# Potential Herb–Drug Interactions in the Management of Age-Related Cognitive Dysfunction

**DOI:** 10.3390/pharmaceutics13010124

**Published:** 2021-01-19

**Authors:** Maria D. Auxtero, Susana Chalante, Mário R. Abade, Rui Jorge, Ana I. Fernandes

**Affiliations:** 1 CiiEM, Interdisciplinary Research Centre Egas Moniz, Instituto Universitário Egas Moniz, Quinta da Granja, Monte de Caparica, 2829-511 Caparica, Portugal; mauxtero@egasmoniz.edu.pt (M.D.A.); susanachalante1@sapo.pt (S.C.); mariorui91@hotmail.com (M.R.A.); rjorge@egasmoniz.edu.pt (R.J.); 2 Polytechnic Institute of Santarém, School of Agriculture, Quinta do Galinheiro, 2001-904 Santarém, Portugal; 3 CIEQV, Life Quality Research Centre, IPSantarém/IPLeiria, Avenida Dr. Mário Soares, 110, 2040-413 Rio Maior, Portugal

**Keywords:** herb–drug interactions, botanicals, food supplements, nootropics, phytochemicals, nutraceuticals, pharmacokinetics, cognitive dysfunction

## Abstract

Late-life mild cognitive impairment and dementia represent a significant burden on healthcare systems and a unique challenge to medicine due to the currently limited treatment options. Plant phytochemicals have been considered in alternative, or complementary, prevention and treatment strategies. Herbals are consumed as such, or as food supplements, whose consumption has recently increased. However, these products are not exempt from adverse effects and pharmacological interactions, presenting a special risk in aged, polymedicated individuals. Understanding pharmacokinetic and pharmacodynamic interactions is warranted to avoid undesirable adverse drug reactions, which may result in unwanted side-effects or therapeutic failure. The present study reviews the potential interactions between selected bioactive compounds (170) used by seniors for cognitive enhancement and representative drugs of 10 pharmacotherapeutic classes commonly prescribed to the middle-aged adults, often multimorbid and polymedicated, to anticipate and prevent risks arising from their co-administration. A literature review was conducted to identify mutual targets affected (inhibition/induction/substrate), the frequency of which was taken as a measure of potential interaction. Although a limited number of drugs were studied, from this work, interaction with other drugs affecting the same targets may be anticipated and prevented, constituting a valuable tool for healthcare professionals in clinical practice.

## 1. Introduction

The aging population and the increased life expectancy have unveiled the need for effectively managing associated cognitive decline to maintain functional capacity and quality of life. Senile dementia is a clinical syndrome observed in the elderly, which includes a range of progressive neurological disorders characterized by a number of cognitive deficits, such perception, logical thought, memory, orientation, and alertness [1]. Dementia and cognitive deficit prevalence is increasing considerably, mostly because old age is the main risk factor [1,2]. In 2015, 47 million people were estimated to be affected by dementia, and the predictions for 2050 amount to 131 million people worldwide [3]. Of note is also that the onset of dementia is occurring increasingly earlier in life [4] and, besides old age, chronic conditions such as diabetes, depression, hypertension, and various forms of vascular disease are also risk factors [2].

Age-related brain disorders, such as dementia and its most prevalent form Alzheimer’s disease, are a burden with limited pharmacological therapies available [5]. Multiple mechanisms have been proposed to underlie the causes of dementia, and therefore there are a variety of potentially valid treatment strategies from the broad concepts of improving angiogenesis and cerebral blood flow [6] or the antioxidant and neuroprotective effect against oxidative stress [7], to more specific targets such as modulating the brain glutamatergic and cholinergic neurotransmission [8] or improving the hippocampal brain-derived neurotrophic factor mRNA levels [9,10]. At present, licensed drugs include cholinesterase inhibitors (donepezil, rivastigmine, and galantamine) and a glutamate NMDA (*N*-methyl-d-aspartate) receptor antagonist (memantine) [4,11]; a range of other nonspecific drugs often prescribed include tranquilizers, antipsychotics, antidepressants, and hypnotics.

The burden of the disease may be reduced by identifying new prevention and treatment strategies [1]. In addition to pharmacological intervention, the aged are resorting to complementary and alternative approaches to delay decline and enhance cognitive function. On one hand, the role of diet in late-life cognition has also been postulated and neuroprotective food supplements considered to delay onset of such disorders [6]. On the other, traditional medicine is considered particularly attractive in treating poor-resources populations [12,13], and the World Health Organization has recognized its important role in primary healthcare in such contexts [14].

Natural products and, in particular, botanicals play a central role in healthcare and management, not only because of their widespread use as food supplements, but also as source of new drugs per se, or as lead compounds [15,16]. Precedents for the continuing search for plants and phytochemicals, which can prevent or treat cognitive impairment, are natural compounds such as galantamine from Amaryllidaceae (e.g., *Galanthus* or *Narcissus*) species [4,17]. Although clinical evidence of efficacy is at times rather inconsistent and scarce, many promising plants (as such or concentrated in the form of extracts) have been identified and extensively reviewed [4,5,18,19,20,21]. Many of these contain bioactive compounds, belonging to different chemical classes, with good to excellent anticholinesterase activity [5,19,20,22,23], or antioxidant and anti-inflammatory effects, among others [19,21].

Additionally, access to online information has dramatically broadened the scope of treatment options, and the elderly are sometimes making their own health decisions, without consulting a physician. As a result, use of herbal food supplements (also called nutraceuticals, highlighting the link between food and health), touted for benefits such as improved memory and concentration (often called nootropics), is on the rise. Though usually considered by consumers as safe and exempt from side effects due to their natural origin, the potential for interaction with drugs is well documented [24,25,26] affecting both efficacy and safety (varying from mild to life-threatening episodes) of drugs.

Considering that dementia’s peak incidence, in developing countries, is among those aged 80–89 years [2,3] and also that polypharmacy often occurs in the geriatric population with pre-existing comorbidities [27], the risk of interactions is increased manifold. A thorough evaluation by the doctor is thus needed to select an intervention with a favorable risk–benefit and prevent the common problems of drug–drug or herbal–drug interactions (HDI) [28]. Understanding pharmacokinetic and pharmacodynamic interactions is warranted to avoid undesirable adverse drug reactions, which result in unwanted side-effects or therapeutic failure.

The aim of this narrative review of the literature is to evaluate potential risks of HDI between purported botanical cognitive enhancers often taken by the elderly and ten representative drugs of different pharmacotherapeutic classes commonly prescribed to this age group. The work is limited to phytochemicals with reported benefits in cognition, disregarding the effect of other constituents of the plants considered.

The ability of mutual (bioactive-drug) target (enzymes, transporters and receptors) modulation (substrates, inhibitors and inducers) was taken as a measure of the interaction potential. This work not only identifies the bioactives with the highest HDI potential, but can also be further utilized as a suitable database for physicians and healthcare professionals to improve clinical outcomes and prevent adverse effects.

## 2. Methodology

**Figure 1:**
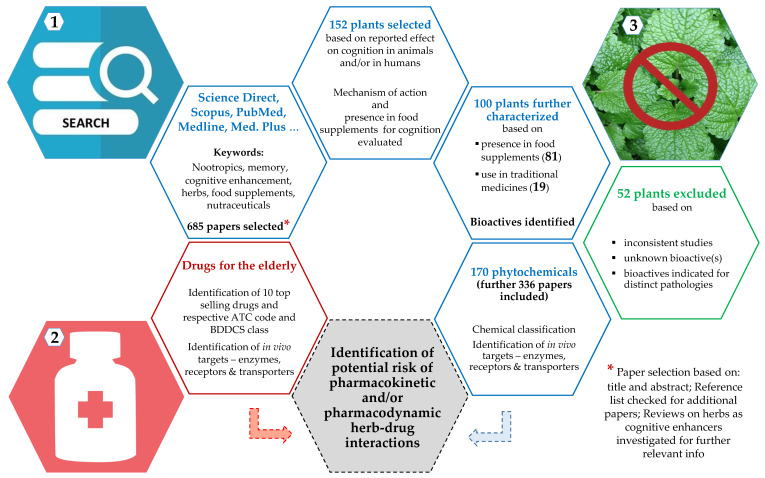
Workflow used to retrieve information to identify potential risk of HDI in aged food supplements’ consumers. Herbals are represented in blue (**1**), drugs in red (**2**), and exclusions in green (**3**). The number of publications used, botanicals characterized, and bioactives identified and studied, is highlighted in bold.

The workflow used to retrieve information is represented in Figure 1 using a color code to distinguish between paths taken regarding plants (blue), drugs (red), and exclusions (green). In short, several online electronic databases (e.g., Scopus, Google Scholar, ScienceDirect, Medline, Medline Plus, and Pubmed) were used to search for relevant literature on the use of plants for cognition related ailments. Keywords, such as nootropics, memory, cognitive enhancement, herbs, food supplements, and nutraceuticals, were used. A total of 685 papers was selected based on title and abstract and thorough checking of reference lists for additional papers. Previous reviews on the use of herbs as cognitive enhancers were also investigated for further relevant information, double checked by another investigator. 

A total of 152 plants were retrieved as having an effect in cognition, through a number of different mechanisms. From those, 100 plants were considered for further characterization and checked for inclusion in official compendia—European Pharmacopoeia (Ph. Eur.) [29], United States Pharmacopoeia (USP) [30], The Japanese Pharmacopoeia (JP) [31], Indian Pharmacopeia (IP) [32], Pharmacopoeia of the People’s Republic of China (PPRC) [33], World Health Organization (WHO) monographs on selected medicinal plants [34], and the Herbal Medicines Compendium (HMC)-USP [35]. Plants (52) not matching the eligibility criteria were excluded. Exclusions were made when studies were inconsistent, or the plant bioactive(s) unknown, poorly studied or indicated for different pathologies.

The plants selected (100) corresponded to 170 bioactives related to cognition enhancement, whose presence in commercial food supplements was also checked. Bioactives were grouped in chemical families and the relative weight of each class determined. For every bioactive, a thorough pharmacokinetic characterization was made regarding action as substrates, inducers, or inhibitors of target enzymes (e.g., cytochrome P450 (CYP), AMP-activated protein kinase (AMPK), monoamine oxidase (MAO), cyclooxygenase (COX), and arachidonate 5-lipoxygenase (ALOX5)), transporters (ATP binding cassette (ABC)), such as P-glycoprotein (P-gP) and MRP (multidrug resistance-associated protein), and solute carriers (SCL) and receptors (e.g., *N*-methyl-d-aspartate (NMDA)), identified as being involved in selected drugs’ disposition.

Ten representative drugs commonly prescribed to the elderly, belonging to 10 pharmacotherapeutic groups, were selected according to unpublished prescription data (2017-2019) supplied by the National Pharmacies Association. They were classified according to the Anatomical Therapeutic Chemical Classification System (ATC) [36], the Biopharmaceutical Drug Disposition and Classification System (BDDCS) [37,38,39,40,41,42,43] and checked for increased risk if an interaction occurs [44]. The pharmacokinetics of each drug was evaluated as described for bioactives.

Finally, the potential risk of pharmacodynamic and/or pharmacokinetic HDI was evaluated by identifying simultaneous action in the same target(s) and the number of mutual targets affected, used as a measure of the probability of interaction occurrence.

## 3. Botanicals for Cognitive Enhancement

### 3.1. Identification and Selection of Botanicals (and Respective Bioactives) Implicated in Cognition Enhancement

Botanicals (whole plant or parts of the plant), their extracts, or isolated bioactives, were identified through an online search, as detailed in Section 2. The plants considered for additional study and the main bioactive molecules reported as responsible for enhancing cognition are presented in Table 1. Plants excluded, typically due to lack or inconsistent evidence of efficacy in cognition, unknown bioactive, or toxicity, are shown in the footnote of the same Table. As an example, *Albizia adianthifolia*, despite the antioxidant and acetylcholinesterase (AChE) inhibitory activities shown [45,46] (therefore with potential to manage memory loss and neurodegenerative disorders), has been disregarded. In fact, little is known about the specific function and pharmacokinetic properties of the more than 90 secondary metabolites, isolated from several parts of the plant, including those of a new triterpenoid saponin (adianthifolioside J) recently identified [47]. *Ricinus communis* was also excluded, because the level of evidence for its use in cognition-related diseases is poor, despite the fact that its bioactive, ricinine, was considered a central nervous system stimulant [48] and a promising cognition-enhancing drug [49].

### 3.2. Mechanisms of Action in Cognition Enhancement

Herbals in food supplements present substantial variability in composition according to ecotype, culture conditions, harvesting season, extraction method, and other processing operations. Moreover, the high complexity of the plant matrices and the multiplicity of compounds they contain (sometimes with synergic or antagonic action) may also contribute to contradictory and inconsistent findings. It is therefore not uncommon to find literature reports that point in different directions. A thorough description of the multiple mechanisms by which plants can improve cognition is out of the scope of this work and only a simplified overview is provided.

Of the plants studied, 63% are listed in at least one of the official compendia consulted and presented in Table 1. Ph. Eur., for example, lists 37 plants, 10 of which are part of a general chapter dedicated to herbal drugs used in Traditional Chinese Medicine, published for information only.

**Figure 2:**
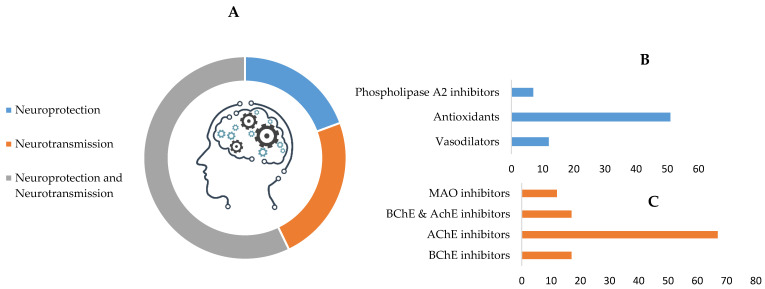
Summary of the main mechanisms by which botanicals may enhance cognition (**A**). Main relative contributions to neuroprotection (**B**) and neurotransmission (**C**) are highlighted. AChE—acetylcholinesterase; BChE—butyrylcholinesterase; MAO—monoamine oxidase.

The main mechanisms identified in these plants, as associated with cognition enhancement, relate either to neuroprotection, neurotransmission, or a combination of both (Table 1 and Figure 2A).

Neuroprotection is associated with radical oxygen scavenger ability, reduction in inflammation and associated brain damage. Polyphenols, for instance, play an important part in reducing oxidative stress-induced inflammation and associated diseases. In fact, inflammation plays an important role in age-related cognitive disorders [50] and, as such, antioxidative molecules and the inhibitors of pro-inflammatory enzymes or cytokines, present in many (58%) of the plants considered, may improve cognition. As examples, *Bacopa monnieri* [51] has been associated with a reduction of radical oxygen associated inflammation and plants, such as *Foeniculum vulgare* [52] and *Centella asiatica* [53], inhibit the production of phospholipase A2. Crocin from *Crocus sativus* suppressed formation of brain inflammatory mediators, such as interleukin-1 and tumor necrosis factor-α [54].

On the other end, β-amyloid is a component of amyloid plaques characteristic of Alzheimer’s, and T-tau and P-tau proteins are over phosphorylated in neurodegenerative disorders [50]. Inhibitors of aggregation/destruction of β-amyloid plaques or protection of T-tau and P-tau proteins (e.g., *Alpinia oxyphylla* [61]) were considered within the neuroprotective group, together with plants which reportedly prevent neuronal death (e.g., *Schisandra chinensis* [174]).

There is a strong link between reduced vasodilation (which can result in cerebrovascular lesions) and cognitive impairment and, ultimately, vascular dementia in elderly people [175]. Vasodilator-containing plants improve blood flow to the brain, reducing ischemia and therefore protecting the brain from injury and ameliorating cognition. *Angelica sinensis* [63], *Eleutherococcus senticosus* [91], and *Salvia miltiorrhiza* [24] are examples of such plants. Vinpocetin present in *Vinca minor* is also used as a neuroprotective cerebral vasodilator [20] and may interact with warfarin and other anticoagulants [176].

Many of the plants studied impact directly on neurotransmission (79%) by inhibiting acetylcholinesterase (AChE), butyrylcholinesterase (BChE), and MAO (Figure 2C). Others inhibit catechol-O-methyltransferase (COMT; the enzyme responsible for the degradation of the catecholamine neurotransmitters) or show cholinergic activity (e.g., Z-ligustilide and ferulic acid from *Angelica sinensis* [18]). Noteworthy is the significant expression of plants capable of inhibiting AChE alone (67%) or in conjunction with BChE (17%), the majority of which contain alkaloids (e.g., assoanine, galantamine, lycorine, sanguinine and huperzine) and show promise in the treatment of Alzheimer’s disease [17,22]. *Pancratium illyricum* roots and leaves are examples of good sources of anticholinergic alkaloids, including a particularly potent molecule, 11α-hydroxy-*O*-methylleucotamine [121]. Huperzine A is an example of a lead compound in the development of anticholinesterase drugs, which has shown higher oral bioavailability and blood–brain barrier penetration, as well as longer duration of activity, as compared to the approved drugs [22].

Quercetin and β-carbolines (e.g., harmine) from *Mucuna pruriens* and *Peganum harmala* strongly inhibit MAO [126]. Antidepressant and anxiolytic activity have also been associated with *Rhodiola rosea* [148], *Hypericum perforatum* [177], and *Rosmarinus officinalis* [178], amongst many other plants.

Glutamine is an excitatory neurotransmitter, an energy substrate, the precursor of the neurotransmitter amino acids glutamate of γ-aminobutyric acid (GABA), as well as a potent neurotoxin [179]. As such, glutamate homeostasis is paramount, and several plants (e.g., *Hypericum perforatum* [98] and *Mangifera indica* [108]) reportedly regulate glutamate signaling through NMDA receptor antagonism.

Perhaps the most striking plant for its levodopa contents is *Mucuna pruriens*, whose therapeutic utility of the many seed constituents in neuroprotection and treatment of Parkinson’s disease has been reviewed by Kasture [12].

Estrogen-like effects of phytochemicals (e.g., biochanin A, which requires P450-catalyzed metabolism to generate the active phytoestrogens daidzein and genistein, in *Trifolium pratense* and isoliquiritigenin in *Glycyrrhiza uralensis*) may also contribute to reduced cognitive decline and improve cerebrovascular function in postmenopausal women [158,180].

Caffein, present in *Paullinia cupana* and *Coffee arabica,* is a central nervous system stimulant and an adenosine receptor antagonist, increasing acetylcholine and dopamine transmission in the brain [181], which acts as an energizer, reducing fatigue and promoting wakefulness. In addition, the structurally similar alkaloids, theacrine and methylliberine, also identified in the seeds and leaves of *Coffee arabica* [74], are believed to potentiate and synergize with caffein, also enhancing mood, energy, focus, and motivation, but showing less side effects. *Camellia sinensis* catechins (epigallocatechin-3-gallate and epicatechin gallate) present antioxidant and anti-inflammatory activity and are capable of crossing the blood–brain barrier, acting as neuroprotectors [73].

Additionally, plants often present vitamins (e.g., A, B1, B2, B3, B12, C and E), as well as minerals, including calcium, zinc, potassium, copper, manganese, sodium, and iron, among others. These act, for instance, as antioxidants and enzyme cofactors, also contributing to cognition enhancement.

As a result of the different mechanisms described, plants (and bioactives) are frequently categorized based on their target application, such as mood support, improved mental focus, alertness and memory, stress reduction, neurostimulation, antidepressants, anxiolytics, anti-Parkinsonians, etc.

## 4. Chemical Characterization of Nootropic Bioactive Compounds

Phytochemicals are plant-derived bioactive, non-nutrient chemicals, which can be found in plant foods (e.g., fruits, vegetables and grains) and food supplements. They encompass a group of secondary metabolites and are part of the plant’s adaptation mechanism to the environment. Phytochemicals are responsible for the health benefits attributed to botanicals and the purported prevention or risk reduction of chronic diseases, such as dementia, Alzheimer’s, or Parkinson’s.

**Figure 3:**
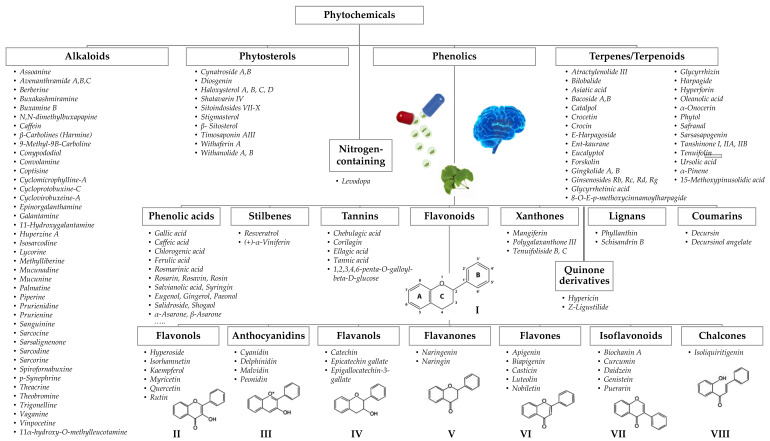
Classification of phytochemicals found in botanicals used in cognitive enhancement. For clarity, compounds, which due to the reduced expression were grouped as “others”, are not represented; in a similar fashion, a shortened list of phenolic acids and only the aglycone part of flavonoids are shown. The structural backbone of flavonoids (**I**) comprises two phenyl rings (**A**,**B**) and a heterocyclic ring (**C**); the flavonoid family is classified into different groups, such as flavonols (**II**), anthocyanidins (**III**), flavanols (**IV**), flavanones (**V**), flavones (**VI**), isoflavonoids (**VII**), and chalcones (**VIII**), according to the degree of oxidation and substituent chemistry.

**Figure 4:**
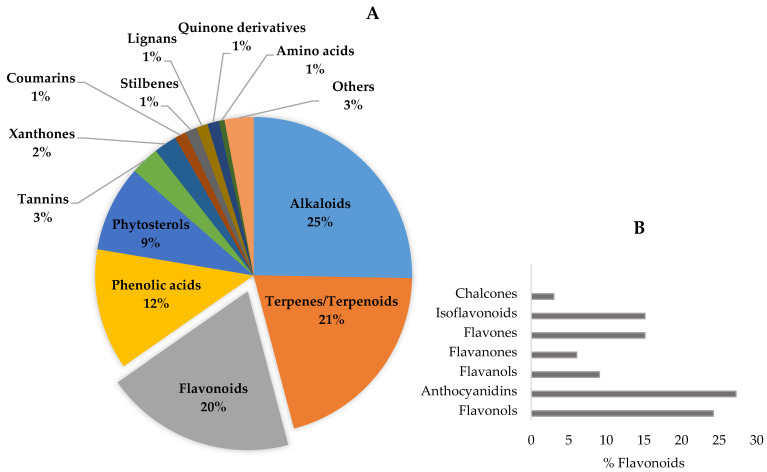
Phytochemicals in botanicals for cognitive enhancement (*n* = 170) by chemical class, evidencing the contribution of flavonoids (**A**). Phenolic compounds represent 42% of the total. On the inset (**B**), the relative percentage of the different types of flavonoids is presented.

The bioactive compounds identified in botanicals for cognitive enhancement, as described in the previous section, were classified according to their chemical structure [182,183,184,185] (Figure 3). Phytochemicals may also be categorized according to their different functions in the body, such as antioxidants, anti-inflammatory, neuroprotective, etc., reflecting their mechanism of action, previously discussed. Mixed classifications are sometimes found in literature. Noteworthy is the relative contribution of alkaloids (25%), terpenes/terpenoids (21%), flavonoids (20%), and phenolic acids (12%), as shown in Figure 4A. Compounds with reduced expression were grouped as “others” (Figure 4A), except for the amino acid levodopa, which, due to its relevance, is emphasized. Again, quinone derivatives (e.g., hypericin, a naphtodianthrone and Z-ligustilide, a benzoquinone derivative; Figure 3) and the family of flavonoids are highlighted (Figure 4A) and detailed in Figure 4B due to their outstanding brain health-promoting potential.

Alkaloids are a heterogeneous group of naturally occurring chemical compounds, the majority of which contain nitrogen, usually in a heterocyclic ring [186]. Many are toxic and marketed as drugs. Reported actions, which may improve cognition, include antinociceptive, anticholinergic, sympathomimetic, anti-inflammatory, and antioxidant activities, as well as the ability to stimulate the central nervous system and cerebellum, have been extensively reviewed by Debnath et al. [187]. Many of the alkaloids found in our study show anticholinergic activity (56%), in line with reports by other authors [22,188].

Terpenes/terpenoids are the second most prevalent type of bioactives found (Figure 4A). Terpenes are water insoluble and made from single or multiple isoprene units joined together in different combinations to produce a variety of compounds [189]. Terpenoids derive from terpenes, usually by oxidation, and the terms are often used interchangeably. These are strong-smelling compounds and the major components of essential oils. Terpenes show psychoactive [190], anti-inflammatory, and antioxidant effects but some, in particular monoterpenes, are cytotoxic [191]. In plants, the majority of non-alkaloid AChE inhibitors are terpenoids; α-pinene from *Salvia lavandulaefolia* and tanshinones from *Salvia miltiorrhiza* are examples of such compounds [23].

Phenolic compounds represent 42% of the total bioactives identified (Figure 4A) and include simple phenols (e.g., phenolic acids—either hydroxycinnamic or hydroxybenzoic acids—and coumarins) and polyphenols (e.g., tannins, stilbenes, and flavonoids, which are the most representative group). As an example, decursin, a coumarin from *Angelica gigas*, has shown cholinesterase inhibitory activity [23].

Flavonoids show anti-inflammatory properties and reduce oxidative stress, among other direct roles on cognition [192]. Along with carotenoids (tetraterpenes; e.g., crocetin), flavonoids are responsible for the vivid colors of fruits and vegetables. They are found in plants in the aglycone form, polymerized (procyanidins), or linked to sugars in different positions, such as glycosides [15]. The general chemical structure of flavonoids is presented in Figure 3. A relation between structure and activity is possible to establish and, furthermore, the number and type of the sugar residues (glycone) impact oral bioavailability. 

Naringenin (and its precursor naringin), is a flavanone, present in citrus and grapefruits, involved in different signaling pathways mainly related to neuroprotection [193]. Naringin attenuates inflammatory response (its potential to alleviate COVID-19 symptoms has recently been reported [194]), and it is believed to show anti-AChE activity as well [195]. The use of naringenin is, however, compromised due to poor oral bioavailability and accessibility to the brain [193].

Phytosterols are structurally related to cholesterol and encompass plant sterols and stanols, mainly present in vegetable oils, nuts, and cereals [196]. They have been linked to cholesterol lowering properties, but are also present in nootropic food supplements. Withaferin A and withanolides A and B, from *Withania somnifera*, are examples of neuroprotective sterols [165].

Our literature search revealed the presence in botanicals (e.g., *Mucuna pruriens*) of one amino acid well known for its activity in cognition: levodopa (L-DOPA), a precursor of dopamine with antiparkinsonian properties [12].

## 5. Interactions between Botanicals and Drugs

HDI are either pharmacokinetic, i.e., related to drug disposition, or pharmacodynamic, i.e., caused by changes in the drug’s mechanism of action. Nonetheless, pharmacokinetic interactions are the most frequent. Only a brief summary is provided, since detailed characterization of drug targets is out of the scope of the present work.

### 5.1. Protein Targets as Key Points for Herb–Drug Interactions

Plants and herbal formulations contain several bioactive compounds, which increase the likelihood of HDI occurring with prescribed drugs, as reported in clinical practice [197]. On the other hand, drug’s pharmacokinetic phases (absorption, distribution, metabolism, and elimination; ADME) require drug transformation and/or membrane crossing with the involvement of specific proteins, such as members of the CYP450 superfamily, and drug carriers.

HDI occurs when a botanical formulation interferes with the action of a co-administered drug. This can happen by action on several targets, such as enzymes, receptors, and transporters, causing changes in the drug’s plasma profile, which can compromise therapeutic success or have fatal consequences, especially with narrow therapeutic margin drugs (HAM, as discussed before).

#### 5.1.1. Cytochrome P450

CYP450 refers to a large family of enzymes responsible for the phase I metabolism of most drugs and other chemical compounds. CYP450 enzymes are grouped in families, with CYP1, CYP2, and CYP3 metabolizing the majority of xenobiotics. Although these enzymes can be found in several tissues, they are more abundant in the liver and small intestine. CYP3A, the most abundant, is implicated in many drug interactions [198].

Regarding the drugs evaluated in the present work, 70% are substrates of CYP3A (mainly CYP3A4, but also CYP3A5 and CYP3A7). Based on in vitro, in silico, and in vivo studies largely documented [199], it is fairly safe to conclude that these enzymes are easily vulnerable to modulation by several compounds, including phytoconstituents. Indeed, many of the bioactives isolated from herbs may act as substrates, inducers, or inhibitors of several CYP enzymes. Hence, it is of the utmost importance to identify the enzymes targeted by these bioactives.

#### 5.1.2. Uridine Diphosphate-Glucuronosyltransferases

Drug metabolism may include phase II conjugation reactions mediated by enzymes of the uridine diphosphate-glucuronosyltransferases (UGT) family, using UDP-glucuronic acid as a co-substrate. This conjugation ultimately facilitates drug elimination in urine or bile by increasing its hydrophilicity. UGT members are liable to undergo induction or inhibition by various xenobiotics, such as flavonoids, with a consequent change in the pharmacokinetic profile (e.g., elimination half-life) [200].

#### 5.1.3. Drug Carriers

A large number of drugs and other xenobiotics are organic anions or cations, and their pharmacokinetic disposition depends on special carriers. Drug carriers or transporters are the largest group of membrane proteins in the human body, which ensure the passage of molecules across membranes. The transporters are divided into two main families: ABC and SLC. Although many of the members can perform bidirectional transport, mostly ABC transporters mediate the efflux of drugs, whereas SLC are involved in the substrate uptake and are responsible for the cellular entry of many clinically important drugs. Both are expressed in various tissues, such as in the intestine, where they modulate absorption, in the liver and kidney, influencing the metabolism and excretion of drugs. 

The two main ABC efflux pumps are multidrug resistance protein 1 (MDR1; P-gP) and BCRP. Both proteins limit the entry of several drugs (especially BDDCS Classes II-IV) in the central nervous system and have the potential to alter drug pharmacokinetics. BCRP serves two major drug transport functions, conditioning the distribution of its substrates into several organs, such as the brain, and eliminating its substrates from excretory organs.

SLC includes two superfamilies responsible for the transport of organic anions and cations: SLC21A (current designation, solute carrier organic anion transporter family, SLCO), comprising the organic anion transporting polypeptides (OATP), and SLC22A, which contains the organic anion/cation transporters (OAT/OCT) [201,202,203].

Following recommendations of the International Transporter Consortium (ITC) on transporters with relevance in drug interactions [204], the main transporters with impact on drug ADME are P-gP, BCRP, OATP1B1/1B3/2B1, OCT1/2, SLC47A, MRP, and bile salt export pump (BSEP). Hence, these are more likely to be involved in herb–drug or drug–drug interactions.

#### 5.1.4. Other Targets

In addition to drug carriers UGT and CYP450 oxidative enzymes, which have a significant influence on pharmacokinetics of administered drugs, other targets may also be involved in HDI through pharmacodynamic processes, such as COX and MAO enzymes and NMDA receptor. 

COX1 and COX2 catalyze the formation of prostaglandins, thromboxane, and levuloglandins. COX enzymes are clinically important, because they are inhibited by non-steroidal anti-inflammatory drugs, such as Di, also used as antipyretic and antithrombotic [205]. Therefore, the bioactives under evaluation, which exert an inhibitory or inductor effect on this group of enzymes, have the potential to affect the therapeutic efficacy of Di, through a HDI that may increase the risk of side effects [206].

MAO (A and B) is a widely distributed mitochondrial enzyme with high expression levels in gastro-intestinal and hepatic, as well as neuronal, tissues. The enzyme catalyzes the oxidative deamination of a variety of monoamines, both endogenous and exogenous, and has major roles in metabolizing released neurotransmitters and in detoxification of a large variety of endogenous and exogenous amines [207]. Whenever drugs and bioactives, taken concomitantly, share MAo-A and/or MAo-B as targets (e.g., Se, as substrate and Pr, as inhibitor), an HDI may occur with impact on the deamination of monoamines and the metabolization of neurotransmitters. Especially, the upregulation of MAo-A prompted increments of 5-hydroxyindoleacetic acid/5-hydroxytryptamine ratio (5-HIAA/5-HT) and oxidative stress, leading to nuclear factor-κB activation, inflammation, and apoptosis [208].

NMDA receptor is a ligand of glutamate, the primary excitatory neurotransmitter in the human brain. It plays an integral role in synaptic plasticity, which is a neuronal mechanism believed to be the basis of memory formation. NMDA receptors also appear to have involvement in a process called excitotoxicity, which may play a role in the pathophysiology of a variety of diseases such as Alzheimer’s disease. Many drugs inhibit NMDA receptors, including Me, an uncompetitive NMDA antagonist, which is used in the treatment of Alzheimer’s and off-label for Huntington’s diseases [209]. Bioactive compounds, which are antagonists and inhibit NMDA receptors, can mimic Me activity.

### 5.2. Drugs Used in Elderly Patients

Aging is associated with an increase in chronic pathologies and, consequently, an increase in medication. Indeed, the number of elderly people who regularly take five or more medications (polypharmacy) has been rising in several countries. In the 2017 study by Page et al. [210], it was found that 36.1% of Australians over 70 years of age were polymedicated with five or more medications, representing about one million people. In the USA, between 2013 and 2016, the value rose to 40.9%, for people older than 65 [211]. Polypharmacy increases the risk of drug related interactions, which leads to clinical complications with significant damage to the patient and financial loss.

Propranolol (Pr), alprazolam (Al), sertraline (Se), metformin (Mt), diclofenac (Di), atorvastatin (At), tadalafil (Ta), memantine (Me), piracetam (Pi), and clopidogrel (Cl) were selected as representatives of the pharmacotherapeutic classes commonly prescribed in this age group.

Pharmacokinetic and pharmacodynamic processes are influenced by the transport of drugs through membranes and, eventually, by metabolism [212]. Crossing of membranes can occur either by passive diffusion or by active or facilitated transport mechanisms involving transporters. Metabolism, on the other hand, results from the action of enzymes. Transporters and enzymes are found essentially in the intestinal epithelium, liver, and kidneys, and can exist in many other tissues, such as the brain and heart. Changes in the expression and/or activity of transporters and enzymes can result in modification of the disposition of drugs with a compromise in effectiveness and safety [213].

Detailed knowledge of drug pharmacokinetics, especially of the involvement of targets such as enzymes, transporters, and receptors, allows understanding, anticipation, and prevention of interactions with other xenobiotics, such as phytochemicals [214]. Nevertheless, not all interactions may have clinical relevance, and to assess the real significance of each enzyme or transporter can be a lengthy and expensive process [215], hence the need for a simplified method to define whether enzymes and transporters are potentially important in the clinic [216].

BDDCS was developed to predict drug disposition and potential drug–drug interactions, mainly in the intestine and the liver [40]. The system classifies drugs based on the criteria of solubility and permeability, in order to establish the relevance of enzymes and transporters in determining drug disposition. For example, according to BDCSS, At is a Class II drug (exhibiting poor solubility and extensive metabolism), which may potentially exhibit an interaction with inhibitors of hepatic uptake transporters. In fact, as indicated in Table 2, the disposition of At involves several CYP enzymes and transporters. 

Drugs in Class I and II have a disposition greatly influenced by metabolism (>70%), whereas classes III and IV drugs are mainly eliminated unchanged [43]. In short, these authors hypothesize that Class I drugs are very affected by enzymatic changes, but not by changes in transporters. On the other hand, Class II drugs can undergo major changes in disposition due to enzymatic and transport modifications. Class III drugs are unlikely to be affected by metabolic changes, but are susceptible to changes in absorption or efflux transport in various tissues. Finally, Class IV drugs (not represented in the drugs selected amongst the most prescribed, probably due to the fact that they represent about 5% of the approved drugs [38]) are substrate for P-gP and undergo extensive presystemic metabolization. Noteworthy is that BDDCS only allows for predictions, i.e., there will always be drugs with unanticipated behavior.

The clinical significance of changes in drug disposition is also dependent on the type of drug. High-Alert Medications (HAM) bear a significant risk of causing harm to patients if errors or interactions occur, thus requiring extra caution. These drugs present narrow therapeutic indexes, and therefore, small changes in drug blood levels can result in critical, even life-threatening events.

Table 2 summarizes the different targets involved in the metabolism, transport, and action of the selected drugs and includes their BDDCS and HAM classifications. Details of the targets involved in the pharmacokinetics of the 10 drugs studied can be found in the Supplementary Material.

Generally, drugs act as substrates of enzymes and drug carriers. The top six targets are four enzymes of CYP P450 (CYP3A4, CYP3A5, CYP2C9, and CYP2C19) and two efflux pumps from the ABC transporters family (P-gP and breast cancer resistance protein-BCRP). CYP3A4 metabolizes all the Class I and Class II drugs (Pr, Se, Al, At, Cl, Di, and Ta); CYP3A5 is involved in the metabolism of Pr, Al, At, Ta, and Cl and CYP2C19 metabolizes Pr, Al, Se, Di, and Cl, whereas it is inhibited by At and Me; CYP2C9 is induced by At and metabolizes Se, Al, Di, and Cl; with respect to transporters, Pr, Se, At, Ta, and Cl are substrates of P-glycoprotein, and Di induces its expression. Finally, Se, Mt, Di, and At are substrates of BCRP transporter, which is inhibited by Cl. Other enzymes are also inhibited by the drugs, such as CYP2B6 (Se, Me, and Cl) and CYP2D6 (Pr, Se, and At).

Regarding uptake transporters, such as the solute carrier family, there are several members involved in drug disposition. For example, organic-anion-transporting polypeptide (OATP) OATP1B3 (SLC21A8) is an uptake transporter exclusively expressed in the liver on the basolateral side of hepatocytes. Together with OATP1B1 (SLC21A6), it is responsible for the hepatic uptake of some important drug classes, including the BDDCS Class II, At, Cl, and Di, thus mediating drug interactions.

SLC22A2 (organic cation transporter 2-OCT2) facilitates the transport of cationic compounds, including many drugs such as Mt. SLC22A2 is inhibited by four of the 10 drugs studied (Pr, Mt, Me, and Cl).

**Figure 5:**
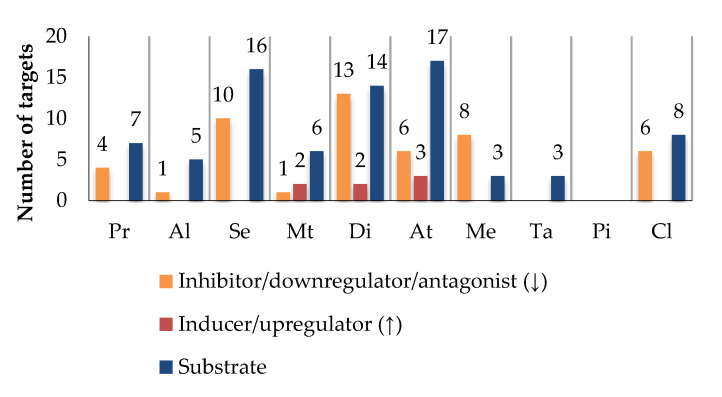
Effect of drugs on targets. The number of shared targets between drugs and bioactives was taken as a measure of potential interaction. Propranolol (Pr), alprazolam (Al), sertraline (Se), metformin (Mt), diclofenac (Di), atorvastatin (At), tadalafil (Ta), memantine (Me), piracetam (Pi), and clopidogrel (Cl).

Di, Se, and At are the most promiscuous drugs, being related to 29 (14, as a substrate), 26 (16, as a substrate), and 26 (17, as a substrate) targets, respectively (Figure 5). In addition, At and Di are HAM and are classified as Class II drugs, making their disposition more likely to depend on both enzymes and transporters. Thus, these drugs have a higher risk of clinically relevant interactions with bioactive agents, which share the same targets. On the other hand, Pi is the drug with the lowest probability of HDI, since it does not share any target with the phytochemicals under study.

### 5.3. Target Modulation by Bioactives

Given the importance of the above mentioned entities as potential targets for HDI and considering the fact that all of them are somehow involved with the drugs under study, a literature review was undertaken, in order to characterize the relationship between each of the bioactives isolated from the plants used for cognitive enhancement and neuroprotection and those targets. A total of 55 targets, including drug carriers, receptors (e.g., NMDA), UGT, CYP, and other enzymes (e.g., MAO, COX, ALOX5, 3-hydroxy-3-methyl-glutaril-CoA reductase-HMGCoAR) were analyzed, and the results are displayed on Table 3.

**Figure 6:**
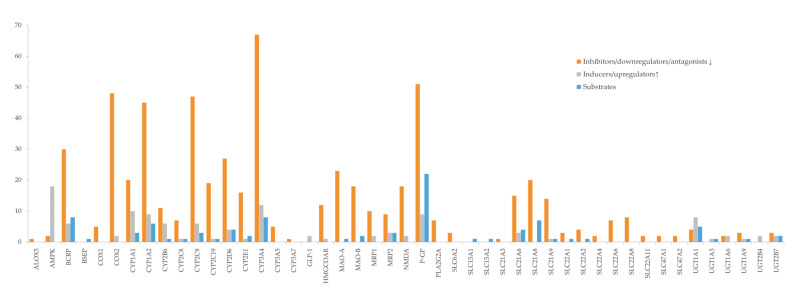
Effect of bioactives on targets (enzymes, transporters, and receptors).

Bioactives interfere in the targets, mainly by inhibition (Figure 6), and the six most frequently inhibited are CYP3A4 (*n* = 67; 39%), P-gP (*n* = 51; 30%), COX2 (*n* = 48; 28%), CYP2C9 (*n* = 47; 28%) and CYP1A2 (*n* = 45; 26%), and BCRP (*n* = 30; 18%). These are of particular importance for the pharmacokinetic profile (or pharmacodynamic in case of COX2) of multiple drugs, as it was also observed for the majority of the drugs under study. Moreover, P-gP and BCRP are amongst the most relevant transporters for drug interactions [204], and CYP enzymes, particularly 3A and 2C families, play a major role in the disposition of many drugs and have been associated with HDI [245].

**Figure 7:**
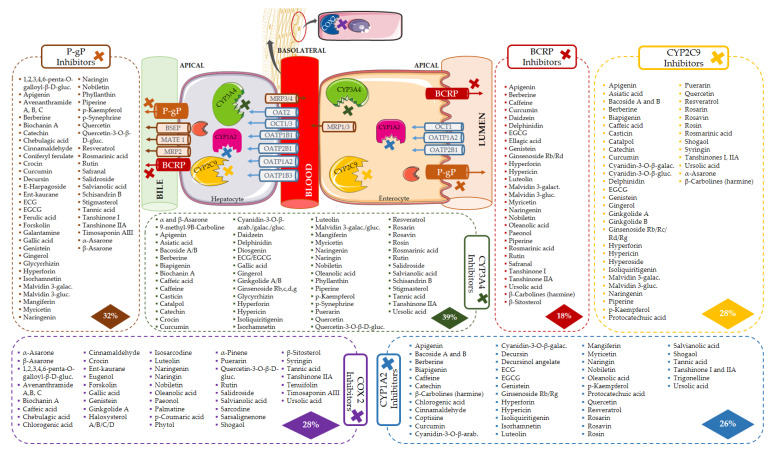
Bioactives that inhibit the six most affected targets (COX2, CYP1A2, CYP3A4, CYP2C9, BCRP, and P-gP). Location of the targets is indicative, since they are expressed in several other tissues.

The bioactives responsible for the inhibition of the six most affected targets are shown in Figure 7. The location of the targets is purely indicative, since they are expressed in several other tissues.

By comparison of the type of interaction that drugs and bioactives have on the 55 targets analyzed, a high degree of overlap is evident, with drugs acting mostly as substrates of enzymes and transporters, whereas bioactives act as inhibitors of the same targets. As an example, from the top six targets of drugs and bioactives, four are shared: two enzymes of CYP P450 (CYP3A4 and CYP2C9) and two efflux pumps from the ABC transporter family (P-gP and BCRP). Hence, whenever drugs and herbal formulations are associated, the modulation that bioactive compounds can exert on targets may lead to therapeutic failure or toxicity.

The number of targets affected by the herbal bioactives largely depends on the type of the latter. On the one hand, one fourth of the 170 bioactives studied did not show any influence in any of the 55 targets (see Supplementary Material). On the other hand, some have the capacity to modulate several different targets. Naringenin, for example, modulates 20 targets (13 inhibitions and seven inductions), while epigallocatechin-3-gallate (EGCG) and quercetin both affect a total of 19 targets, mainly by inhibition. The higher the number of targets affected, the higher the potential for interaction.

### 5.4. Assessment of HDI Potential

In order to understand the potential consequences of combining each bioactive with the drugs, a crossed analysis was performed searching for matches between the targets of which the drugs are substrate and that are simultaneously inhibited, or induced, by the bioactive. Whenever a match was found in at least one target for a specific drug and a specific bioactive, the latter was considered a potential HDI agent, regardless of the direction of modulation (inhibition or induction). Otherwise, if the roles of the drug and the bioactive were reversed, that is, the drug assumed the role of inhibitor/inducer of a specific target of which the bioactive was a substrate, the interaction was considered to be of a different nature, because, in this case, it is the drug that changes the disposition of the bioactive. However, this type of interaction was disregarded in the present study.

Furthermore, situations were identified in which the drug and the bioactive modulate the same target, either in the same direction (both inhibit/induce) or in opposite directions (one induces and the other inhibits the target).

**Figure 8:**
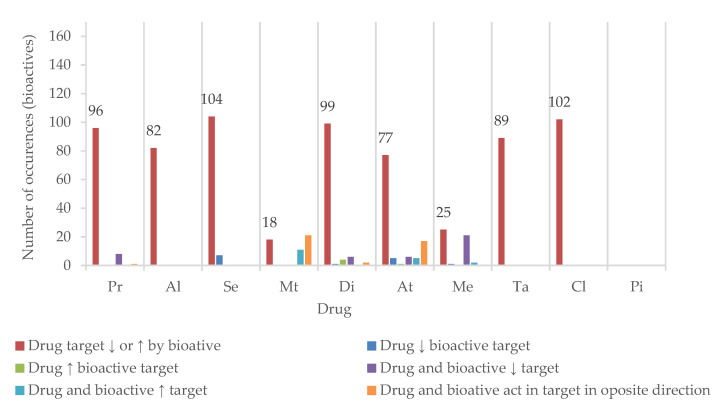
Number of bioactives sharing at least one target with the drugs (↓—inhibition; ↑—induction). Propranolol (Pr), alprazolam (Al), sertraline (Se), metformin (Mt), diclofenac (Di), atorvastatin (At), tadalafil (Ta), memantine (Me), piracetam (Pi), and clopidogrel (Cl).

Half of the drugs have at least one of their targets inhibited or induced by more than 80 of the bioactives found in plants used for cognition enhancement. Se and Cl have over 100 bioactive agents as potential modulators of their metabolism, transport, or therapeutic action (Figure 8).

However, since the risk of HDI is naturally related to the number of shared targets between a given bioactive and a specific drug, only the bioactives that cause induction/inhibition of at least four targets were selected for more detailed analysis. The potential HDI of these with the 10 drugs under study is summarized in a double entry table (Table 4). Hence, a total of 75 bioactive agents met the inclusion criteria (minimum of four targets affected), and 95 were excluded. Of the 95 excluded, only 42 have no action on any of the 55 targets analyzed. Thus, the exclusion of bioactives does not guarantee the absence of interactions with any of the drugs under study; simply, the probability of their occurrence was considered lower. Pi was excluded from the detailed analysis for not sharing any target with any bioactive (Figure 8).

For the construction of Table 4, the targets shared between each drug and each bioactive were analyzed, based on two sequential criteria: (1) targets of which the drug is a substrate; (2) targets of which the drug is a modulator. If at least one target fulfilled criterion 1, the interaction was considered and identified in the table with an x, regardless of inhibition or induction; when no target met criterion 1, the analysis proceeded to targets modulated by the drug (criterion 2), considering the following types of interaction: (a) the drug and bioactive modulate the target in the same or opposite directions and (b) the drug modulates at least one target of which the bioactive is a substrate. For clarity, the following example illustrates application of type 2 criteria: both Di and eugenol are inhibitors of COX2 and Di inhibits CYP2E1, of which eugenol is a substrate; therefore, Di inhibits the metabolism of the bioactive and was identified with ▼. Finally, when none of the criteria were met, it was considered that there was no interaction.

**Figure 9:**
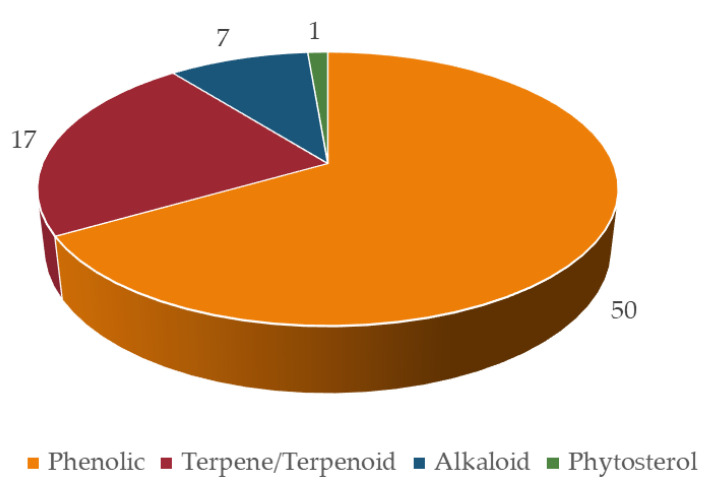
Number of bioactives inducing/inhibiting at least four targets, according to chemical class.

In terms of chemistry, the majority of bioactives with potential to cause HDI belong to the phenolic group (50; 67%), followed by terpenes (17; 23%) and alkaloids (7; 9%) (Figure 9). Phenolic compounds, such as the isoflavonoids (daidzein, genistein, biochanin A, etc.) have already been reported as inhibitors of several CYP enzymes (e.g., the noncompetitive inhibition of CYP2C9 caused by genistein and daidzein [245]). Both flavonoids and terpenoids have the ability to modulate ABC transporters [192,617], which can be advantageous for drugs with poor absorption, but can also lead to toxic plasma drug concentrations, especially for narrow therapeutic window drugs.

Amongst the 50 phenolic compounds listed in Table 4, apigenin, EGCG, genistein, hypericin, quercetin, caffeic acid, catechin, cinnamaldehyde, curcumin, delphinidin, luteolin, naringenin, puerarin, rosmarinic acid, and resveratrol show the capacity to interfere with all nine drugs. For the remaining groups, the terpenoids forskolin, ginsenoside Rd, hyperforin, and ursolic acid and the alkaloids coptisine and piperine showed similar capacity. On the other hand, β-sitosterol only has the potential to modulate disposition of three of the nine drugs (Mt, Di and At). The effect of β-sitosterol on Se disposition was not considered, since the target affected is BCRP, and Se is a BDDCS class I drug and therefore unlikely to depend on efflux carriers. The noncompetitive inhibition of CYP2C9 by genistein can change the disposition of Al, Se, Di, and Cl. Aside from this effect on CYP2C9, genistein modulates 16 other targets, inhibiting for instance CYP1A1/2, COX2, HMGCoAR, BCRP, P-gP, MAo-A, and MAo-B, among others.

Some drugs may also potentiate or antagonize the modulation of bioactives acting on shared targets. For example, At induces CYP2C9, whereas the same CYP is inhibited by biapigenin, catechin, cyanidin-3-*O*-β-glucoside, ginkgolide A, ginkgolide B, ginsenoside Rg, protocatechuic acid, rosarin, rosavin, and rosin. On the other hand, Mt significantly increases glucagon-like peptide-1 (GLP-1) levels, an effect that can be potentiated by forskolin. In the case of At, a drug used to slow the production of cholesterol in the body by inhibiting HMG-CoAR, several of the bioactives studied (e.g., β-sitosterol, rutin, resveratrol, naringenin genistein, chlorogenic acid, oleanolic acid, luteolin, catalpol, and α-asarone) also inhibit the same enzyme. Finally, a drug may cause changes on the disposition of the bioactive by modulating targets of which the latter is a substrate. This is the case of Di, which inhibits CYP2E1, an enzyme involved in the metabolism of eugenol and schisandrin B. Se inhibits CYP1A2, which metabolizes palmatine, daidzein, and paeonol.

As discussed before, drugs belonging to BDDCS class I (Pr, Se and Al) are more sensitive to changes in CYP, whereas class II drugs (At, Cl, Di and Ta) are affected by both CYP and drug carriers; for drugs in classes III and IV, modulation of CYP has a minimal effect [43]. Hence, the disposition of class II drugs may be at greater risk of being modified by co-administration of herbal formulations. On the other hand, for class I drugs in which the only targets shared with bioactives are transporters, the probability of the interaction having clinical significance is low, because, although the drug can use the transporter, its disposition does not depend on it. This is the case of Se combined with decursin or β-sitosterol, where the sole interaction point is the inhibition of P-gP by decursin and the inhibition of BCRP by β-sitosterol. In these cases, the risk of significant interaction is considered low. Likewise, if an interaction is identified between a bioactive and a class III drug, exclusively due to CYP modulation, the risk of clinical significance is low. This was not the case for any of our two BDDCS class III drugs (Mt and Me), since none of them were reported to be a substrate of any CYP enzyme.

For the remaining BDDCS class II drugs (Di, At, Ta, and Cl), all interactions are considered relevant, regardless of whether the affected targets are CYP enzymes, transporters, or other receptors. Furthermore, drugs are substrates of multiple targets, thus increasing the risk of serious interaction, with a multiplicity of plants/bioactives, which will be of more concern if the drug belongs to the HAM group. Among the nine drugs studied, Di and At meet a series of criteria, sufficient to be considered at risk for potential clinically significant interactions with the bioactives used in cognitive enhancement. Specifically, both are BDDCS class II drugs, belong to the HAM group, share targets with the 75 bioactives under study (only considering the bioactives modulating at least four different targets), and are substrates of 14 and 17 targets, respectively. Additionally, Di inhibits 13 targets and induces two, whereas At inhibits six targets and induces three (Figure 5). These drugs were therefore chosen for an in depth study, presented in the following sections.

#### 5.4.1. Diclofenac

Di is a nonsteroidal anti-inflammatory drug used to treat pain and inflammatory diseases. It acts by inhibiting COX1 and COX2. Di is mainly metabolized by several CYP enzymes: CYP1A1, CYP2B6, CYP2C8, CYP2C9, CYP2C18, CYP2C19, and CYP3A4. Di is a substrate of BSEP/ABCB11, BCRP, and OATP1B3 (Table 2). The official label of the drug indicates lethargy, drowsiness, nausea, vomiting, epigastric pain, and gastrointestinal bleeding, as symptoms of overdose.

**Figure 10:**
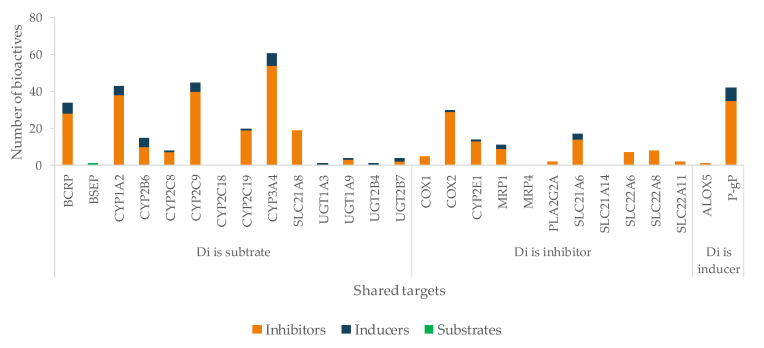
Number of bioactives affecting the shared targets with Di and respective mode of interaction (induction/inhibition). For clarity and due to relevance, bioactives were considered as substrates only for BSEP.

**Figure 11:**
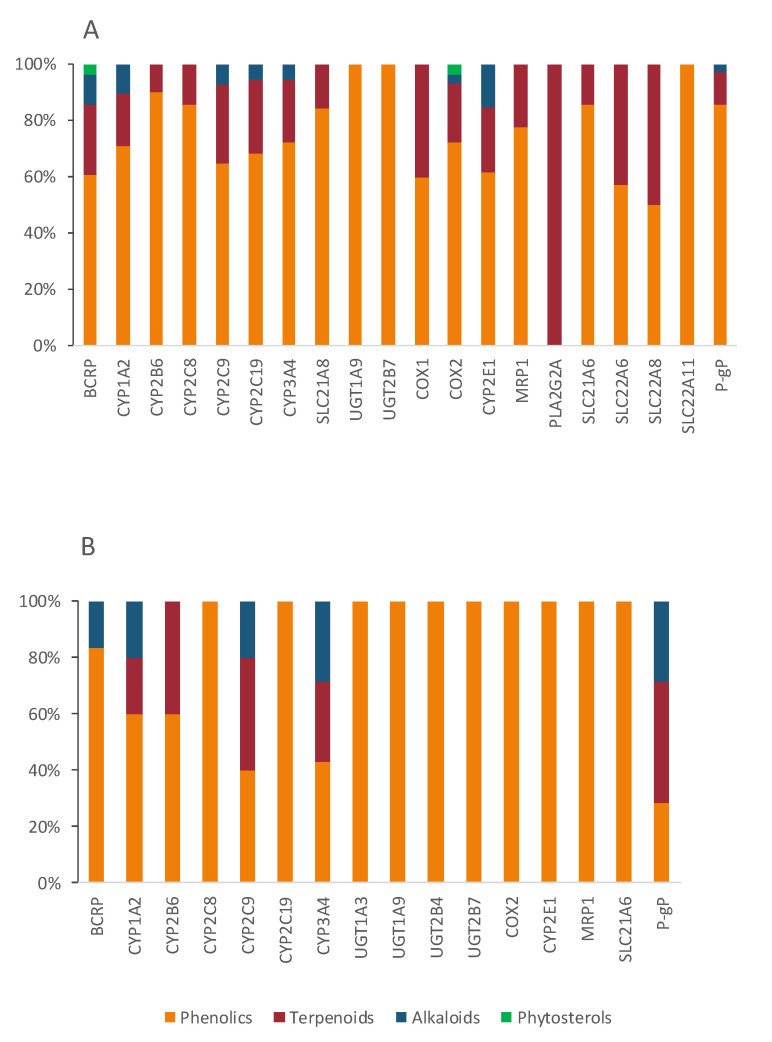
Characterization of the bioactives inhibiting (**A**) or inducing (**B**) Di shared targets, according to chemical class.

According to our results, Di is at great risk for HDI by modulation (mostly inhibition) of CYP enzymes, drug carriers (both efflux and uptake transporters), and COX enzymes (Figure 10). CYP1A2, CYP2C9, and CYP3A4 are the targets most susceptible to HDI, which can result in a decreased metabolization of the drug, and toxicity may occur. OATP1B3 (SLC21A8) is an uptake transporter exclusively expressed in the liver on the basolateral side of hepatocytes, responsible for the uptake of Di (Figure 7). The inhibition of this transporter results in less exposition of the drug to the metabolization site in the liver. OATP3 is inhibited by 19 bioactives, 16 of them being phenolic and three terpenes (Figure 11), such as EGCG, naringenin, quercetin, curcumin, hyperforin, apigenin, ursolic acid and p-kaempferol, among others. EGCG has been reported to inhibit diclofenac 4′-hydroxylation [618].

Moreover, naringenin and ursolic acid have the capacity to inhibit the six most affected Di targets (CYP1A2, CYP2C9, CYP2C19, CYP3A4, BCRP, and OATP1B3) and also COX2 synergistically with the mechanism of action of Di. In fact, a total of 48 (of the 170 bioactives) have the ability to inhibit COX2. All of these HDI may result in toxic levels of Di. Several authors have reported HDI involving Di [619,620]. For example, cinnamaldehyde (COX2 and CYP1A2 inhibitor) enhanced analgesia of a low dose of Di [206]. The authors hypothesized that cinnamaldehyde could increase Di absorption by increasing gastrointestinal blood flow by vasodilatation. Our results suggest that the inhibition of CYP1A2 and additional inhibition of COX2 could help explain those results. The same explanation could fit to the results of Matejczyk et al. [621], where the association of Di with chlorogenic acid resulted in increased toxicity to *E. coli* K-12. Resveratrol has shown to significantly interact with Di, probably due to CYP2C9 inhibition [622]. Our findings are in line with those conclusions, since resveratrol inhibits CYP2C9, CYP1A2, CYP2C19, CYP3A4, and OATP1B3 (SLC21A8), all contributing to the observed pharmacokinetic changes. A similar effect on CYP2C9 was found with association of Di with genistein [245]. Therefore, it is reasonable to conclude that bioactives with potential to inhibit CYP2C9 and COX2 (please refer to Figure 7 for additional examples) have a great chance to originate clinically relevant HDI and should not be associated with Di. 

BSEP is a uni-directional efflux transporter expressed in the liver, involved in the elimination of bile salts from the hepatocyte [623]. In the case of inhibition, bile salts will not be cleared and ultimately accumulate in the liver, causing cholestasis and liver injury [624]. Di and At are BSEP substrates, as well as glycyrrhizin. No reports were found on bioactives/plant inhibition of BSEP, but the fact that those three molecules may compete for the transporter must not be ignored and is highlighted in Figure 10 and Figure 11. Since no bioactive interfered with BSEP, neither by induction nor by inhibition, they were not represented; bioactives were considered as substrates only for BSEP of which Di is also a substrate and may indicate some sort of competition.

#### 5.4.2. Atorvastatin

At is a lipid lowering drug included in the statin group (lipophilic statin), considered the first-line treatment for dyslipidemia and in prevention of cardiovascular events. At can cause moderate side effects, but also, although rarely, serious side effects such as liver problems and kidney failure, as well as myopathy, which can progress to rhabdomyolysis, a potentially life-threatening complication. As reported in the drug label, the most common side effects include cold symptoms such as runny nose, sneezing, and coughing, diarrhea, heartburn, joint pain, forgetfulness, and confusion.

**Figure 12:**
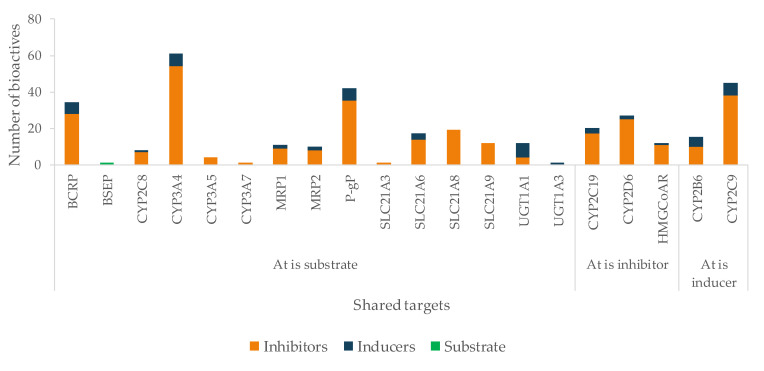
Number of bioactives affecting the shared targets with At and respective mode of interaction (induction/inhibition). For clarity and due to relevance, bioactives were considered as substrates only for BSEP.

The mechanism of action of At is due to competitive inhibition of the HMG-CoAR, the enzyme involved in the hepatic synthesis of cholesterol, through the production of mevalonate [625]. At is mainly metabolized by CYP3A4, 3A5, 3A7, and 2C8 enzymes, and it is also a substrate of BSEP, MRPs, P-gP, and BCRP, four SLC transporters and UGTs (Table 2). All of these targets contribute to At disposition, and, therefore, changes in any of them may modify At plasma profile and compromise the therapeutic outcomes. The great variety of CYP enzymes and efflux and uptake transporters of which At is a substrate, typical of a BDDCS class II drug, makes it more likely to suffer HDI, especially concerning since At is a HAM. In fact, amongst the bioactives under study, several have the potential to modulate most of those targets. For instance, there are 28 inhibitors of BCRP, 54 of CYP3A4, 35 of P-gP, 19 of SLC21A8, and 14 of SLC21A6 (Figure 12). Most bioactives have the potential to modulate multiple At targets, such as naringenin (inhibits BCRP, CYP3A4, HMGCoAR, P-gP, SLC21A6, SLC21A8, and SLC21A9 and induces UGT1A1 and UGT1A3), quercetin (inhibits CYP2C8, CYP3A4, MRP1, MRP2, P-gP, SLC21A6, SLC21A8, and SLC21A9 and induces UGT1A1 and BCRP), and EGCG (inhibits BCRP, CYP2C8, CYP3A4, CYP3A5, P-gP, SLC21A6, SLC21A8, and SLC21A9). Hence, if co-administered with At, they can cause pharmacokinetic and pharmacodynamic changes, with the possibility of compromising the efficacy and safety of the drug. Regarding HMGCoAR, naringenin, genistein, α-asarone, luteolin, resveratrol, rutin, naringin, chlorogenic acid, oleanolic acid, catalpol, β-sitosterol, and phytol may potentiate the inhibitory effect of At, since they are all inhibitors of the enzyme. Naringin proved to be a bioenhancer towards At, since the co-administration of both of them resulted in higher At plasma levels in rats. This effect was associated with the inhibition of CYP3A4 and P-gP by naringin [626].

The alkaloid berberine has been used as a cholesterol-lowering agent through a mechanism different from that of statins [627]. In the study of Feng et al. [628] the association of berberine with At had a greater inhibitory effect of CYP3A4 than the drug alone. Additionally, Glycyrrhizin and At are both substrates of BSEP, and they could compete for the binding site of BSEP, as discussed earlier for Di, which is a BSEP substrate as well.

**Figure 13:**
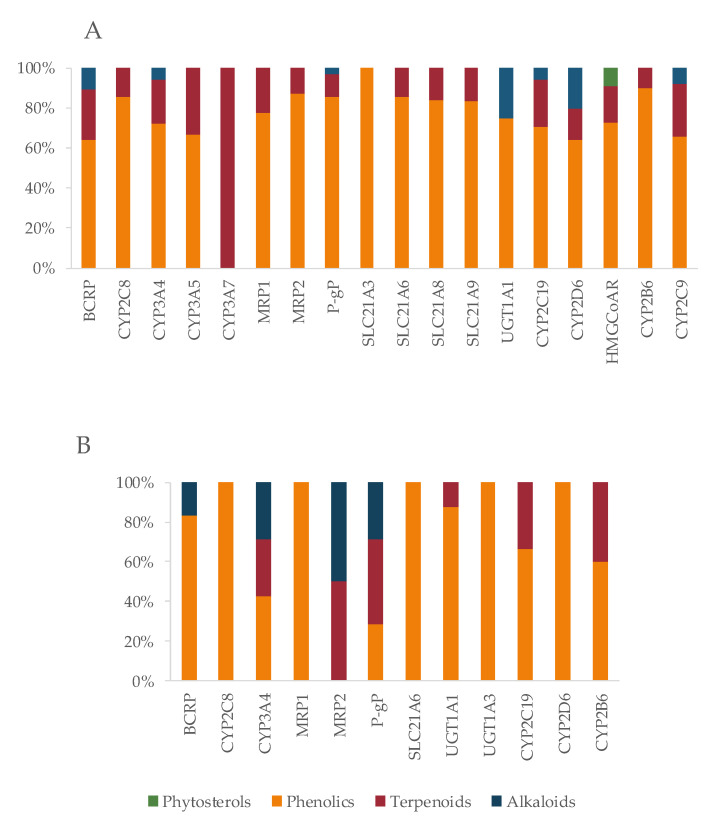
Characterization of the bioactives inhibiting (**A**) or inducing (**B**) At shared targets, according to chemical class.

OATP1B1 (SLC21A6), OATP1B3 (SLC21A8), and OATP2B1(SLC21A9) are the most relevant uptake transporters of At. OATP1B1 is inhibited by 14 bioactives (naringenin, EGCG, ECG, quercetin, genistein, curcumin, hyperforin, apigenin, ursolic acid, rutin, catechin, tannic acid, biochanin A, and myricetin); OATP1B3 is inhibited by 19 bioactives (EGCG, ECG, naringenin, quercetin, curcumin, hyperforin, apigenin, ursolic acid, nobiletin, p-kaempferol, rutin, naringin, biochanin A, gallic acid, quercetin-3-*O*-β-d-glucuronide, delphinidin, salvianolic acid, isorhamnetin, and glycyrrhizin) and OATP2B1 by 12 bioactives (EGCG, naringenin, naringin, quercetin, hypericin, hyperforin, apigenin, ursolic acid, nobiletin, p-kaempferol, quercetin-3-*O*-β-d-glucuronide, and cyanidin-3-*O*-β-glucoside). On all three carriers, more than 80% of the inhibitors are phenolic, and the remaining are terpenes (Figure 13A). Inducers are scarce (Figure 12) and mainly phenolic. Exceptionally, the MRP2 is not induced by any phenolic, but by one terpene (hyperforin) and one alkaloid (β-carboline) (Figure 13B). At and curcumin have been reported to act synergistically in lipid lowering effect [629].

Flavonoids like apigenin, quercetin, and kaempferol have been reported to competitively inhibit OATP1B1 [250]. This, in addition to CYP3A4 inhibition, could alter the pharmacokinetics and pharmacodynamics of At [253]. Hyperforin, a terpenoid, has been related to cause an increased efflux ratio of At in Caco-2 cells transcellular transport through the inhibition of OATP2B1 [443].

The number of HDI possibilities is overwhelming if we consider the huge number of plants reported to have an effect on cognition, the vast quantity of different bioactives present in each plant, and the complex network of targets involved in both drug and bioactive disposition and action. Moreover, drugs and bioactives can interact with the targets in different ways (substrates, inducers, inhibitors, and combinations of them, such as substrate and inhibitor of a given target). Nevertheless, not all HDI has a clinically significant impact on patients’ health. The data used to build up our HDI prediction tool has limitations (detailed in Section 6), such as the lack of definition of minimum amount of bioactive necessary to cause HDI, the fact that many data come from in vitro and/or animal studies, which do not always translate to humans, and the fact that no information is provided on the strength of the effect on targets [630]. On the other hand, bioactives studied in the present work are not usually taken alone, but rather included in food supplements (hence, briefly discussed in the following section), in association with other bioactives (for the same therapeutic goal or otherwise). The net effect of such a combination is not easy to predict, but it seems reasonable to speculate that the risk for HDI increases.

### 5.5. Food Supplements

Food supplements containing the phytochemicals identified in the plants were searched online to assess the prevalence of the potential interactions with drugs. Many of the plants/bioactives (about 80%) are indeed found in commercially available supplements, used for cognitive enhancement and identified in Table 3 with an asterisk. 

Products are advertised as brain tonics, nootropics, memory boosters, cognitive enhancers, memory protectors, memory enhancers, and cognition strengtheners, to support memory or improve brain attention, brain health, and cognitive function, concentration, and focus.

It is very common to encounter extracts of the plants rather than a specific bioactive. Additionally, mixtures derived from many different plants are the norm. As examples, NEUROTHERA™ [631] is a food supplement claiming to “Offer combined cognitive function benefits of 11 key neuronutrients” including extracts of *Withania somnifera* (root), *Vaccinium corymbosum* (Blueberry fruit concentrate), *Ginkgo biloba* (leaf), and *Eleutherococcus senticosus* (root); Maxgars Memory Booster [632] includes in the formula 14 different plants: *Bacopa monnieri*, *Centella asiatica*; *Withania somnifera*, *Terminalia arjuna*, *Terminalia bellirica*, *Phyllanthus emblica*, *Terminalia chebula*, *Convolvulus pluriens*, *Mucuna pruriens*, *Acorus calamus*, *Cyperus rotundus*, *Cassia occidentalis*, *Chlorophytum borivilianum*, and *Asparagus racemosus*.

The most common dosage forms are hard gelatin capsules, tablets, and soft gels, depending on the solubility of the bioactive/extract/tincture. Formulation and manufacturing processes (e.g., to avoid degradation, improve bioavailability, or correct organoleptic properties) are sometimes patent protected [633].

To safeguard consumers, who often confuse food supplements and drugs, the first should undergo a thorough, systematic monitorization, which would allow for the collection of reliable information on the safety of these products and thus the development of guidelines for its safe and effective use, as postulated for herbal medicines [634].

## 6. Final Remarks

Phytochemicals (bioactives) found in plants and food supplements are used as cognitive enhancers. Since they are part of the plant defense mechanism against predators, they can be toxic and interact with drugs sharing the same targets. The occurrence of the potential risks depends on exposure levels, which may be high, especially when various food supplements are taken simultaneously. To safely assess potential toxicity and HDI, the right daily dose of the bioactive should be established, standardized, and strictly controlled in food supplements.

The potential of such compounds to affect the pharmacokinetics or pharmacodynamics of drugs usually used by the elderly, a particularly sensitive age group due to polymedication, has not been studied yet. Thus, based on the documented reports available in literature for the interaction potential on targets, including enzymes, transporters, and receptors, an attempt has been made to postulate their HDI potential.

Limitations to the study relate to: (a) counting every reported action on target, regardless of whether they were obtained in vivo (animals or humans), in vitro, or in silico studies; (b) published studies are sometimes contradictory, in which case interaction was disregarded; (c) not every possible HDI mechanism has been contemplated (e.g., competitive binding to plasma proteins); and (d) the intensity of interactions is unknown, because they are dependent on dose and presence of other constituents in the plant matrix or food supplements. In fact, our study has considered the bioactives individually, and that might be misleading. Plants are sometimes used as a whole, or in the form of extracts, which contain very complex mixtures of compounds affecting the targets in diverse manners. Moreover, many food supplements contain cocktails of different plants, further hampering evaluation of the potential hazards.

Although efficacy evidence may be feeble (e.g., because studies were done in vitro, in animals, or in a limited number of individuals), the fact is that many of the plants studied are sold as food supplements and show potential for interacting with drugs, compromising the safety and efficacy of the latter. In fact, our study shows that HDI should not be ignored, strengthening the idea that when a patient starts a therapeutic regimen, or a new drug, these should be carefully assessed.

Plants containing alkaloids seem to be particularly involved in neurotransmission regulation, while polyphenols present the highest potential of neuroprotection and HDI (67% of the 75 bioactives affecting at least four targets are phenolic). Our research suggests that many herbs/bioactives interact with the drugs under study through a complex cytochrome P450 (mainly CYP3A4, CYP1A2, and CYP2C9) and/or transport mechanism, mainly involving P-glycoprotein, BCRP (ABCG2) and OATP1B3 carriers (SLC21A8). Approximately 30% of all the bioactives studied modulate CYP2C9 and CYP1A2, and the number rises to 45% when the target is CYP3A4 (70% of the drugs studied are substrates of this enzyme). In terms of transport, P-gP is the most involved carrier, modulated by 36% of the 170 bioactives studied, followed by BCRP (21%) and OATP1B3 (12%), in line with other reviews [630]. Additionally, we found COX2 to be a critical target for HDI (30%), which may be of particular concern for anti-inflammatory drugs such as Di. On the other hand, drugs may also affect the bioactive disposition. In our study, 89 of the 170 bioactives (52%) are substrates of at least one shared target with the 10 drugs.

Naturally, not every target modulation identified results in changes with clinical significance. Furthermore, a cellular transporter–enzyme interaction may not translate into an in vivo clinically relevant interaction. However, the higher the number of targets affected, the bigger the likelihood of interaction and of that interaction having clinical significance due to potentiation of effects.

Nevertheless, in this work we have created a database of interactions of 170 bioactives with 10 drugs, which may help doctors when prescribing, nutritionists in clinical practice, and pharmacists when counselling in the community or hospital pharmacy. Though only a limited number of drugs were selected, this work constitutes a helpful tool to anticipate interaction potential with other drugs affecting the same targets.

## Figures and Tables

**Table 1:** Characterization of the plants purported as acting as cognition enhancers and main mechanism(s) of action. Bioactive molecules were selected based on reported activity in cognition; other phytochemicals present in the plant were omitted.

Plant	Main Bioactive(S) for Cognition	Effect on Brain/Cognition	References
*Acorus calamus* ^+^^⬪^	α-Asarone β-Asarone	Neurotransmission	[55,56,57]
*Acorus gramineus*	α-Asarone β-Asarone	Neuroprotection and Neurotransmission	[58]
*Aframomum melegueta*	Gingerol Shogaol Quercetin p-Kaempferol	Neuroprotection and Neurotransmission	[59,60]
*Alpinia oxyphylla* ^■^^⬪^	Protocatechuic acid	Neuroprotection and Neurotransmission	[61]
*Anemarrhena asphodeloides* ^●^^■^^⬪^	Timosaponin AIII	Neuroprotection and Neurotransmission	[58]
*Angelica gigas* ^○^	Decursin Decursinol angelate	Neuroprotection and Neurotransmission	[58]
*Angelica sinensis* ^●▲^^⬪^	Z-ligustilide Coniferyl ferulate 11-Angeloylsenkyunolide F Ferulic acid	Neuroprotection and Neurotransmission	[18,62,63]
*Asparagus adscendens*	Shatavarin IV (Asparinin B) Conypododiol	Neuroprotection and Neurotransmission	[64,65]
*Asparagus racemosus* ^+^	Shatavarin IV (Asparinin B) Sarsasapogenin	Neuroprotection and Neurotransmission	[57,66]
*Atractylodes japonica* ^■^	Atractylenolide III	Neuroprotection	[67]
*Atractylodes lancea* ^●^^■^^⬪^	Atractylenolide III (Codonolactone) Stigmasterolβ-Sitosterol	Neurotransmission	[58]
*Avena sativa*	Avenanthramides A, B and C	Neuroprotection	[58]
*Bacopa monnieri* ^∗^^+^	Bacoside A and B	Neuroprotection and Neurotransmission	[51,68,69,70]
*Bauhinia rufescens*	d-pinitol (3-*O*-methyl-d-inositol) p-Coumaric acid Ferulic acid Hyperoside	Neuroprotection and Neurotransmission	[71]
*Buxus hyrcana*	Buxamine B	Neurotransmission	[22,72]
*Buxus papillosa*	Buxakashmiramine Cycloprotobuxine-C Cyclovirobuxeine-A Cyclomicrophylline-A *N,N*-dimethyl buxapapine	Neurotransmission	[22,72]
*Camellia sinensis* ^●^^∗^^⬪^	Caffein Epigallocatechin-3-gallate Epicatechin gallate Methylliberine	Neuroprotection and Neurotransmission	[55,57,73,74]
*Caragana chamiague* ^+^	(+)-α-Viniferin	Neurotransmission	[22]
*Centella asiatica* ^●^^∗▲^^+^^⬪^	Asiatic acid	Neuroprotection and Neurotransmission	[55,57,66]
*Cinnamomum wilsonii* (extract)	Cinnamaldehyde Eugenol	Neuroprotection and Neurotransmission	[75,76,77,78]
*Citrus aurantium* ^●^^■^^+^^⬪^	p-Synephrine	Neurotransmission	[79]
*Citrus reticulata* ^●^^■^^⬪^^○^	Nobiletin	Neuroprotection and Neurotransmission	[58]
*Clitoria ternatea* ^+^	Kaempferol	Neuroprotection and Neurotransmission	[57,66]
*Coffee arabica*	Caffein Methylliberine Theacrine	Neuroprotection and Neurotransmission	[55,74,80,81]
*Coleus forskohlii* ^+^	Forskolin (Colforsin)	Neuroprotection and Neurotransmission	[82]
*Convolvulus pluricaulis* ^+^	Kaempferol Kaempferol-3-glucoside Caffeic acid Convolamine Β-Sitosterol	Neuroprotection	[83]
*Coptis chinensis* ^●^^■▲^^⬪^	Berberine Coptisine Palmatine	Neurotransmission	[57]
*Coptis japonica* ^■▲^	Berberine	Neuroprotection	[57]
*Corydalis speciosa*	Palmatine	Neurotransmission	[22]
*Crocus sativus* ^●^^■▲^^+^^⬪^	Quercetin Crocins/picrocrocin Crocetin Safranal	Neuroprotection and Neurotransmission	[55,57,66]
*Croton tonkinensis*	Ent-kaurane	Neuroprotection and Neurotransmission	[16,84]
*Curcuma longa* ^●^^∗^^■▲^^+^^⬪^	Curcumin	Neuroprotection and Neurotransmission	[55,57]
*Cuscuta japonica*	Hyperoside Kaempferol	Neuroprotection and Neurotransmission	[85]
*Cynanchum atratum* ^⬪^	Cynatroside A Cynatroside B	Neurotransmission	[22]
*Cyperus rotundus* ^■^^+^^⬪^	Quercetin Kaempferol Catechin	Neuroprotection and Neurotransmission	[85]
*Dioscorea polystachya* ^■^^○^	Diosgenin	Neuroprotection	[58]
*Dioscorea oppositifolia* ^●^^■^^⬪^	Diosgenin	Neuroprotection	[86,87]
*Echium amoenum*	Cyanidin-3-glucoside	Neuroprotection	[88,89]
*Eleutherococcus senticosus*^●^^∗^^■▲^ (Siberian ginseng)	Syringin (Eleutheroside B)	Neuroprotection and Neurotransmission	[90,91,92,93]
*Eucharis grandiflora*	Sanguinine (*O*-Desmethylgalantamine)	Neuroprotection and Neurotransmission	[22]
*Foeniculum vulgare* ^●^^∗^^■▲^^+^^⬪^	Caffeic acid Chlorogenic acid 1,8-Cineole	Neuroprotection and Neurotransmission	[52,94,95,96]
*Galanthus nivalis*	Galantamine	Neurotransmission	[22,57]
*Galanthus woronowii* *(or ikariae)*	Galantamine Lycorine	Neurotransmission	[20,22,55]
*Ginkgo biloba* ^●^^∗▲^^⬪^	Ginkgolide A Ginkgolide B Bilobalide Isorhamnetin Protocatechuic acid	Neuroprotection and Neurotransmission	[20,55,57,81,97]
*Glycyrrhiza glabra* ^●^^∗^^■▲^^+^^⬪^	Glycyrrhizin Glycyrrhetinic acid	Neuroprotection	[57]
*Glycyrrhiza uralensis* ^●^^■▲^^⬪^	Isoliquiritigenin	Neuroprotection	[58]
*Haloxylon recurvum*	Haloxysterols A, B, C, D	Neurotransmission	[22]
*Huperzia serrata*	Huperzine A	Neuroprotection and Neurotransmission	[8,20,55,57]
*Hypericum perforatum* ^●^^∗▲^^⬪^	Hypericin Hyperforin Biapigenin (I3,II8-biapigenin) Quercetin Chlorogenic acid Rutin Hyperoside Kaempferol	Neuroprotection and Neurotransmission	[98,99,100,101,102]
*Ilex paraguariensis* ^●^	Chlorogenic acid Caffein Theobromine Quercetin Kaempferol	Neurotransmission	[22,57]
*Lepidium meyenii*^○^ (Maca Root Extract)	Quercetin β-Carbolines *N*-Benzylhexadecanamide *N*-Acetylbenzylamine *N*-3-Methoxybenzyl-linoleamide	Neuroprotection and Neurotransmission	[57,103,104,105,106,107]
*Lespedeza bicolor*	Catechin Rutin Daidzein Luteolin Naringenin Genistein	Neuroprotection and Neurotransmission	[22]
*Lycopodium clavatum*	α-Onocerin	Neurotransmission	[22]
*Lycoris radiata*	Galantamine	Neurotransmission	[22,57]
*Mangifera indica* ^+^^○^	Mangiferin	Neuroprotection and Neurotransmission	[108]
*Matricaria chamomilla* ^●^^∗▲^	Chlorogenic acid Caffeic acid Catechin Rutin Luteolin Apigenin	Neuroprotection and Neurotransmission	[22]
*Mauritia flexuosa*	Rutin	Neuroprotection and Neurotransmission	[22,109]
*Melissa officinalis* ^●▲^	Luteolin Apigenin Rosmarinic acid Protocatechuic acid	Neuroprotection and Neurotransmission	[20,55,57]
*Mentha spicata*	Rosmarinic acid Salvianolic acid	Neuroprotection and Neurotransmission	[110,111]
*Morinda lucida*	Phytol Oleanolic acid Chlorogenic acid p-Coumaric acid Daidzein Rutin Naringin Quercetin Naringenin Genistein	Neuroprotection and Neurotransmission	[112,113,114,115,116,117,118,119]
*Moringa peregrina*	Rutin Myricetin β-Sitosterol	Neuroprotection and Neurotransmission	[22]
*Mucuna pruriens* ^+^^○^	Gallic acid Genistein Levodopa (l-Dopa) Mucunadine Mucunine Prurienidine Prurienine β-Carbolines (Harmine) β-Sitosterol	Neuroprotection and Neurotransmission	[12,17,22,120]
*Narcissus assoanus*	Assoanine	Neurotransmission	[22]
*Narcissus confusus*	Galantamine Epinorgalantamine	Neurotransmission	[17,22,57]
*Narcissus poeticus*	11-Hydroxygalantamine	Neurotransmission	[22]
*Paeonia lactiflora* ^●^^■▲^^⬪^^○^	Paeonol	Neurotransmission	[22,58]
*Panax ginseng* ^●^^∗^^■▲^^⬪^^○^	Ginsenosides Rb, Rc, Rd, Rg1	Neuroprotection	[8,55,57,58,81]
*Pancratium illyricum*	11-α-hydroxy-*O*-methylleucotamine Galantamine Sanguinine Lycorine	Neuroprotection and Neurotransmission	[8,20,55,57,58,81,121]
*Paullinia cupana*^●^^○^ (Guarana)	Caffein	Neuroprotection and Neurotransmission	[81,122,123]
*Peganum harmala*	β-Carbolines (Harmine, 9-Methyl-9B-Carboline)	Neuroprotection and Neurotransmission	[124,125,126,127]
*Peltophorum pterocarpum*	Hyperoside Quercetin-3-*O*-β-d-glucuronide	Neuroprotection and Neurotransmission	[116]
*Phyllanthus emblica* ^+^^⬪^ *(Emblica officinalis)*	Ellagic acid Gallic acid Chebulagic acid Apigenin Quercetin Corilagin Luteolin Phyllanthin	Neuroprotection and Neurotransmission	[57,128,129,130]
*Piper nigrum* ^●^^∗^^+^^⬪^	Piperine	Neuroprotection	[131,132]
*Platycladus orientalis* ^⬪^	15-Methoxypinusolidic acid	Neurotransmission	[57,58,133]
*Polygala tenuifolia* ^●^^■^^⬪^	Tenuifoliside B Tenuifoliside C Tenuifolin Polygalaxanthone III	Neuroprotection and Neurotransmission	[134,135,136,137,138,139,140]
*Puerariae lobate* ^●^^∗^^■^^⬪^	Daidzein Puerarin	Neuroprotection	[141]
*Rehmannia glutinosa* ^■▲^^⬪^^○^	Catalpol	Neuroprotection	[58,142,143]
*Rhizoma acori* ^+^^⬪^	Eugenol β-Asarone	Neuroprotection	[144,145]
*Rhodiola rosea* ^∗^^○^	Rosavin Rosin Rosarin Salidroside	Neuroprotection and Neurotransmission	[146,147,148]
*Ribes nigrum*^●^ (Blackcurrant)	Myricetin Quercetin Isorhamnetin	Neuroprotection and Neurotransmission	[22,149]
*Rosmarinus officinalis* ^●^^∗▲^	Caffeic acid Chlorogenic acid Oleanolic acid Rosmarinic acid Ursolic acid α-Pinene Eucalyptol (Cineole) Luteolin Protocatechuic acid	Neuroprotection and Neurotransmission	[22]
*Salvia lavandulaefolia* ^●^	Eucalyptol α-Pinene	Neuroprotection and Neurotransmission	[20,22,55,57,150]
*Salvia miltiorrhiza* ^●^^∗^^■^^⬪^^○^	Tanshinone I Tanshinone IIA Tanshinone IIB Salvianolic acid	Neurotransmission	[24,151,152]
*Sarcococca saligna*	Sarsalignenone Vaganine Sarcocine Sarcodine Sarcorine Isosarcodine	Neurotransmission	[22]
*Saussurea costus* ^●^^■^	Apigenin Quercetin	Neurotransmission	[22,58]
*Schisandra chinensis* ^●^^■▲^^⬪^^○^	Schisandrin B (Gomisin N)	Neuroprotection and Neurotransmission	[57,58]
*Scrophularia buergeriana*	E-Harpagoside 8-*O*-E-p-methoxycinnamoylharpagide Harpagide	Neuroprotection and Neurotransmission	[153,154]
*Terminalia chebula* ^▲^^+^^⬪^^○^	Ellagic acid Tannic acid Chebulagic acid Corilagin Gallic acid 1,2,3,4,6-penta-*O*-galloyl-β-d-glucose	Neuroprotection and Neurotransmission	[22,57,66]
*Theobroma cacao* ^●^^∗^^■^	Theobromine Caffein Catechin	Neuroprotection and Neurotransmission	[155,156,157]
*Theobroma grandiflorum*	Theacrine	Neuroprotection	[74]
*Trifolium pretense*^∗▲^ (Red clover)	Biochanin A	Neuroprotection	[158]
*Trigonella foenum-graecum* ^●^^∗▲^^+^^⬪^^○^	Diosgenin	Neuroprotection and Neurotransmission	[159]
*Vaccinium myrtillus*^●^^∗▲^ (Bilberry)	Cyanidin-3-*O*-β-glucoside	Neuroprotection	[160]
*Vaccinium uliginosum L.* (Bog bilberry)	Quercetin Rutin (Quercetin-3-rutinoside) Malvidin-3-glucoside Cyanidin-3-*O*-β-glucoside Delphinidin	Neuroprotection and Neurotransmission	[22,160]
*Vaccinium angustifolium* (Lowbush Blueberry or Wild blueberry)	Malvidin 3-glucoside Malvidin 3-galactoside Caffeic acid Cyanidin-3-*O*-β-galactoside Cyanidin-3-*O*-β-glucoside Cyanidin-3-*O*-β-arabinose Malvidin-3-*O*-β-arabinose Peonidin-3-*O*-β-arabinose Delphinidin-3-*O*-β-galactoside	Neuroprotection and Neurotransmission	[161]
*Vinca minor*	Vinpocetine	Neuroprotection and Neurotransmission	[20,162]
*Vitex agnus-castus*^●^^∗▲^^+^^○^ (Chasteberry Extract)	Casticin	Neurotransmission	[163]
*Vitis vinifera* ^∗^^+^	Resveratrol Protocatechuic acid	Neuroprotection	[57,164]
*Withania somnifera* ^∗▲^^+^	Withaferin A Withanolide A, B Sitoindosides VII-X	Neuroprotection	[57,66,165,166,167,168,169]
*Zingiber officinale* Roscoe ^●^^∗^^■▲^^+^^⬪^	Gingerol Shogaol	Neuroprotection and Neurotransmission	[22,57,170,171,172,173]

Plant listed in Ph.Eur (^●^), USP (^∗^), JP (^■^), WHO monographs (^▲^), IP (^+^), PPRC (^⬪^), (HMC)-USP (^○^). **List of plants excluded:** *Achyranthes aspera*; *Albizia adianthifolia*; *Allium sativum*; *Asparagus cochinchinensis*; *Astragalus membranaceus*; *Beta vulgaris*; *Boerhavia diffusa*; *Cannabis sativa* L; *Cassia occidentalis*; *Celastrus paniculatus*; *Chlorophytum borivilianum*; *Commiphora whighttii*; *Dipsacus asper*; *Euonymus alatus*; *Evodia rutaecarpa*; *Evolvulus alsinoides*; *Gastrodia elata*; *Indigo naturalis*; *Juncus effusus*; *Lavandula angustifolia*; *Lawsonia inermis*; *Ligusticum officinale*; *Ligusticum wallichii*; *Liriope muscari*; *Lycium barbarum*; *Lycopersicon esculentum*; *Magnolia officinalis*; *Nardostachys jatamansi*; *Nicotiana tobaccum*; *Paeonia suffruticosa*; *Petiveria aliácea*; *Physostigma venenosum*; *Piper betel*; *Piper methysticum*; *Pueraria tuberosa*; *Punica granatum*; *Pycnanthus angolensis*; *Rheum* spp.; *Ricinus communis*; *Terminalia arjuna*; *Terminalia bellirica*; *Tinospora cordifolia*; *Tripterygium wilfordii*; *Uncaria tomentosa*; *Vaccinium alaskaense How*; *Vaccinium cespitosum Michx.*; *Vaccinium membranaceum* L.; *Vaccinium ovalifolium* Sm; *Valeriana officinalis*; *Xanthoceras sorbifolia*; *Zanthoxylum armatum*; *Ziziphus jujuba*.

**Table 2:** Characteristics of the drugs used in the study. The main enzymatic targets (substrates, inhibitors, and inducers) are specified.

Drug Name and Chemical Structure	HAM	ATC Class (Code)	BDDCS Class	ALOX5	AMPK	BSEP	COX	CYP	GLP-1	HMGCoAR	MAO	NMDA	ABC			PLA2G2A	SLC	UGT	References
													BCRP	MRP	Pgp		PEPT; OCT; OAT, MATE, OATP		
Alprazolam 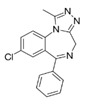	Y	Benzodiazepine derivatives (N05BA12)	I	^-^	^-^	^-^	^-^	3A4^S↓^ 3A5^S^ 3A7^S^ 2C9^S^ 2C19^S^	^-^	^-^	^-^	^-^	^-^	^-^	^-^	^-^	^-^	^-^	[217,218,219]
Atorvastatin 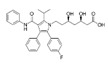	Y	HMG CoA reductase inhibitors (C10AA05)	II	^-^	^-^	^S^	^-^	3A4^S↑^ 3A5^S^ 3A7^S^ 2B6^↑^ 2C8^S↓^ 2C9^↑^ 2C19^↓^ 2D6^↓^	^-^	^↓^	^-^	^-^	^S^	1^S^ 2^S^ 4^S^ 5^S^	^S↓^	^-^	21A3^S^ 21A6^S↓^ 21A8^S^ 21A9^S^	1A1^S^ 1A3^S^	[218,219,220,221,222]
Clopidogrel 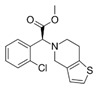	Y	Platelet aggregation inhibitors excl. heparin (B01AC04)	II	^-^	^-^	^-^	^-^	1A2^S^ 3A4^S^ 3A5^S^ 2B6^S↓^ 2C8^↓^ 2C9^S↓^ 2C19^S^	^-^	^-^	^-^	^-^	^↓^	^-^	^S^	^-^	21A8^S^ 22A1^↓^22A2^↓^	^-^	[218,219,223,224,225]
Diclofenac 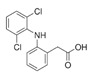	Y	Anti-inflammatory agents, non-steroids (S01BC03)	II	^↑^	^-^	^S^	1^↓^ 2^↓^	1A2^S^ 3A4^S↓^ 2B6^S^ 2C8^S^ 2C9^S↓^ 2C18^S^ 2C19^S^ 2E1^↓^	^-^	^-^	^-^	^-^	^S^	1^↓^ 4^↓^	^↑^	^↓^	21A6^↓^ 21A8^S^ 21A14^↓^ 22A11^↓^ 22A6^↓^ 22A8^↓^	1A3^s^ 1A9^s^ 2B4^s^ 2B7^s^	[218,219,226,227]
Memantine 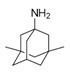	N	Other anti-dementia drugs (N06DX01)	III	^-^	^-^	^-^	^-^	2B6^↓^ 2C19^↓^	^-^	^-^	^-^	^ANT^	^-^	^-^	^-^	^-^	15A1^↓^ 15A2^↓^ 22A8^↓^ 22A1^↓^ 22A2^S↓^ 22A4^S^ 47A1^S^	^-^	[218,222,228,229,230,231]
Metformin 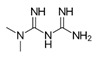	Y	Biguanides (A10BA02)	III		^↑^	^-^	^-^	^-^	^↑^	^-^	^-^	^-^	^S^	^-^	^-^	^-^	22A1^S^ 22A2^S↓^ 22A3^S^ 47A1^S^ 47A2^S^	^-^	[218,222,232,233,234,235]
Piracetam 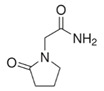	N	Other psychostimulants and nootropics (N06BX03)	III	^-^	^-^	^-^	^-^	^-^	^-^	^-^	^-^	^-^	^-^	^-^	^0^	^-^	^-^	^-^	[236]
Propranolol 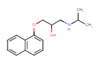	Y	β-blocking agents, non-selective (C07AA05)	I	^-^	^-^	^-^	^-^	1A1^↓^ 1A2^S^ 3A4^S^ 3A5^S^ 3A7^S^ 2C19^S^ 2D6^S↓^	^-^	^-^	A^↓^	^-^	^-^	^-^	^S^	^-^	22A2^↓^	^-^	[218,237,238,239,240]
Sertraline 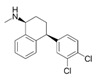	N	Selective serotonin reuptake inhibitors (N06AB06)	I	^-^	^-^	^-^	^-^	1A2^↓^ 3A4^S^ 2B6^S↓^ 2C9^S↓^ 2C19^S↓^ 2D6^S↓^ 2E1^S^	^-^	^-^	A^S^ B^S^	^-^	^S^	^-^	^S↓^	^-^	6A2^↓^ 6A3^↓S^ 6A4^↓S^ 36A1^↓^	1A3^S^ 1A6^S^ 2B4^S^ 2B7^S^	[217,218,219,241,242,243]
Tadalafil 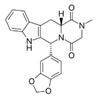	N	Drugs used in erectile dysfunction (G04BE08)	II	^-^	^-^	^-^	^-^	3A4^S^ 3A5^S^	^-^	^-^	^-^	^-^	^-^	^-^	^S^	^-^	^-^	^-^	[218,219,244]

^↓^—target inhibition; ^↑^—target induction; ^s^—drug is substrate of the target; ^ANT^—drug is a target antagonist; ^0^—No effect; (^—^)—Not reported; ABC—ATP-binding cassette; ALOX5—Arachidonate 5-lipoxygenase; AMPK—AMP-activated protein kinase; BCRP—Breast cancer resistance protein; BSEP—Bile salt export pump (ABCB11); COX—Cyclooxygenase; CYP—Cytochrome P450; GLP-1—Glucagon-like peptide-1; HMGCoAR—3-hydroxy-3-methyl-glutaril-CoA reductase; MAO—Monoamine Oxidase; MATE—Multi-antimicrobial extrusion protein; MRP—Multidrug resistance-associated protein; NMDA—*N*-methyl-d-aspartate; OAT—Organic anion transporter; OATP—Organic-anion-transporting polypeptide; OCT—Organic cation transport; P-gP—Glycoprotein P; UGT—Uridine diphosphate-glucuronosyltransferase; PLA2G2A—Phospholipase A2 Group IIA; SLC—Solute Carriers (15A/PEPT; 22A1-3/OCT1-3; 22A4/OCTN1; 22A6,8,11/OAT1,3,4; 47A/MATE; 21A3/OATP1A2; 21A6,8/OATP1B1,3; 21A9/OATP2B1; 21A14/OATP1C1); PEPT—Peptide transporter. HAM—High-Alert Medications [44]; Y—yes; N—No; ATC—Anatomical Therapeutic Chemical [36]; BDDCS—Biopharmaceutics Drug Disposition Classification System [37,38,39,40,41,42,43].

**Table 3:** Characterization of the identified phytochemicals in terms of origin and main enzymatic targets (substrates, inhibitors, and inducers).

Bioactive	Plant Sources	ALOX5	AMPK	BSEP	COX	CYP	GLP-1	HMGCoAR	MAO	NMDA	ABC			PLA2G2A	SLC	UGT	Reference
											BCRP	MRP	PgP		PEPT; OCT; OAT, MATE, OATP		
11-Angeloylsenkyunolide F *	*A. sinensis*	^—^	^—^	^—^	^—^	3A4^↑^2D6^↑^	^—^	^—^	^—^	^—^	^—^	^—^	^—^	^—^	^—^	^—^	[246]
Apigenin *	*M. chamomilla* *M. officinalis* *P. emblica* *S. costus*	^—^	^—^	^—^	^—^	1A2^↓^ 2C9^↓^ 2C19^↓^ 3A4^↓^	^—^	^—^	^—^	^—^	^↓^	^—^	^↓^	^—^	21A6^↓^ 21A8^↓^ 21A9^↓^ 22A8^↓^ 22A11^↓^ 22A4^↓^	1A1^↑^	[200,247,248,249,250,251,252,253]
α-Asarone	*A. calamus* *A. gramineus*	^—^	^—^	^—^	2^↓^	1A1^↓^ 3A4^↓^ 2B6^↓^ 2C8^↓^ 2C9^↓^ 2C19^↓^ 2D6^↓^ 2E1^↓^	^—^	^↓^	A^↓^ B^↓^	^Ant^	^—^	^—^	^↓^	^—^	^—^	^—^	[254,255,256,257,258,259,260]
β-Asarone	*A. calamus* *A. gramineus* *R. acori*	^—^	^↑^	^—^	2^↓^	3A4^↓^	^—^	^—^	A^↓^ B^↓^	^—^	^—^	^—^	^↓^	^—^	^—^	^—^	[255,259,261]
Asiatic acid *	*C. asiatica*	^—^	^—^	^—^	^—^	3A4^↓^ 2D6^↓^ 2C9^↓^	^—^	^—^	^—^	^—^	^—^	^—^	^S^	^—^	^—^	^—^	[262,263,264,265]
Assoanine	*N. assoanus*	^—^	^—^	^—^	^—^	^—^	^—^	^—^	^—^	^—^	^—^	^—^	^—^	^—^	^—^	^—^	—
Atractylenolide III *	*A. japonica* *A. lancea*	^—^	^—^	^—^	^—^	^—^	^—^	^—^	^—^	^—^	^—^	^—^	^—^	^—^	^—^	2B7^↓^	[266]
Avenanthramide A *	*A. sativa*	^—^	^—^	^—^	2^↓^	^—^	^—^	^—^	^—^	^—^	^—^	^—^	^↓^	^—^	^—^	^—^	[267,268]
Avenanthramide B *	*A. sativa*	^—^	^—^	^—^	2^↓^	^—^	^—^	^—^	^—^	^—^	^—^	^—^	^↓^	^—^	^—^	^—^	[268]
Avenanthramide C *	*A. sativa*	^—^	^↑^	^—^	2^↓^	^—^	^—^	^—^	^—^	^—^	^—^	^—^	^↓^	^—^	^—^	^—^	[268,269]
Bacoside A * and B *	*B. monnieri*	^—^	^—^	^—^	^—^	1A2^↓^ 3A4^↓^ 2C9^↓^ 2C19^↓^	^—^	^—^	^—^	^—^	^—^	^—^	^—^	^—^	^—^	^—^	[270]
*N*-Acetyl Benzylamine *	*L. meyenii*	^—^	^—^	^—^	^—^	^—^	^—^	^—^	^—^	^—^	^—^	^—^	^—^	^—^	^—^	^—^	—
*N*-Benzylhexadecanamide *	*L. meyenii*	^—^	^—^	^—^	^—^	^—^	^—^	^—^	^—^	^—^	^—^	^—^	^—^	^—^	^—^	^—^	—
Berberine *	*C. chinensis* *C. japonica*	^—^	^—^	^—^	^—^	1A2^↓^ 3A4^S↓^ 2C9^↓^ 2D6^↓^	^—^	^—^	^—^	^—^	^↓^	^—^	^↓↑S^	^—^	21A8^S^	^—^	[271,272,273,274,275,276,277]
Biapigenin *	*H. perforatum*	^—^	^—^	^—^	^—^	1A2^↓^ 3A4^↓^ 2C9^↓^ 2D6^↓^	^—^	^—^	A^↓^	^Ant^	^—^	^—^	^S^	^—^	^—^	^—^	[124,200,278,279]
Bilobalide *	*G. biloba*	^—^	^—^	^—^	^—^	1A1^↑^ 1A2^↑^ 3A4^↑^ 2B6^↑^ 2C9^↑^ 2E1^↑^	^—^	^—^	^—^	^↓^	^—^	^—^	^—^	^↓^	6A2^↓^ 22A6^↓^ 22A8^↓^	^—^	[218,280,281,282]
Biochanin A *	*T. pratense*	^—^	^—^	^—^	2^↓^	3A4^↓^	^—^	^—^	B^↓^	^↓^	^↓S^	^—^	^↓^	^—^	21A6^↓^ 21A8^↓^	^—^	[245,248,283,284,285,286,287]
Buxakashmiramine	*B. papillosa*	^—^	^—^	^—^	^—^	^—^	^—^	^—^	^—^	^—^	^—^	^—^	^—^	^—^	^—^	^—^	—
Buxamine B	*B. hyrcana*	^—^	^—^	^—^	^—^	^—^	^—^	^—^	^—^	^—^	^—^	^—^	^—^	^—^	^—^	^—^	—
Caffeic acid *	*C. pluricaulis* *F. vulgare* *M. chamomilla* *M. officinalis* *R. officinalis* *V. angustifolium*	^—^	^—^	^—^	1,2^↓^	1A1^↓^ 3A4^↓^ 2C9^↓^ 2C19^↓^ 2D6^↓^	^—^	^—^	^—^	^—^	^↑^	^—^	^—^	^↓^	^—^	^—^	[288,289,290,291]
Caffein *	*C. arabica* *I. paraguariensis* *P. cupana* *T. cacao* *C. sinensis*	^—^	^—^	^—^	^—^	1A2^↓^ 3A4^↓^	^—^	^—^	^—^	^—^	^↓^	^—^	^—^	^—^	^—^	^—^	[292,293]
β-Carbolines (Harmine) *	*L. meyenii* *M. pruriens* *P. harmala*	^—^	^—^	^—^	^—^	1A1^↓^ 1A2^↓^ 2C9^↓^ 2C19^↓^ 2D6^↓^ 2E1^↓^	^—^	^—^	A^↓^ B^↓^	^—^	^↓^	2^↑S^	^—^	^—^	^—^	^—^	[124,126,294,295,296]
9-Methyl-9B-Carboline *	*P. harmala*	^—^	^—^	^—^	^—^	3A4^↓^ 2D6^S↓^	^—^	^—^	A^↓^ B ^↓^	^—^	^—^	^—^	^0^	^—^	^—^	^—^	[127,296,297]
Casticin *	*V. agnus-castus*	^—^	^—^	^—^	^—^	3A4^↓^ 2C9^↓^	^—^	^—^	^—^	^—^	^—^	^—^	^—^	^—^	^—^	^—^	[298]
Catalpol *	*R. glutinosa*	^—^	^—^	^—^	^—^	3A4^↑↓^ 2C9^↑↓^ 2E1^↓^	^—^	^↓^	^—^	^—^	^—^	^—^	^↑^	^—^	^—^	^—^	[292,299,300,301]
Catechin *	*C. rotundus* *L. bicolor* *M. chamomilla* *T. cacao*	^—^	^—^	^—^	^—^	1A2 ^↓^ 3A4 ^↓^ 2C9 ^↓^	^—^	^—^	^—^	^—^	^—^	^—^	^↓^	^—^	47A1^↓^ 47A2^↓^ 22A1^↓^ 22A2^↓^ 21A3^↓^ 21A6^↓^	^—^	[302,303]
Chebulagic acid *	*T. chebula* *P. emblica*	^—^	^—^	^—^	2^↓^	^—^	^—^	^—^	^—^	^—^	^—^	^—^	^↓^	^—^	^—^	^—^	[304,305]
Chlorogenic acid *	*F. vulgare* *H. perforatum* *I. paraguariensis* *M. chamomilla* *M. lucida* *R. officinalis*	^—^	^↑^	^—^	2^↓^	1A2^↓^ 3A4^o^ 2E1^↓^	^—^	^↓^	B^↓^	^—^	^—^	^—^	^—^	^↓^	22A6^↓^ 22A8^↓^	^—^	[306,307,308,309,310,311]
1,8-Cineole (eucalyptol) *	*F. vulgare* *S. lavandulaefolia* *R. officinalis*	^—^	^—^	^—^	^—^	3A4^S^	^—^	^—^	^—^	^—^	^—^	^—^	^S^	^—^	^—^	^—^	[312]
Cinnamaldehyde *	*C. wilsonii*	^—^	^↑^	^—^	2^↓^	1A2^↓^ 2E1^↓^	^—^	^—^	B^S^	^Ant^	^—^	^—^	^↓^	^—^	^—^	^—^	[75,313,314,315,316,317]
Coniferyl ferulate *	*A. sinensis*	^—^	^—^	^—^	^—^	3A4^↑^ 2D6^↑^	^—^	^—^	^—^	^—^	^—^	^—^	^↓^	^—^	^—^	^—^	[246,318]
Convolamine *	*C. pluricaulis*	^—^	^—^	^—^	^—^	^—^	^—^	^—^	^—^	^—^	^—^	^—^	^—^	^—^	^—^	^—^	—
Conypododiol	*A. adscendens*	^—^	^—^	^—^	^—^	^—^	^—^	^—^	^—^	^—^	^—^	^—^	^—^	^—^	^—^	^—^	—
Coptisine	*C. chinensis*	^—^	^—^	^—^	^—^	1A2^↓^ 3A4^↑^ 2C9^↑^ 2D6^↓^	^—^	^—^	A^↓^ B^↓^	^—^	^—^	^—^	^S↑^	^—^	22A1^S↓^ 22A2^S↓^	^—^	[272,273,274,319,320]
p-Coumaric acid *	*B. rufescens* *M. lucida*	^—^	^↑^	^—^	2^↓^	^—^	^—^	^—^	^—^	^—^	^—^	^—^	^—^	^—^	^—^	^—^	[321,322]
Corilagin *	*T. chebula* *P. emblica*	^—^	^—^	^—^	^—^	^—^	^—^	^—^	^—^	^—^	^—^	2^↑^	^—^	^—^	^—^	2B4^↑^	[323]
Crocetin *	*C. sativus*	^—^	^—^	^—^	^—^	^—^	^—^	^—^	^—^	^Ant^	^—^	1^↓^ 2^↓^	^S^	^—^	^—^	^—^	[324,325,326]
Crocin *	*C. sativus*	^—^	^—^	^—^	1^↓^ 2^↓^	3A4^↓^ 3A5^↓^ 3A7^↓^ 2B6^↓^	^—^	^—^	A^↓^ B^↓^	^—^	^—^	1^↓^ 2^↓^	^↓^	^—^	^—^	^—^	[327,328,329]
Curcumin *	*C. longa*	^—^	^—^	^—^	^—^	1A2^↓^ 3A4^↓^ 2B6^↓^ 2C9^↓^ 2D6^↓^	^—^	^—^	^—^	^—^	^↑↓^	1^↓^ 2^↓^	^↓^	^—^	21A6^↓^ 21A8^↓^	1A1^↓^ 1A6^↓^ 1A9^↓^	[247,330,331,332,333,334,335,336,337,338,339]
Cyanidin-3-*O*-β-arabinose *	*V. angustifolium*	^—^	^—^	^—^	^—^	1A2^↓^ 3A4^↓^	^—^	^—^	^—^	^—^	^—^	^—^	^—^	^—^	^—^	^—^	[340]
Cyanidin-3-*O*-β-galactoside *	*V. angustifolium*	^—^	^—^	^—^	^—^	1A2^↓^ 3A4^↓^ 2C9^↓^	^—^	^—^	^—^	^—^	^S^	^—^	^0^	^—^	^—^	^—^	[341,342]
Cyanidin-3-*O*-β-glucoside *	*E. amoenum* *V. angustifolium* *V. uliginosum L.*	^—^	^—^	^—^	2^↑^	3A4^↓^ 2C9↓	^—^	^—^	^—^	^—^	^S^	^—^	^—^	^↓^	21A9^↓^	^—^	[341,343,344,345]
Cyclomicrophylline A	*B. papillosa*	^—^	^—^	^—^	^—^	^—^	^—^	^—^	^—^	^—^	^—^	^—^	^—^	^—^	^—^	^—^	—
Cycloprotobuxine-C	*B. papillosa*	^—^	^—^	^—^	^—^	^—^	^—^	^—^	^—^	^—^	^—^	^—^	^—^	^—^	^—^	^—^	—
Cyclovirobuxeine-A	*B. papillosa*	^—^	^—^	^—^	^—^	^—^	^—^	^—^	^—^	^—^	^—^	^—^	^—^	^—^	^—^	^—^	—
Cynatroside A	*C. atratum*	^—^	^—^	^—^	^—^	^—^	^—^	^—^	^—^	^—^	^—^	^—^	^—^	^—^	^—^	^—^	
Cynatroside B	*C. atratum*	^—^	^—^	^—^	^—^	^—^	^—^	^—^	^—^	^—^	^—^	^—^	^—^	^—^	^—^	^—^	—
Daidzein *	*L. bicolor* *M. lucida* *P. lobata*	^—^	^—^	^—^	^—^	1A1^S^ 1A2^S^ 3A4^↓^	^—^	^—^	^—^	^—^	^↓S^	1^↑^	^↑^	^—^	^—^	1A1^S^ 1A9^S^	[245,285,346,347,348,349]
Decursin *	*A. gigas*	^—^	^—^	^—^	^—^	1A1^↓^ 1A2^↓^	^—^	^—^	^—^	^—^	^—^	2^↓^	^↓^	^—^	^—^	^—^	[247,350]
Decursinol angelate *	*A. gigas*	^—^	^—^	^—^	^—^	1A1^↓^ 1A2^↓^ 3A4^S^ 2C19^S^	^—^	^—^	^—^	^—^	^—^	^—^	^S^	^—^	^—^	^—^	[350,351,352]
Delphinidin *	*V. uliginosum L.*	^—^	^—^	^—^	^—^	3A4^↓^ 2B6^↓^ 2C9^↓^	^—^	^—^	^—^	^—^	^↓^	^—^	^—^	^—^	21A8^↓^	^—^	[341,344,353]
Delphinidin-3-*O*-β-galactoside *	*V. angustifolium*	^—^	^—^	^—^	^—^	^—^	^—^	^—^	^—^	^—^	^—^	^—^	^—^	^—^	^—^	^—^	—
Diosgenin *	*D. polystachya* *D. oppositifolia* *T. foenum-graecum*	^—^	^—^	^—^	2^↑^	3A4^↓^	^—^	^—^	^—^	^—^	^—^	^—^	^S^	^—^	^—^	^—^	[354,355,356]
Ellagic acid *	*T. chebula* *P. emblica*	^—^	^—^	^—^	^—^	1A1^↑↓^ 2B6^↓^ 2E1^↓^	^—^	^—^	^—^	^—^	^↓^	^—^	^S^	^—^	22A6^↓^ 22A11^↓^	^—^	[357,358,359,360,361,362]
Ent-kaurane *	*C. tonkinensis*	^—^	^↑^	^—^	2^↓^	^—^	^—^	^—^	^—^	^—^	^—^	^—^	^↓^	^—^	^—^	^—^	[363,364,365]
Epicatechin gallate (ECG) *	*C. sinensis*	^—^	^—^	^—^	^—^	1A1^↓^ 1A2^↓^ 3A4^↓^	^—^	^—^	^—^	^—^	^—^	^—^	^↓^	^—^	21A6^S↓^ 21A8^↓^	^—^	[366,367,368,369]
Epigallocatechin-3-gallate (EGCG) *	*C. sinensis*	^—^	^—^	^—^	^—^	1A1^↓^ 1A2^↓^ 3A4^↓^ 3A5^↓^ 2B6^↓^ 2C8^↓^ 2C9^↓^ 2C19^↓^ 2D6^↓^ 2E1^↓^	^—^	^—^	^—^	^—^	^↓^	^—^	^S↓^	^—^	21A6^↓^ 21A8^S↓^ 21A9^↓^ 22A1^↓^ 22A2^↓^ 47A1^↓^ 47A2^↓^	^—^	[248,302,303,345,367,370,371,372,373,374,375,376]
Epinorgalantamine	*N. confusus* *G. woronowii*	^—^	^—^	^—^	^—^	^—^	^—^	^—^	^—^	^—^	^—^	^—^	^—^	^—^	^—^	^—^	—
Eugenol *	*C. wilsonii* *R. acori* *R. officinalis*	^—^	^↓^	^—^	2^↓^	1A1^↓^ 2E1^S^	^—^	^0^	A^↓^	^↓^	^—^	^—^	^0↓^	^—^	^—^	1A1^↑^	[77,247,377,378,379,380,381,382,383]
Ferulic acid *	*A. sinensis* *B. rufescens*	^—^	^—^	^—^	^—^	1A1^↑^ 1A2^↑S^ 3A4^↑S^ 2B6^↑S^ 2C8^↑S^ 2C9^↑S^ 2C19^↑S^ 2D6^↑S^	^—^	^—^	B^↓^	^Ant^	^—^	^—^	^↓^	^—^	^—^	1A1^S^ 2B7^S^	[246,384,385,386,387]
Forskolin *	*C. forskohlii*	^—^	^↓^	^—^	2^↓^	3A4^S↑^ 2C^↑^ 2B6^↑^	^↑^	^—^	^—^	^↑^	^—^	^—^	^s↓^	^—^	^—^	^—^	[388,389,390,391,392,393,394,395,396,397]
Galantamine *	*G. nivalis* *G. woronowii* *L. radiata* *N. confusus* *P. illyricum*	^—^	^—^	^—^	^—^	3A4^S 0^ 2D6^S^	^—^	^—^	^—^	^—^	^—^	^—^	^↓^	^—^	^—^	1A1^S^	[398,399,400,401,402]
11-Hydroxy Galantamine *	*N. poeticus*	^—^	^—^	^—^	^—^	^—^	^—^	^—^	^—^	^—^	^—^	^—^	^—^	^—^	^—^	^—^	—
Gallic acid *	*M. pruriens* *P. emblica* *T. chebula*	^—^	^—^	^—^	2^↓^	3A4^↓^ 2D6^↓^	^—^	^—^	A^↓^	^—^	^—^	^—^	^↓^	^—^	21A8^↓^	2B7^↑^	[403,404,405,406,407,408,409]
Genistein *	*L. bicolor* *M. lucida* *M. pruriens*	^—^	^↑^	^—^	2^↓^	1A1^↓^ 1A2^↓S^ 3A4^↑^ 2C8^↓^ 2C9^↓^ 2D6^↓^	^—^	^↓^	A^↓^ B^↓^	^↓^	^↓S^	1^↓^ 2^↓S^	^↓^	^—^	21A6^↓S^	^—^	[218,285,369,374,409,410,411,412,413,414,415]
Gingerol *	*A. melegueta* *Z. officinale Rosc.*	^—^	^—^	^—^	^—^	3A4^↓^ 2C9^↓^ 2C19^↓^	^—^	^—^	^—^	^—^	^—^	^—^	^↓^	^—^	^—^	^—^	[416,417]
Ginkgolide A *	*G. biloba*	^—^	^—^	^—^	2^↓^	3A4^↓^ 2C9^↓^	^—^	^—^	^—^	^↓^	^—^	^—^	^↑^	^↓^	6A2^↓^ 22A6^↓^ 22A8^↓^	^—^	[218,281,418,419,420]
Ginkgolide B *	*G. biloba*	^—^	^—^	^—^	^—^	3A4^↓^ 2C9^↓^	^—^	^—^	^—^	^—^	^—^	^—^	^↑^	^↓^	6A2^↓^ 22A6^↓^ 22A8^↓^	^—^	[218,281,418]
Ginsenoside Rb *	*P. ginseng*	^—^	^—^	^—^	^—^	1A1^↑^ 1A2^↓^ 3A4^↓^ 2C9^↓^	^—^	^—^	^—^	^—^	^↓^	^—^	^—^	^—^	21A8^S^	^—^	[421,422,423]
Ginsenoside Rc *	*P. ginseng*	^—^	^—^	^—^	^—^	3A4^↓^ 2C9 ^↓^	^—^	^—^	^—^	^—^	^—^	^—^	^—^	^—^	21A8^S^	^—^	[421,423]
Ginsenoside Rd *	*P. ginseng*	^—^	^—^	^—^	^—^	3A4^↓^ 2C9^↓^ 2C19^↓^ 2D6^↓^	^—^	^—^	^—^	^—^	^↓^	^—^	^—^	^—^	21A8^S^	^—^	[421,423]
Ginsenoside Rg *	*P. ginseng*	^—^	^—^	^—^	^—^	1A1^↑^ 1A2^↓^ 3A4^↓^ 2C9^↓^ 2D6^↓^	^—^	^—^	^—^	^—^	^S^	^—^	^—^	^—^	21A8^S^	^—^	[421,422,423]
1,2,3,4,6-Penta-*O*-galloyl-β-d-glucose *	*T. chebula*	^—^	^—^	^—^	2^↓^	^—^	^—^	^—^	^—^	^—^	^—^	^—^	^↓^	^—^	^—^	^—^	[424,425]
Glycyrrhetinic acid *	*G. glabra*	^—^	^—^	^—^	^—^	^—^	^—^	^—^	^—^	^—^	^—^	^—^	^—^	^—^	21A9^↓^	^—^	[426]
Glycyrrhizin *	*G. glabra*	^—^	^—^	^S^	^—^	3A4^↓^ 2D6^↓^	^—^	^—^	^—^	^—^	^—^	^—^	^↑↓^	^—^	21A8^↓^	^—^	[247,257,427,428,429]
Haloxysterol A *, B *, C *, D *	*H. recurvum*	^—^	^—^	^—^	2^↓^	^—^	^—^	^—^	^—^	^—^	^—^	^—^	^—^	^—^	^—^	^—^	[430]
Harpagide *	*S. buergeriana*	^—^	^—^	^—^	^—^	3A4^0^	^—^	^—^	^—^	^—^	^—^	^—^	^—^	^—^	^—^	^—^	[431]
8-*O*-E-p-Methoxy Cinnamoyl Harpagide	*S. buergeriana*	^—^	^—^	^—^	^—^	^—^	^—^	^—^	^—^	^—^	^—^	^—^	^—^	^—^	^—^	^—^	—
E-Harpagoside *	*S. buergeriana*	^—^	^—^	^—^	1^↓0^ 2^↓0^	3A4^0^	^—^	^—^	^—^	^—^	^—^	^—^	^↓^	^—^	^—^	^—^	[431,432,433,434]
Huperzine A *	*H. serrata*	^—^	^—^	^—^	^—^	1A2^↑^ 3A4^↑0^	^—^	^—^	^—^	^—^	^—^	^—^	^S^	^—^	^—^	^—^	[435,436,437]
Hyperforin *	*H. perforatum*	^↓^	^—^	^—^	1^↓^ 2^0^	2D6^↓0^ 2C9^↓^ 3A4^↓^ 3A5^↓^ 1A2^↓^ 2C19^↓^	^—^	^—^	A^0^	^Ant^	^↓^	2^↑^	^↑↓^	^—^	21A6^↓^ 21A8^↓^ 21A9^↓^	1A1^↑↑^	[98,124,202,278,438,439,440,441,442,443,444,445,446,447,448,449]
Hypericin *	*H. perforatum*	^—^	^↑^	^—^		2D6^↓0^ 2C9^↓^ 3A4^↓^ 1A2^↓^ 2C19^↓^	^—^	^—^	A^↓0^ B^↓0^	^Ant^	^↓^	1^↑^	^↑^	^—^	21A9^↓S^	1A6^↓^	[102,124,278,336,442,443,444,447,450,451,452,453,454,455]
Hyperoside *	*B. rufescens* *C. japonica* *H. perforatum* *P. pterocarpum*	^—^	^—^	^—^	^—^	2D6^↓^ 2C9^↓^	^—^	^—^	^—^	^—^	^—^	^—^	^S^	^—^	^—^	^—^	[289,456]
Isoliquiritigenin *	*G. uralensis*	^—^	^—^	^—^	^—^	1A2^↓^ 3A4^↓^ 2C9^↓^ 2C19^↓^	^—^	^—^	^—^	^—^	^—^	^—^	^—^	^—^	^—^	^—^	[457]
Isorhamnetin *	*G. biloba* *R. nigrum*	^—^	^—^	^—^	^—^	1A2^↓^ 3A4^↓^	^—^	^—^	^—^	^—^	^—^	2^↓^	^S↑↓^	^—^	21A8^↓^	^—^	[200,247,249,458,459]
Isosarcodine	*S. saligna*	^—^	^—^	^—^	2^↓^	^—^	^—^	^—^	^—^	^—^	^—^	^—^	^—^	^—^	^—^	^—^	[430]
p-Kaempferol *	*A. melegueta* *C. pluricaulis* *C. japonica* *C. rotundus* *H. perforatum* *I. paraguariensis*	^—^	^—^	^—^	^—^	1A2^↓^ 3A4^↓^ 2C9^↓^ 2C19^↓^	^—^	^—^	^—^	^Ant^	^—^	^—^	^↓S^	^—^	21A8^↓^ 21A9^↓^ 22A4^↓^ 22A8^↓^	1A1^↑^	[200,218,250,251,374,460,461,462,463]
Kaempferol-3-glucoside *	*C. ternatea* *C. pluricaulis*	^—^	^—^	^—^	^—^	^—^	^—^	^—^	^—^	^—^	^—^	^—^	^—^	^—^	21A6^↓^ 21A8^↓^	^—^	[345,463]
11α-Hydroxy-*O*-methylleucotamine	*P. illyricum*	^—^	^—^	^—^	^—^	^—^	^—^	^—^	^—^	^—^	^—^	^—^	^—^	^—^	^—^	^—^	—
*N*-3-methoxybenzyl-linoleamide (macamide) *	*L. meyenii*	^—^	^—^	^—^	^—^	^—^	^—^	^—^	^—^	^—^	^—^	^—^	^—^	^—^	^—^	^—^	—
Levodopa *	*M. pruriens*	^—^	^—^	^—^	^—^	^—^	^—^	^—^	^—^	^Ant^	^—^	^—^	^S^	^—^	^—^	^—^	[464,465]
Luteolin *	*L. bicolor* *M. chamomilla* *M. officinalis* *P. emblica* *R. officinalis*	^—^	^↑^	^—^	2^↓^	1A2^↓^ 3A4^↓^ 3A5^0^ 2B6^↓^ 2C8^↓^ 2C9^↓^ 2C19^↓^ 2D6^↓^ 2E1^↓^	^—^	^↓^	A^↓^ B^↓^	^—^	^↓^	^—^	^—^	^—^	^—^	1A1^↓S^ 1A9^↓^ 2B7^↓^	[276,466,467,468,469,470,471,472]
Lycorine	*G. woronowii* *P. illyricum*	^—^	^—^	^—^	^—^	3A4^0^	^—^	^—^	^—^	^—^	^—^	^—^	^0^	^—^	^—^	^—^	[342,473]
Malvidin 3-galactoside *	*V. angustifolium*	^—^	^—^	^—^	^—^	3A4^↓^ 2C9^↓^	^—^	^—^	^—^	^—^	^↓S^	^—^	^↓^	^—^	21A6^↑^	^—^	[341,353,474]
Malvidin 3-glucoside *	*V. angustifolium* *V. uliginosum L.*	^—^	^—^	^—^	^—^	3A4^↓^ 2C9^↓^	^—^	^—^	^—^	^—^	^↓^	^—^	^↓^	^—^	21A6^↑^	^—^	[341,353,474]
Malvidin-3-*O*-β-arabinose *	*V. angustifolium*	^—^	^↑^	^—^	^—^	^—^	^—^	^—^	^—^	-	^—^	^—^	^—^	^—^	^—^	^—^	[475]
Mangiferin *	*M. indica*	^—^	^—^	^—^	^—^	1A1^↓^ 1A2^↓^ 3A4^↓^ 2C8^↓^ 2B6^↓^ 2D6^↓^	^—^	^—^	^—^	^—^	^—^	^—^	^↓^	^—^	^—^	1A1^↓^ 1A9^↓^ 2B7^↓^	[198,476,477]
Methylliberine *	*C. arabica*	^—^	^—^	^—^	^—^	^—^	^—^	^—^	^—^	^—^	^—^	^—^	^—^	^—^	^—^	^—^	—
Mucunadine *	*M. pruriens*	^—^	^—^	^—^	^—^	^—^	^—^	^—^	^—^	^—^	^—^	^—^	^—^	^—^	^—^	^—^	—
Mucunine *	*M. pruriens*	^—^	^—^	^—^	^—^	^—^	^—^	^—^	^—^	^—^	^—^	^—^	^—^	^—^	^—^	^—^	—
Myricetin *	*M. peregrina* *R. nigrum*	^—^	^—^	^—^	^—^	1A2^↓^ 3A4^↓^ 2D6^↓^	^—^	^—^	^—^	^—^	^↓^	1^↓^	^↓S^	^—^	21A6^↓^	^—^	[218,247,248,276,478]
Naringenin	*L. bicolor* *M. lucida*	^—^	^↑^	^—^	2^↓^	1A2^↓0^ 3A4^↓^ 2B6^0^ 2C9^↓^ 2C19^↓^ 2D6^0^ 2E1^0^	^—^	^↓^	A^↓^ B^↓^	^—^	^↓^	^—^	^↓^	^—^	22A6^↓^ 21A6^↓^ 21A8^↓^ 21A9^↓^	1A1^↑^ 1A3^↑^ 1A6^↑^ 1A9^↑^ 2B4^↑^ 2B7^↑^	[200,218,247,253,479,480,481,482,483,484,485,486,487,488]
Naringin *	*M. lucida*	^—^	^↑^	^—^	2^↓^	3A4^↓↑^ 1A2^↓^	^—^	^↓^	A^↓^	^—^	^↑^	^—^	^↓^	^—^	21A9^↓^	^—^	[200,253,338,483,487,489,490,491,492,493]
*N,N*-dimethyl buxapapine	*B. papillosa*	^—^	^—^	^—^	^—^	^—^	^—^	^—^	^—^	^—^	^—^	^—^	^—^	^—^	^—^	^—^	—
Nobiletin *	*C. reticulata*	^—^	^—^	^—^	2^↓^	1A1^↓↑^ 1A2^↓↑^ 3A4^↓^	^—^	^—^	A^↓^ B^↓^	^—^	^↓^	1^↓^	^↓^	^—^	21A8^↓^ 21A9^↓^	^—^	[208,247,337,487,494,495,496,497]
Oleanolic acid *	*M. lucida* *R. officinalis*	^—^	^↑^	^—^	2^↑^	1A2^↓^ 3A4^↓^	^—^	^↓^	A^↓^	^—^-	^↓^	1^↓^	^0^	^—^	^—^	^—^	[276,498,499,500,501,502,503]
α-Onocerin *	*L. clavatum*	^—^	^—^	^—^	^—^	^—^	^—^	^—^	^—^	^—^	^—^	^—^	^—^	^—^	^—^	^—^	—
p-Synephrine *	*C. aurantium*	^—^	^—^	^—^	^—^	1A2^0^ 3A4^↓^ 2D6^0^ 2E1^0^	^—^	^—^	A^S^ B^S^	^—^	^—^	^—^	^↓^	^—^	^—^	^—^	[79,504,505,506]
Paeonol *	*P. lactiflora*	^—^	^—^	^—^	2^↓^	1A2^S^	^—^	^—^	A^↓^ B^↓^	^—^	^↓^	^—^	^S^	^—^	^—^	^—^	[507,508,509,510,511]
Palmatine	*C. chinensis* *C. speciosa*	^—^	^—^	^—^	2^↓^	1A1^↓^ 1A2^S^ 3A4^↑^ 2D6^S^	^—^	^—^	A^↓^	^—^	^—^	^—^	^↑^	^—^	^—^	^—^	[274,512,513,514,515]
Peonidin-3-*O*-β-arabinose *	*V. angustifolium*	^—^	^—^	^—^	^—^	^—^	^—^	^—^	^—^	^—^	^—^	^—^	^—^	^—^	^—^	^—^	—
d-Pinitol	*B. rufescens*	^—^	^—^	^—^	^—^	^—^	^—^	^—^	^—^	^—^	^—^	^—^	^—^	^—^	^—^	^—^	—
15-Methyl Pinusolidic acid *	*P. orientalis*	^—^	^—^	^—^	^—^	^—^	^—^	^—^	^—^	^A^	^—^	^—^	^—^	^—^	^—^	^—^	[133]
Phyllanthin *	*P. emblica*	^—^	^—^	^—^	^—^	1A2^0^ 3A4^↓^ 2C9^0^ 2D6^0^ 2E1^0^	^—^	^—^	^—^	^—^	^—^	2^0^	^↓^	^—^	^—^	^—^	[516,517]
Phytol	*M. lucida*	^—^	^↑^	^—^	2^↓^	^—^	^—^	^↓^	^—^	^—^	^—^	^—^	^—^	^—^	^—^	^—^	[518,519,520]
α-Pinene *	*R. officinalis* *S. lavandulaefolia*	^—^	^—^	^—^	2^↓^	2B6^↓^	^—^	^—^	^—^	^Ant^	^—^	^—^	^—^	^—^	^—^	^—^	[521,522,523]
Piperine *	*P. nigrum*	^—^	^—^	^—^	^—^	1A2^↑^ 3A4^↓^ 2C9^↓^ 2E1^↓^	^—^	^—^	^—^	^—^	^↓^	^—^	^↓^	^—^	22A2^↓^	1A1^↓^	[524,525,526]
Polygalaxanthone III *	*P. tenuifolia*	^—^	^—^	^—^	^—^	2E1^↓^	^—^	^—^	^—^	^—^	^—^	^—^	^—^	^—^	^—^	^—^	[527]
Protocatechuic acid *	*A. oxyphylla* *G. biloba* *M. officinalis* *R. officinalis* *V. vinifera*	^—^	^—^	^—^	^—^	1A1^↓^ 1A2^↓^ 2C9^↓^ 2E1^↓^	^—^	^—^	^—^	^—^	^—^	^—^	^—^	^—^	^—^	^—^	[245,528]
Prurienidine *	*M. pruriens*	^—^	^—^	^—^	^—^	^—^	^—^	^—^	^—^	^—^	^—^	^—^	^—^	^—^	^—^	^—^	—
Prurienine *	*M. pruriens*	^—^	^—^	^—^	^—^	^—^	^—^	^—^	^—^	^—^	^—^	^—^	^—^	^—^	^—^	^—^	—
Puerarin *	*P. lobata*	^—^	^↑^	^—^	2^↓^	1A1^↑^ 1A2^↑^ 3A4^↓^ 2B6^↓^ 2C9^↓^ 2D6^↓^ 2E1^↓^	^—^	^↓^	^—^	^↓^	^—^	^—^	^↑^	^—^	^—^	1A1^↑^	[529,530,531,532,533,534,535,536,537]
Quercetin *	*A. melegueta* *C. sativus* *C. rotundus* *H. perforatum* *I. paraguariensis* *L. meyenii* *M. lucida* *P. emblica* *R. nigrum* *S. costus* *V. uliginosum L.*	^—^	^—^	^—^	^—^	1A1^↓^ 1A2^↓^ 3A4^↓^ 2C8^↓^ 2C9^↓^ 2C19^↓^ 2D6^↓^	^—^	^—^	A^↓^	^Ant^	^↑S^	1^↓^ 2^↓^	^↓S^	^—^	21A6^↓^ 21A8^↓^ 21A9^↓^ 22A6^↓^	1A1^↑^ 1A6^↑^	[24,200,218,247,289,338,374,462,487,488,538,539,540,541,542]
Quercetin-3-*O*-β-d-glucuronide	*P. pterocarpum*	^—^	^—^	^—^	2^↓^	3A4^↓^	^—^	^—^	^—^	^—^	^—^	2^↓^	^↓^	^—^	21A8^↓^ 21A9^↓^	1A1^↓^	[539,543]
Resveratrol *	*V. vinifera*	^—^	^—^	^—^	^—^	1A1^↓S^ 1A2^↓^ 3A4^↓^ 2C9^↓^ 2C19^↓^ 2D6^↓^	^↑^	^↓^	A^↓^	^↓^	^↑S^	2^S^	^↓^	^—^	21A8^S^	^—^	[218,298,338,544,545,546,547,548,549,550,551]
Rosarin *	*R. rosea*	^—^	^—^	^—^	^—^	1A2^↓^ 3A4^↓^ 2C9^↓^ 2D6^↓^	^—^	^—^	A^0^ B^0^	^—^	^—^	^—^	^—^	^—^	^—^	^—^	[552,553]
Rosavin *	*R. rosea*	^—^	^—^	^—^	^—^	1A2^↓^ 3A4^↓^ 2C9^↓^ 2D6^↓^	^—^	^—^	A^0^ B^0^	^—^	^—^	^—^	^—^	^—^	^—^	^—^	[552,553]
Rosin *	*R. rosea*	^—^	^—^	^—^	^—^	1A2^↓^ 3A4^↓^ 2C9^↓^ 2D6^↓^	^—^	^—^	A^0^ B^0^	^—^	^—^	^—^	^—^	^—^	^—^	^—^	[552,553]
Rosmarinic acid *	*M. officinalis* *M. spicata* *R. officinalis*	^—^	^—^	^—^	^—^	3A4^↓^ 2C9^↓^ 2C19^↓^ 2D6^↓^ 2E1^↓^	^—^	^—^	^—^	^—^	^↓^	^—^	^↓^	^—^	^—^	1A1^↓^	[416,554,555]
Rutin *	*H. perforatum* *L. bicolor* *M. chamomilla* *M. flexuosa* *M. lucida* *M. peregrina* *V. uliginosum L.*	^—^	^↑^	^—^	2^↓^	1A1^↑^ 3A4^↓^	^—^	^↓^	B^↓^	^—^	^↓^	^—^	^↓^	^—^	21A6^↓^ 21A8^↓^ 21A9^↓↑^	^—^	[24,248,253,345,426,556,557,558,559,560,561,562,563]
Safranal *	*C. sativus*	^—^	^—^	^—^	^—^	2B6^↑^	^—^	^—^	^—^	^—^	^↓^	^—^	^↓^	^—^	^—^	^—^	[247,328,564]
Salidroside *	*R. rosea*	^↓^	^—^	^—^	1^↓^ 2^↓^	1A2^↑^ 3A4^↓^ 2B6^↑^ 2C9^↑^	^—^	^—^	A^↓^ B^↓^	^↑^	^—^	1^↓^	^↓^	^↓^	^—^	^—^	[565,566,567,568,569,570]
Salvianolic acid *	*M. spicata* *S. miltiorrhiza*	^—^	^—^	^—^	2^↓^	1A2^↓^ 3A4^↓^	^—^	^—^	^—^	^—^	^↑^	^—^	^↓^	^—^	21A8^S^	^—^	[571,572,573,574]
Sanguinine	*E. grandiflora* *P. illyricum*	^—^	^—^	^—^	^—^	3A4^0^	^—^	^—^	^—^	^—^	^—^	^—^	^—^	^—^	^—^	^—^	[399]
Sarcocine	*S. saligna*	^—^	^—^	^—^	^—^	^—^	^—^	^—^	^—^	^—^	^—^	^—^	^—^	^—^	15A1^S^ 15A2^S^	^—^	[575]
Sarcodine	*S. saligna*	^—^	^—^	^—^	2^↓^	^—^	^—^	^—^	^—^	^—^	^—^	^—^	^—^	^—^	^—^	^—^	[430]
Sarcorine	*S. saligna*	^—^	^—^	^—^	^—^	^—^	^—^	^—^	^—^	^—^	^—^	^—^	^—^	^—^	^—^	^—^	—
Sarsalignenone	*S. saligna*	^—^	^—^	^—^	2^↓^	^—^	^—^	^—^	^—^	^—^	^—^	^—^	^—^	^—^	^—^	^—^	[430]
Sarsasapogenin *	*A. racemosus*	^—^	^—^	^—^	^—^	^—^	^—^	^—^	A^↓^ B^↓^	^—^	^—^	^—^	^—^	^—^	21A6^S^	^—^	[369,576]
Schisandrin B *	*S. chinensis*	^—^	^—^	^—^	^—^	3A4^↓^ 3A5^↓^ 2B6^↑^ 2E1^S^	^—^	^—^	^—^	^—^	^—^	^—^	^↓^	^—^	21A6^↑^	^—^	[577,578,579,580,581]
Shatavarin IV *	*A. adscendens* *A. racemosus*	^—^	^—^	^—^	^—^	^—^	^—^	^—^	A^↓^ B^↓^	^—^	^—^	^—^	^—^	^—^	^—^	^—^	[582]
Shogaol *	*A. melegueta* *Z. officinale Rosc.*	^—^	^—^	^—^	1^↓^ 2^↓^	1A2^↓^ 2C9^↓^ 2C19^↓^	^—^	^—^	^—^	^↓^	^—^	^—^	^—^	^—^	^—^	1A1^S^ 1A3^S^ 2B7^S^	[583,584,585,586]
Sitoindosides VII-X *	*W. somnifera*	^—^	^—^	^—^	^—^	^—^	^—^	^—^	^—^	^—^	^—^	^—^	^—^	^—^	^—^	^—^	—
β-Sitosterol *	*A. lancea* *C. pluricaulis* *M. peregrina* *M. pruriens*	^—^	^↑^	^—^	2^↓^	3A4^0^ 3A5^0^ 2C19^0^	^—^	^↓^	^—^	^—^	^↓^	^—^	^0^	^—^	^—^	^—^	[587,588,589,590,591,592]
Spirofornabuxine	*B. hyrcana*	^—^	^—^	^—^	^—^	^—^	^—^	^—^	^—^	^—^	^—^	^—^	^—^	^—^	^—^	^—^	—
Stigmasterol *	*A. lancea*	^—^	^—^	^—^	^—^	3A4^↓^ 3A5^↓^	^—^	^—^	^—^	^—^	^—^	^—^	^↓^	^—^	^—^	^—^	[590,593]
Syringin *	*E. senticosus*	-	-	-	2^↓^	2C9^↓^ 2E1^↓^	^—^	^—^	^—^	^—^	^—^	^—^	^S^	^—^	^—^	^—^	[93,594]
Tannic acid *	*T. chebula*	^—^	^—^	^—^	2^↓^	1A2^↓^ 3A4^↓^ 2B6^↓^	^—^	^—^	^—^	^—^	^—^	1^↓^ 2^↓^	^↓^	^—^	21A6^S↓^	^—^	[333,369,595,596,597,598]
Tanshinone I *	*S. miltiorrhiza*	^—^	^—^	^—^	^—^	1A1^↑^ 1A2^↑↓^ 2C9^↓^ 2E1^↓^	^—^	^—^	^—^	^—^	^↓^	^—^	^S↓^	^—^	^—^	1A1^o^ 1A3^0^ 1A9^0^ 2B7^0^	[24,599,600,601,602]
Tanshinone IIA *	*S. miltiorrhiza*	^—^	^—^	^—^	2^↓^	1A1^↑^ 1A2^↑↓^ 3A4^↑↓^ 2C9^↓^ 2E1^↓^	^—^	^—^	^—^	^—^	^↓^	^—^	^↓^	^—^	^—^	1A1^o^ 1A3^0^ 1A9^0^ 2B7^0^	[24,599,600,601,603]
Tanshinone IIB *	*S.miltiorrhiza*	^—^	^—^	^—^	^—^	^—^	^—^	^—^	^—^	^—^	^—^	^—^	^—^	^—^	^—^	^—^	—
Tenuifolin *	*P.tenuifolia*	^—^	^—^	^—^	2^↓^	^—^	^—^	^—^	^—^	^—^	^—^	^—^	^—^	^—^	^—^	^—^	[604]
Tenuifoliside B *	*P. tenuifolia*	^—^	^—^	^—^	^—^	^—^	^—^	^—^	^—^	^—^	^—^	^—^	^—^	^—^	^—^	^—^	—
Tenuifoliside C *	*P. tenuifolia*	^—^	^—^	^—^	^—^	2E1^↓^	^—^	^—^	^—^	^—^	^—^	^—^	^—^	^—^	^—^	^—^	[527]
Theacrine *	*C. arabica* *T. grandiflorum*	^—^	^—^	^—^	^—^	^—^	^—^	^—^	^—^	^—^	^—^	^—^	^—^	^—^	^—^	^—^	—
Theobromine *	*T. cacao*	^—^	^—^	^—^	^—^	1A2^S^ 3A4^0^ 2C9^0^ 2C19^0^ 2D6^0^	^—^	^—^	^—^	^—^	^—^	^—^	^0^	^—^	^—^	^—^	[218,605]
Timosaponin AIII *	*A. asphodeloides*	^—^	^↑^	^—^	2^↓^	^—^	^—^	^—^	^—^	^—^	^—^	^—^	^↓^	^—^	^—^	^—^	[606]
Trigonelline *	*T. foenum-graecum*	^—^	^—^	^—^	^—^	1A2^↓^ 3A4^0^ 2B6^0^ 2C8^0^ 2C19^0^ 2D6^↓^ 2E1^0^	^—^	^↑^	^—^	^—^	^↑^	^—^	^—^	^—^	^—^	^—^	[607,608]
Ursolic acid *	*R. officinalis*	^—^	^—^	^—^	2^↓^	1A2^↓^ 3A4^↓S^ 2C8^↓^ 2C9^↓S^ 2C19^↓^ 2D6^↓^	^—^	^—^	A^↓^	^—^	^↓^	1^0^	^—^	^—^	21A6^↓^ 21A8^↓^ 21A9^↓^ 22A8^↓^	^—^	[276,409,501,609,610,611,612,613]
Vaganine	*S. saligna*	^—^	^—^	^—^	^—^	^—^	^—^	^—^	^—^	^—^	^—^	^—^	^—^	^—^	^—^	^—^	—
(+)-α-Viniferin *	*C. chamiague*	^—^	^—^	^—^	^—^	3A4^S^	^—^	^—^	^—^	^—^	^—^	^—^	^S^	^—^	^—^	^—^	[218]
Vinpocetine *	*V. minor*	^—^	^—^	^—^	^—^	3A4^S^	^—^	^—^	^—^	^—^	^—^	^—^	^—^	^—^	^—^	^—^	[614]
Withaferin A *	*W. somnifera*	^—^	^—^	^—^	^—^	1A1^↓^	^—^	^—^	^—^	^—^	^—^	^—^	^—^	^—^	^—^	^—^	[615]
Withanolide A *	*W. somnifera*	^—^	^—^	^—^	^—^	1A1^↓^	^—^	^—^	^—^	^—^	^—^	^—^	^—^	^—^	^—^	^—^	[615]
Withanolide B *	*W. somnifera*	^—^	^—^	^—^	^—^	1A1^↓^	^—^	^—^	^—^	^—^	^—^	^—^	^—^	^—^	^—^	^—^	[615]
Z-Ligustilide *	*A. sinensis*	^—^	^—^	^—^	^—^	1A1^↓^ 3A4^↑^ 2D6^↑^	^—^	^—^	^—^	^—^	^—^	^—^	^—^	^—^	^—^	^—^	[246,616]

*—Phytochemicals found in food supplements; ^↓^—target inhibition; ^↑^—target induction; ^s^—bioactive is substrate of the target; ^Ant^—Bioactive is a target antagonist; ^0^—No effect; (-)—Not reported; ABC—ATP-binding cassette; ALOX5—Arachidonate 5-lipoxygenase; AMPK—AMP-activated protein kinase; BCRP—Breast cancer resistance protein; BSEP—Bile salt export pump (ABCB11); COX—Cyclooxygenase; CYP—Cytochrome P450; GLP-1—Glucagon-like peptide-1; HMGCoAR—3-hydroxy-3-methyl-glutaril-CoA reductase; MAO—Monoamine Oxidase; MATE—Multi-antimicrobial extrusion protein; MRP—Multidrug resistance-associated protein; NMDA—*N*-methyl-d-aspartate; OAT—Organic anion transporter; OATP—Organic-anion-transporting polypeptide; OCT—Organic cation transport; P-gP—Glycoprotein P; UGT—Uridine diphosphate-glucuronosyltransferase; PLA2G2A—Phospholipase A2 Group IIA; SLC—Solute Carriers (15A/PEPT; 22A1-3/OCT1-3; 22A4/OCTN1; 22A6,8,11/OAT1,3,4; 47A/MATE; 21A3/OATP1A2; 21A6,8/OATP1B1,3; 21A9/OATP2B1; 21A14/OATP1C1); PEPT—Peptide transporter; association of two symbols (^↑↓^ or ^↓0^ or ^↑0^) for the same target, refers to conflicting information reported in literature.

**Table 4:** Hypothesized interactions between selected bioactives (those inducing/inhibiting at least four of the 55 targets studied) and drugs. Interaction was considered if at least one target was simultaneously affected by bioactive and drug, while sequentially fulfilling criteria 1 and 2 (see text for details). Piracetam is not shown, since it does not interact with any of the bioactives under study.

Bioactive	Drug								
	Pr	Al	Se	Mt	Di	At	Me	Ta	Cl
Apigenin	x	x	x	x	x	x	x	x	x
α-Asarone	x	x	x	-	x	x	x	x	x
β-Asarone	x	x	x	▲	x	x	-	x	x
Bacoside A	x	x	x	-	x	x	▼	x	x
Bacoside B	x	x	x	-	x	x	▼	x	x
Berberine	x	x	x	x	x	x	-	x	x
Biapigenin	x	x	x	-	x	x	x	x	x
Bilobalide	x	x	x	-	x	x	x	x	x
Biochanin A	x	x	x	-	x	x	x	x	x
Caffeic acid	x	x	x	x	x	x	▼	x	x
β-Carbolines (Harmine)	x	x	x	x	x	x	▼	-	x
9-Methyl-9B-Carboline	x	x	x	-	x	x	-	x	x
Catalpol	x	x	x	-	x	x	-	x	x
Catechin	x	x	x	x	x	x	x	x	x
Chlorogenic acid	x	-	x	▲	x	x	▼	-	x
Cinnamaldehyde	x	x	x	▲	x	x	x	x	x
Coptisine	x	x	x	x	x	▲	x	x	x
Crocin	x	x	x	-	x	x	▼	x	x
Curcumin	x	x	x	x	x	x	▼	x	x
Cyanidin-3-*O*-β-glucoside	x	x	x	-	x	x	-	x	x
Daidzein	x	x	x	x	x	x	-	x	x
Decursin	x	-	x^&^	-	x	x	-	x	x
Delphinidin	x	x	x	x	x	x	▼	x	x
Ellagic acid	▼	-	x	x	x	x	▼	-	x
ECG	x	x	x	-	x	x	-	x	x
EGCG	x	x	x	x	x	x	x	x	x
Eugenol	▼	-	x	◄►	▼	x	x	-	x
Ferulic acid	x	x	x	-	x	x	x	x	x
Forskolin	x	x	x	▲	x	x	x	x	x
Gallic acid	x	x	x	-	x	x	-	x	x
Genistein	x	x	x	x	x	x	x	x	x
Gingerol	x	x	x	-	x	x	▼	x	x
Ginkgolide A	x	x	x	-	x	x	▼	x	x
Ginkgolide B	x	x	x	-	x	x	▼	x	x
Ginsenoside Rb	x	x	x	x	x	x	-	x	x
Ginsenoside Rd	x	x	x	x	x	x	▼	x	x
Ginsenoside Rg	x	x	x	-	x	x	-	x	x
Glycyrrhizin	x	x	x	-	x	x	-	x	x
Hyperforin	x	x	x	x	x	x	x	x	x
Hypericin	x	x	x	x	x	x	x	x	x
Isoliquiritigenin	x	x	x	-	x	x	▼	x	x
Isorhamnetin	x	x	x	-	x	x	-	x	x
p-Kaempferol	x	x	x	-	x	x	x	x	x
Luteolin	x	x	x	x	x	x	▼	x	x
Malvidin 3-galactoside	x	x	x	x	x	x	-	x	x
Malvidin 3-glucoside	x	x	x	x	x	x	-	x	x
Mangiferin	x	x	x	-	x	x	▼	x	x
Myricetin	x	x	x	x	x	x	-	x	x
Naringenin	x	x	x	x	x	x	▼	x	x
Naringin	x	x	x	x	x	x	-	x	x
Nobiletin	x	x	x	x	x	x	-	x	x
Oleanolic acid	x	x	x	x	x	x	-	x	x
Paeonol	▼	-	x	x	x	x	-	-	-
Palmatine	x	x	x	-	x	x	-	x	x
Piperine	x	x	x	x	x	x	x	x	x
Protocatechuic acid	x	x	x	-	x	◄►	-	-	x
Puerarin	x	x	x	▲	x	x	▼	x	x
Quercetin	x	x	x	x	x	x	x	x	x
Quercetin-3-*O*-β-d-glucuronide	x	x	x	-	x	x	-	x	x
Resveratrol	x	x	x	x	x	x	x	x	x
Rosarin	x	x	x	-	x	x	-	x	x
Rosavin	x	x	x	-	x	x	-	x	x
Rosin	x	x	x	-	x	x	-	x	x
Rosmarinic acid	x	x	x	x	x	x	▼	x	x
Rutin	x	x	x	x	x	x	-	x	x
Salidroside	x	x	x	-	x	x	x	x	x
Salvianolic acid	x	x	x	x	x	x	-	x	x
Schisandrin B	x	x	x	-	x	x	▲	x	x
Shogaol	x	x	x	-	x	▼	x	-	x
β-Sitosterol	-	-	x^#^	x	x	x	-	-	-
Tannic acid	x	x	x	-	x	x	▼	x	x
Tanshinone I	x	x	x	x	x	x	-	x	x
Tanshinone IIA	x	x	x	x	x	x	-	x	x
Trigonelline	x	-	x	x	x	x	-	-	x
Ursolic acid	x	x	x	x	x	x	▼	x	x

X—Drug is substrate of at least one target, which is induced/inhibited by the bioactive; direction of interaction is not disclosed; x^&^—The bioactive only inhibits P-gP; x^#^—The bioactive only inhibits BCRP; ▼—Drug and bioactive are inhibitors of at least one shared target; ▲—Drug and bioactive are inducers of at least one shared target; ◄►—Drug and bioactive act at least in one target, in opposite direction; ㇐ no interaction in any of the targets.

## Supplementary Materials

The following are available online at https://www.mdpi.com/1999-4923/13/1/124/s1, Figure S1: Number of drugs interacting with the different targets (enzymes, transporters and receptors) as substrates, inducers/upregulators and inhibitors/downregulators/antagonists. Figure S2. Frequency of target modulation by bioactives, as a measure of HDI potential.

## Abbreviations

ABC	ATP-binding cassette
ABCG2	ATP Binding Cassette Subfamily G Member 2
AChE	Acetylcholinesterase
ADME	Absorption, distribution, metabolism and elimination
Al	Alprazolam
ALOX5	Arachidonate 5-lipoxygenase
AMPK	AMP-activated protein kinase
At	Atorvastatin
ATC	Anatomical Therapeutic Chemical
BChE	Butyrylcholinesterase
BCRP	Breast cancer resistance protein
BDDCS	Biopharmaceutics Drug Disposition Classification System
BSEP	Bile salt export pump
Cl	Clopidogrel
COMT	Catechol-*O*-methyltransferase
COX	Cyclooxygenase
CYP	Cytochrome P450
Di	Diclofenac
ECG	Epicatechin gallate
EGCG	Epigallocatechin-3-gallate
GABA	γ-Aminobutyric acid
GLP-1	Glucagon-like peptide-1
HAM	High-Alert Medications
HDI	Herb–drug interactions
5-HI	5-hydroxyndoleacetic acid
5-HT	5-hydroxytryptamine
HMC	Herbal Medicines Compendium
HMGCoAR	3-hydroxy-3-methyl-glutaril-CoA reductase;
IP	Indian Pharmacopoeia
ITC	International Transporter Consortium
JP	The Japanese Pharmacopoeia
L-DOPA	Levodopa
MAO	Monoamine oxidase
MATE	Multi-antimicrobial extrusion protein
MDR1	Multidrug resistance protein 1
Me	Memantine
MRP	Multidrug resistance-associated protein
Mt	Metformin
NMDA	*N*-methyl-d-aspartate
OAT	Organic anion transporter
OATP	Organic-anion-transporting polypeptide
OCT	Organic cation transport
PEPT	Peptide transporter
P-gP	Glycoprotein P
Ph.Eur	European Pharmacopoeia
Pi	Piracetam
PLA2G2A	Phospholipase A2 Group IIA
PPRC	Pharmacopoeia of People’s Republic of China
Pr	Propranolol
Se	Sertraline
SLC	Solute Carriers
SLCO	Solute carrier organic anion transporter
Ta	Tadalafil
UGT	Uridine diphosphate-glucuronosyltransferase
USP	United States Pharmacopoeia
WHO	World Health Organization

## References

[1] Winblad B., Amouyel P., Andrieu S., Ballard C., Brayne C., Brodaty H., Cedazo-Minguez A., Dubois B., Edvardsson D., Feldman H. (2016). Defeating Alzheimer’s disease and other dementias: A priority for European science and society. Lancet Neurol..

[2] Wu Y.-T., Fratiglioni L., Matthews F.E., Lobo A., Breteler M.M.B., Skoog I., Brayne C. (2016). Dementia in western Europe: Epidemiological evidence and implications for policy making. Lancet Neurol..

[3] Prince M., Ali G.-C., Guerchet M., Prina A.M., Albanese E., Wu Y.-T. (2016). Recent global trends in the prevalence and incidence of dementia, and survival with dementia. Alzheimers. Res. Ther..

[4] Dembitsky V.M., Dzhemileva L., Gloriozova T., D’yakonov V. (2020). Natural and synthetic drugs used for the treatment of the dementia. Biochem. Biophys. Res. Commun..

[5] Howes M.-J.R., Perry E.K., Perry N.S.L., Vásquez-londoño C. (2020). Role of phytochemicals as nutraceuticals for cognitive functions affected in ageing. Br. J. Pharmacol..

[6] Dominguez L.J., Barbagallo M. (2018). Nutritional prevention of cognitive decline and dementia. Acta BioMed.

[7] Solfrizzi V., Agosti P., Lozupone M., Custodero C., Schilardi A., Valiani V., Santamato A., Sardone R., Dibello V., Di Lena L. (2018). Nutritional interventions and cognitive-related outcomes in patients with late-life cognitive disorders: A systematic review. Neurosci. Biobehav. Rev..

[8] Liu Y., Wang S., Kan J., Zhang J., Zhou L., Huang Y., Zhang Y. (2020). Chinese Herbal Medicine Interventions in Neurological Disorder Therapeutics by Regulating Glutamate Signaling. Curr. Neuropharmacol..

[9] Shen J., Xu L., Qu C., Sun H., Zhang J. (2018). Resveratrol prevents cognitive deficits induced by chronic unpredictable mild stress: Sirt1/miR-134 signalling pathway regulates CREB/BDNF expression in hippocampus in vivo and in vitro. Behav. Brain Res..

[10] Sowndhararajan K., Kim S. (2017). Neuroprotective and cognitive enhancement potentials of *Angelica gigas* nakai root: A review. Sci. Pharm..

[11] Tan C.-C., Yu J.-T., Wang H.-F., Tan M.-S., Meng X.-F., Wang C., Jiang T., Zhu X.-C., Tan L. (2014). Efficacy and safety of donepezil, galantamine, rivastigmine, and memantine for the treatment of Alzheimer’s disease: A systematic review and meta-analysis. J. Alzheimers. Dis..

[12] Kasture S., Mohan M., Kasture V. (2013). *Mucuna pruriens* seeds in treatment of Parkinson’s disease: Pharmacological review. Orient. Pharm. Exp. Med..

[13] Cassani E., Cilia R., Laguna J., Barichella M., Contin M., Cereda E., Isaias I.U., Sparvoli F., Akpalu A., Budu K.O. (2016). *Mucuna pruriens* for Parkinson’s disease: Low-cost preparation method, laboratory measures and pharmacokinetics profile. J. Neurol. Sci..

[14] (2013). World Health Organization Global Action Plan for the Prevention and Control of Noncommunicable Diseases 2013–2020.

[15] Sut S., Baldan V., Faggian M., Peron G., Dall Acqua S. (2016). Nutraceuticals, A New Challenge for Medicinal Chemistry. Curr. Med. Chem..

[16] Tundis R., Loizzo M.R., Nabavi S.M., Orhan I.E., Skalicka-Woźniak K., D’Onofrio G., Aiello F., Brahmachari G. (2018). Chapter 3—Natural Compounds and Their Derivatives as Multifunctional Agents for the Treatment of Alzheimer Disease. Discovery and Development of Neuroprotective Agents from Natural Products.

[17] López S., Bastida J., Viladomat F., Codina C. (2002). Acetylcholinesterase inhibitory activity of some Amaryllidaceae alkaloids and Narcissus extracts. Life Sci..

[18] Lin Z., Gu J., Xiu J., Mi T., Dong J., Tiwari J.K. (2012). Traditional chinese medicine for senile dementia. Evid. Based Complement. Altern. Med..

[19] Kennedy D.O., Wightman E.L. (2011). Herbal extracts and phytochemicals: Plant secondary metabolites and the enhancement of human brain function. Adv. Nutr..

[20] Perry E., Howes M.-J.R. (2011). Medicinal plants and dementia therapy: Herbal hopes for brain aging?. CNS Neurosci. Ther..

[21] Wightman E.L. (2017). Potential benefits of phytochemicals against Alzheimer’s disease. Proc. Nutr. Soc..

[22] Ahmed F., Ghalib R.M., Sasikala P., Ahmed K.K.M. (2013). Cholinesterase inhibitors from botanicals. Pharmacogn. Rev..

[23] Hostettmann K., Borloz A., Urbain A., Marston A. (2006). Natural product inhibitors of acetylcholinesterase. Curr. Org. Chem..

[24] Zhou X., Chan K., Yeung J.H.K. (2012). Herb-drug interactions with Danshen (*Salvia miltiorrhiza*): A review on the role of cytochrome P450 enzymes. Drug Metabol. Drug Interact..

[25] Clairet A.-L., Boiteux-Jurain M., Curtit E., Jeannin M., Gérard B., Nerich V., Limat S. (2019). Interaction between phytotherapy and oral anticancer agents: Prospective study and literature review. Med. Oncol..

[26] Shaikh A.S., Thomas A.B., Chitlange S.S. (2020). Herb-drug interaction studies of herbs used in treatment of cardiovascular disorders-A narrative review of preclinical and clinical studies. Phytother. Res..

[27] Kantor E.D., Rehm C.D., Haas J.S., Chan A.T., Giovannucci E.L. (2015). Trends in Prescription Drug Use Among Adults in the United States From 1999–2012. JAMA.

[28] Payne R.A. (2016). The epidemiology of polypharmacy. Clin. Med..

[29] Council of Europe (2019). European Pharmacopoeia (up to 10.2).

[30] United States Pharmacopoeia Convention Inc. (2019). United States Pharmacopeia National Formulary USP43 NF38.

[31] Society of Japanese Pharmacopoeia (2016). The Japanese Pharmacopoeia (English Version).

[32] Indian Pharmacopoeia Commission (2018). Indian Pharmacopoeia 2018.

[33] Chinese Pharmacopoeia Commission (2015). Pharmacopoeia of the People’s Republic of China.

[34] WHO Monographs on Selected Medicinal Plants. https://apps.who.int/iris/handle/10665/42052.

[35] Herbal Medicines Compendium. https://hmc.usp.org/.

[36] World Health Organization WHOCC—ATC/DDD Index. https://www.whocc.no/atc_ddd_index/.

[37] Golfar Y., Shayanfar A. (2019). Prediction of Biopharmaceutical Drug Disposition Classification System (BDDCS) by Structural Parameters. J. Pharm. Pharm. Sci..

[38] Benet L.Z., Broccatelli F., Oprea T.I. (2011). BDDCS applied to over 900 drugs. AAPS J..

[39] Mora M.J., Onnainty R., Granero G.E. (2018). Comparative Oral Drug Classification Systems: Acetazolamide, Azithromycin, Clopidogrel, and Efavirenz Case Studies. Mol. Pharm..

[40] Benet L.Z. (2013). The role of BCS (biopharmaceutics classification system) and BDDCS (biopharmaceutics drug disposition classification system) in drug development. J. Pharm. Sci..

[41] Zheng Y., Chen X., Benet L.Z. (2016). Reliability of In Vitro and In Vivo Methods for Predicting the Effect of P-Glycoprotein on the Delivery of Antidepressants to the Brain. Clin. Pharmacokinet..

[42] Zheng Y., Benet L.Z., Okochi H., Chen X. (2015). pH Dependent but not P-gp Dependent Bidirectional Transport Study of S-propranolol: The Importance of Passive Diffusion. Pharm. Res..

[43] Hosey C.M., Chan R., Benet L.Z. (2016). BDDCS Predictions, Self-Correcting Aspects of BDDCS Assignments, BDDCS Assignment Corrections, and Classification for more than 175 Additional Drugs. AAPS J..

[44] Institute for Safe Medication Practices (2020). ISMP List of High-Alert Medications in Acute Care Settings 2018.

[45] Maroyi A. (2018). *Albizia adianthifolia*: Botany, Medicinal Uses, Phytochemistry, and Pharmacological Properties. Sci. World J..

[46] Sonibare M.A., Ayoola I.O., Elufioye T.O. (2017). Antioxidant and acetylcholinesterase inhibitory activities of leaf extract and fractions of *Albizia adianthifolia* (Schumach) W.F. Wright. J. Basic Clin. Physiol. Pharmacol..

[47] Toukea D.D., Kamto E.L.D., Simo L.M., Mbing J.N., Antheaume C., Haddad M., Noté O.P., Pegnyemb D.E. (2020). New triterpenoid saponin from the stems of *Albizia adianthifolia* (Schumach.) W.Wight. Nat. Prod. Res..

[48] Ferraz A.C., Angelucci M.E., da Costa M.L., Batista I.R., de Oliveira B.H., da Cunha C. (1999). Pharmacological evaluation of ricinine, a central nervous system stimulant isolated from *Ricinus communis*. Pharmacol. Biochem. Behav..

[49] Patel K., Patel D.K. (2016). Medicinal significance, pharmacological activities, and analytical aspects of ricinine: A concise report. J. Coast. Life Med..

[50] Popiołek A.K., Chyrek-Tomaszewska A., Stachowicz-Karpińska A., Bieliński M.K., Borkowska A. (2020). Biochemical parameters in cognitive functions. Neuropsychiatr. Dis. Treat..

[51] Sekhar V.C., Viswanathan G., Baby S. (2019). Insights Into the Molecular Aspects of Neuroprotective Bacoside A and Bacopaside I. Curr. Neuropharmacol..

[52] Rezende D.A.D.C.S., das Graças Cardoso M., Souza R.V., Teixeira M.L., Brandão R.M., Ferreira V.R.F., e Nogueira J.O., Magalhães M.L., Marcussi S., Nelson D.L. (2017). Essential oils from *Mentha piperita*, *Cymbopogon citratus*, *Rosmarinus officinalis*, *Peumus boldus* and *Foeniculum vulgare*: Inhibition of phospholipase A2 and cytotoxicity to human erythrocytes. Am. J. Plant Sci..

[53] Gray N.E., Alcazar Magana A., Lak P., Wright K.M., Quinn J., Stevens J.F., Maier C.S., Soumyanath A. (2018). *Centella asiatica*—Phytochemistry and mechanisms of neuroprotection and cognitive enhancement. Phytochem. Rev..

[54] Rajabian A., Hosseini A., Hosseini M., Sadeghnia H.R. (2019). A review of potential efficacy of Saffron (*Crocus sativus* L.) in cognitive dysfunction and seizures. Prev. Nutr. Food Sci..

[55] Kumar H., More S.V., Han S.-D., Choi J.-Y., Choi D.-K. (2012). Promising therapeutics with natural bioactive compounds for improving learning and memory—A review of randomized trials. Molecules.

[56] Sharma V., Singh I., Chaudhary P. (2014). *Acorus calamus* (The Healing Plant): A review on its medicinal potential, micropropagation and conservation. Nat. Prod. Res..

[57] Shiksharthi A.R., Mittal S., Ramana J. (2011). Systematic review of herbals as potential memory enhancers. Int. J. Herb. Med..

[58] Pak M.E., Kim Y.R., Kim H.N., Ahn S.M., Shin H.K., Baek J.U., Choi B.T. (2016). Studies on medicinal herbs for cognitive enhancement based on the text mining of Dongeuibogam and preliminary evaluation of its effects. J. Ethnopharmacol..

[59] Ishola I.O., Awoyemi A.A., Afolayan G.O. (2016). Involvement of antioxidant system in the amelioration of scopolamine-induced memory impairment by Grains of Paradise (*Aframomum melegueta* K. Schum.) extract. Drug Res..

[60] Adefegha S.A., Oboh G. (2012). Acetylcholinesterase (AChE) inhibitory activity, antioxidant properties and phenolic composition of two Aframomum species. J. Basic Clin. Physiol. Pharmacol..

[61] Shi S.-H., Zhao X., Liu B., Li H., Liu A.-J., Wu B., Bi K.-S., Jia Y. (2014). The effects of sesquiterpenes-rich extract of *Alpinia oxyphylla* Miq. on amyloid-β-induced cognitive impairment and neuronal abnormalities in the cortex and hippocampus of mice. Oxid. Med. Cell. Longev..

[62] Wei W.-L., Zeng R., Gu C.-M., Qu Y., Huang L.-F. (2016). Angelica sinensis in China-A review of botanical profile, ethnopharmacology, phytochemistry and chemical analysis. J. Ethnopharmacol..

[63] Wu Y.C., Hsieh C.L. (2011). Pharmacological effects of Radix *Angelica sinensis* (Danggui) on cerebral infarction. Chin. Med..

[64] Khan I., Nisar M., Khan N., Saeed M., Nadeem S., Ali F., Karim N., Kaleem W.A., Qayum M., Ahmad H. (2010). Structural insights to investigate Conypododiol as a dual cholinesterase inhibitor from *Asparagus adscendens*. Fitoterapia.

[65] Pahwa P., Goel R.K. (2016). *Asparagus adscendens* root extract enhances cognition and protects against scopolamine induced amnesia: An *in-silico* and *in-vivo* studies. Chem. Biol. Interact..

[66] Uddin M.S., Al Mamun A., Kabir M.T., Jakaria M., Mathew B., Barreto G.E., Ashraf G.M. (2019). Nootropic and Anti-Alzheimer’s Actions of Medicinal Plants: Molecular Insight into Therapeutic Potential to Alleviate Alzheimer’s Neuropathology. Mol. Neurobiol..

[67] Chen L.-G., Jan Y.-S., Tsai P.-W., Norimoto H., Michihara S., Murayama C., Wang C.-C. (2016). Anti-inflammatory and Antinociceptive Constituents of *Atractylodes japonica* Koidzumi. J. Agric. Food Chem..

[68] Dubey T., Chinnathambi S. (2019). Brahmi (*Bacopa monnieri*): An ayurvedic herb against the Alzheimer’s disease. Arch. Biochem. Biophys..

[69] Peth-Nui T., Wattanathorn J., Muchimapura S., Tong-Un T., Piyavhatkul N., Rangseekajee P., Ingkaninan K., Vittaya-Areekul S. (2012). Effects of 12-week *Bacopa monnieri* consumption on attention, cognitive processing, working memory, and functions of both cholinergic and monoaminergic systems in healthy elderly volunteers. Evid. Based Complement. Altern. Med..

[70] Devendra P., Patel S.S., Birwal P., Basu S., Deshmukh G., Datir R. (2018). Brahmi (*Bacopa monnieri*) as functional food ingredient in food processing industry. J. Pharmacogn. Phytochem..

[71] Muhammad A., Dauda U., Jibril S., Sirat H.M. (2019). Acetylcholinesterase inhibitory activity of a cyclitol isolated from the leaves of *Bauhinia rufescens*. BAJOPAS.

[72] Ata A., Iverson C.D., Kalhari K.S., Akhter S., Betteridge J., Meshkatalsadat M.H., Orhan I., Sener B. (2010). Triterpenoidal alkaloids from *Buxus hyrcana* and their enzyme inhibitory, anti-fungal and anti-leishmanial activities. Phytochemistry.

[73] Mancini E., Beglinger C., Drewe J., Zanchi D., Lang U.E., Borgwardt S. (2017). Green tea effects on cognition, mood and human brain function: A systematic review. Phytomedicine.

[74] Van Dusseldorp T.A., Stratton M.T., Bailly A.R., Holmes A.J., Alesi M.G., Feito Y., Mangine G.T., Hester G.M., Esmat T.A., Barcala M. (2020). Safety of Short-Term Supplementation with Methylliberine (Dynamine^®^) Alone and in Combination with TeaCrine^®^ in Young Adults. Nutrients.

[75] Keshavarz M., Farrokhi M.R., Amiri A., Hosseini M. (2019). The contribution of S100B to the glioprotective effects of valproic and arundic acids. Iran. J. Basic Med. Sci..

[76] Pham H.M., Xu A., Schriner S.E., Sevrioukov E.A., Jafari M. (2018). Cinnamaldehyde Improves Lifespan and Healthspan in Drosophila melanogaster Models for Alzheimer’s Disease. Biomed Res. Int..

[77] Irie Y. (2006). Effects of eugenol on the central nervous system: Its possible application to treatment of Alzheimer’s disease, depression, and Parkinson’s disease. CBC.

[78] Kumar S., Kumari R., Mishra S. (2019). Pharmacological properties and their medicinal uses of Cinnamomum: A review. J. Pharm. Pharmacol..

[79] Stohs S.J. (2017). Safety, Efficacy, and Mechanistic Studies Regarding *Citrus aurantium* (Bitter Orange) Extract and p-Synephrine. Phytother. Res..

[80] Bello M.L., Walker A.J., McFadden B.A., Sanders D.J., Arent S.M. (2019). The effects of TeaCrine^®^ and caffeine on endurance and cognitive performance during a simulated match in high-level soccer players. J. Int. Soc. Sports Nutr..

[81] Pomeroy D.E., Tooley K.L., Probert B., Wilson A., Kemps E. (2020). A systematic review of the effect of dietary supplements on cognitive performance in healthy young adults and military personnel. Nutrients.

[82] Owona B.A., Zug C., Schluesener H.J., Zhang Z.-Y. (2016). Protective effects of forskolin on behavioral deficits and neuropathological changes in a mouse model of cerebral amyloidosis. J. Neuropathol. Exp. Neurol..

[83] Amin H., Sharma R., Vyas M., Prajapati P.K., Dhiman K. (2014). *Shankhapushpi* (*Convolvulus pluricaulis* Choisy): Validation of the Ayurvedic therapeutic claims through contemporary studies. Int. J. Green Pharm..

[84] Kuo P.-C., Yang M.-L., Hwang T.-L., Lai Y.-Y., Li Y.-C., Thang T.D., Wu T.-S. (2013). Anti-inflammatory diterpenoids from *Croton tonkinensis*. J. Nat. Prod..

[85] Calderón-Montaño J.M., Burgos-Morón E., Pérez-Guerrero C., López-Lázaro M. (2011). A review on the dietary flavonoid kaempferol. Mini Rev. Med. Chem..

[86] Tohda C., Yang X., Matsui M., Inada Y., Kadomoto E., Nakada S., Watari H., Shibahara N. (2017). Diosgenin-rich yam extract enhances cognitive function: A placebo-controlled, randomized, double-blind, crossover study of healthy adults. Nutrients.

[87] Chiu C.-S., Chiu Y.-J., Wu L.-Y., Lu T.-C., Huang T.-H., Hsieh M.-T., Lu C.-Y., Peng W.-H. (2011). Diosgenin ameliorates cognition deficit and attenuates oxidative damage in senescent mice induced by D-galactose. Am. J. Chin. Med..

[88] Patocka J. (2019). Bioactivity of *Echium amoenum*: A mini review. BJSTR.

[89] Rabiei Z., Setorki M. (2018). Effect of hydroalcoholic *Echium amoenum* extract on scopolamine-induced learning and memory impairment in rats. Pharm. Biol..

[90] Kim Y.H., Cho M.L., Kim D.B., Shin G.H., Lee J.H., Lee J.S., Park S.O., Lee S.J., Shin H.M., Lee O.H. (2015). The antioxidant activity and their major antioxidant compounds from *Acanthopanax senticosus* and *A. koreanum*. Molecules.

[91] Kwan C.-Y., Zhang W.-B., Sim S.-M., Deyama T., Nishibe S. (2004). Vascular effects of Siberian ginseng (*Eleutherococcus senticosus*): Endothelium-dependent No- and EDHF-mediated relaxation depending on vessel size. Naunyn. Schmiedebergs. Arch. Pharmacol..

[92] Lee D., Park J., Yoon J., Kim M.-Y., Choi H.-Y., Kim H. (2012). Neuroprotective effects of *Eleutherococcus senticosus* bark on transient global cerebral ischemia in rats. J. Ethnopharmacol..

[93] Ahmed S., Moni D.A., Sonawane K.D., Paek K.Y., Shohael A.M. (2020). A comprehensive in silico exploration of pharmacological properties, bioactivities and COX-2 inhibitory potential of eleutheroside B from *Eleutherococcus senticosus* (Rupr. & Maxim.) Maxim. J. Biomol. Struct. Dyn..

[94] Joshi H., Parle M. (2006). Cholinergic basis of memory-strengthening effect of *Foeniculum vulgare* Linn. J. Med. Food.

[95] Sayah K., El Omari N., Kharbach M., Bouyahya A., Kamal R., Marmouzi I., Cherrah Y., Faouzi M.E.A. (2020). Comparative Study of Leaf and Rootstock Aqueous Extracts of *Foeniculum vulgare* on Chemical Profile and In Vitro Antioxidant and Antihyperglycemic Activities. Adv. Pharmacol. Pharm. Sci..

[96] Nemati M., Hemmati A.A., Najafzadeh H., Mansouri M.T., Khodayar M.J. (2018). Evaluation of the effects of *Foeniculum vulgare* essence on behavioral-motor disorders of Parkinson’s Disease induced by reserpine in ovariectomized and non ovariectomized rats. Jundishapur J. Nat. Pharm. Prod..

[97] Nguyen T., Alzahrani T. (2020). Ginkgo Biloba. StatPearls.

[98] Ben-Eliezer D., Yechiam E. (2016). *Hypericum perforatum* as a cognitive enhancer in rodents: A meta-analysis. Sci. Rep..

[99] Oliveira A.I., Pinho C., Sarmento B., Dias A.C.P. (2016). Neuroprotective Activity of *Hypericum perforatum* and Its Major Components. Front. Plant Sci..

[100] Widy-Tyszkiewicz E., Piechal A., Joniec I., Blecharz-Klin K. (2002). Long term administration of *Hypericum perforatum* improves spatial learning and memory in the water maze. Biol. Pharm. Bull..

[101] Dinamarca M.C., Cerpa W., Garrido J., Hancke J.L., Inestrosa N.C. (2006). Hyperforin prevents beta-amyloid neurotoxicity and spatial memory impairments by disaggregation of Alzheimer’s amyloid-beta-deposits. Mol. Psychiatry.

[102] Karioti A., Bilia A.R. (2010). Hypericins as potential leads for new therapeutics. Int. J. Mol. Sci..

[103] da Silva N.L.P., Cabrera L.P.B., Medeiros L.L.M., Formigoni M., Fuchs R.H.B., Droval A.A., Reitz F.A.C. (2020). Medicinal effects of Peruvian maca (*Lepidium meyenii*): A review. Food Funct..

[104] Wang S., Zhu F. (2019). Chemical composition and health effects of maca (*Lepidium meyenii*). Food Chem..

[105] Alasmari M., Böhlke M., Kelley C., Maher T., Pino-Figueroa A. (2019). Inhibition of Fatty Acid Amide Hydrolase (FAAH) by Macamides. Mol. Neurobiol..

[106] Almukadi H., Wu H., Böhlke M., Kelley C.J., Maher T.J., Pino-Figueroa A. (2013). The Macamide N-3-Methoxybenzyl-Linoleamide Is a Time-Dependent Fatty Acid Amide Hydrolase (FAAH) Inhibitor. Mol. Neurobiol..

[107] Gonzales G.F., Gonzales-Castañeda C. (2009). The Methyltetrahydro-beta-Carbolines in Maca (*Lepidium meyenii*). Evid. Based Complement. Altern. Med..

[108] López-Ríos L., Wiebe J.C., Vega-Morales T., Gericke N. (2020). Central nervous system activities of extract *Mangifera indica* L.. J. Ethnopharmacol..

[109] Nonato C.D.F.A., Leite D.O.D., Pereira R.C., Boligon A.A., Ribeiro-Filho J., Rodrigues F.F.G., da Costa J.G.M. (2018). Chemical analysis and evaluation of antioxidant and antimicrobial activities of fruit fractions of *Mauritia flexuosa* L. f. (Arecaceae). PeerJ.

[110] Ali-Shtayeh M.S., Jamous R.M., Zaitoun S.Y.A., Qasem I.B. (2014). *In-vitro* screening of acetylcholinesterase inhibitory activity of extracts from Palestinian indigenous flora in relation to the treatment of Alzheimer’s disease. Funct. Foods Health Dis..

[111] Herrlinger K.A., Nieman K.M., Sanoshy K.D., Fonseca B.A., Lasrado J.A., Schild A.L., Maki K.C., Wesnes K.A., Ceddia M.A. (2018). Spearmint Extract Improves Working Memory in Men and Women with Age-Associated Memory Impairment. J. Altern. Complement. Med..

[112] Qu J., Zhou Q., Du Y., Zhang W., Bai M., Zhang Z., Xi Y., Li Z., Miao J. (2014). Rutin protects against cognitive deficits and brain damage in rats with chronic cerebral hypoperfusion. Br. J. Pharmacol..

[113] Wang K., Sun W., Zhang L., Guo W., Xu J., Liu S., Zhou Z., Zhang Y. (2018). Oleanolic acid ameliorates Aβ25-35 injection-induced memory deficit in Alzheimer’s disease model rats by maintaining synaptic plasticity. CNS Neurol. Disord. Drug Targets.

[114] Heitman E., Ingram D.K. (2017). Cognitive and neuroprotective effects of chlorogenic acid. Nutr. Neurosci..

[115] Oboh G., Akomolafe T.L., Adefegha S.A., Adetuyi A.O. (2012). Attenuation of cyclophosphamide-induced neurotoxicity in rat by yellow dye extract from root of Brimstone tree (*Morinda lucida*). Exp. Toxicol. Pathol..

[116] Elufioye T.O., Hameed H.A. (2017). Cognitive-enhancing properties of *Morinda lucida* (Rubiaceae) and *Peltophorum pterocarpum* (Fabaceae) in scopolamine-induced amnesic mice. Afr. J. Tradit. Complement. Altern. Med..

[117] Chokki M., Cudălbeanu M., Zongo C., Dah-Nouvlessounon D., Ghinea I.O., Furdui B., Raclea R., Savadogo A., Baba-Moussa L., Avamescu S.M. (2020). Exploring Antioxidant and Enzymes (A-Amylase and B-Glucosidase) Inhibitory Activity of *Morinda lucida* and *Momordica charantia* Leaves from Benin. Foods.

[118] Elufioye T.O., Obuotor E., Agbedahunsi J.M., Adesanya S.A. (2015). Acetyl and Butyrylcholinesterase Inhibiting Constituent from *Morinda lucida* Benth (Rubiaceae). Br. J. Pharm. Res..

[119] Singh B., Sharma R.A. (2020). Indian Morinda species: A review. Phytother. Res..

[120] Sachan A., Singh S., Singh H., Shankar P., Kumar D., Sachan A.K., Nath R., Dixi R.K. (2015). An experimental study to evaluate the effect of *Mucuna pruriens* on learning and memory in mice. IJISR.

[121] Iannello C., Pigni N.B., Antognoni F., Poli F., Maxia A., de Andrade J.P., Bastida J. (2014). A potent acetylcholinesterase inhibitor from *Pancratium illyricum* L.. Fitoterapia.

[122] Konstantinos F., Heun R. (2019). The effects of Guarana (*Paullinia cupana*) supplementation on the cognitive performance of young healthy adults—A Systematic Review. Glob. Psychiatry.

[123] Heckman M.A., Weil J., Gonzalez de Mejia E. (2010). Caffeine (1, 3, 7-trimethylxanthine) in foods: A comprehensive review on consumption, functionality, safety, and regulatory matters. J. Food Sci..

[124] Herraiz T., Guillén H. (2018). Monoamine Oxidase-A Inhibition and Associated Antioxidant Activity in Plant Extracts with Potential Antidepressant Actions. Biomed Res. Int..

[125] Gruss M., Appenroth D., Flubacher A., Enzensperger C., Bock J., Fleck C., Gille G., Braun K. (2012). 9-Methyl-β-carboline-induced cognitive enhancement is associated with elevated hippocampal dopamine levels and dendritic and synaptic proliferation. J. Neurochem..

[126] Li S., Zhang Y., Deng G., Wang Y., Qi S., Cheng X., Ma Y., Xie Y., Wang C. (2017). Exposure characteristics of the analogous β-carboline alkaloids harmaline and harmine based on the efflux transporter of multidrug resistance protein 2. Front. Pharmacol..

[127] Keller S., Polanski W.H., Enzensperger C., Reichmann H., Hermann A., Gille G. (2020). 9-Methyl-β-carboline inhibits monoamine oxidase activity and stimulates the expression of neurotrophic factors by astrocytes. J. Neural Transm..

[128] Charoenteeraboon J., Ngamkitidechakul C., Soonthornchareonnon N., Jaijoy K., Sireeratawong S. (2010). Others Antioxidant activities of the standardized water extract from fruit of *Phyllanthus emblica* Linn. Songklanakarin J. Sci. Technol..

[129] Kumar R., Sharma S., Parihar L. (2020). Evaluation of memory enhancing potential of ethanolic extract of *Terminalia belerica* (EETB) aganinst scopolamine induced amnesia in Wistar rats. World J. Pharm. Pharm. Sci..

[130] Joshi H., Parle M. (2006). Evaluation of antiamnestic potentials of [6]-gingerol and phyllanthin in mice. Nat. Prod. Bioprospect..

[131] Chonpathompikunlert P., Wattanathorn J., Muchimapura S. (2010). Piperine, the main alkaloid of Thai black pepper, protects against neurodegeneration and cognitive impairment in animal model of cognitive deficit like condition of Alzheimer’s disease. Food Chem. Toxicol..

[132] Wightman E.L., Reay J.L., Haskell C.F., Williamson G., Dew T.P., Kennedy D.O. (2014). Effects of resveratrol alone or in combination with piperine on cerebral blood flow parameters and cognitive performance in human subjects: A randomised, double-blind, placebo-controlled, cross-over investigation. Br. J. Nutr..

[133] Silva A.R., Grosso C., Delerue-Matos C., Rocha J.M. (2019). Comprehensive review on the interaction between natural compounds and brain receptors: Benefits and toxicity. Eur. J. Med. Chem..

[134] Kim K.-S., Lee D.-S., Bae G.-S., Park S.-J., Kang D.-G., Lee H.-S., Oh H., Kim Y.-C. (2013). The inhibition of JNK MAPK and NF-κB signaling by tenuifoliside A isolated from *Polygala tenuifolia* in lipopolysaccharide-induced macrophages is associated with its anti-inflammatory effect. Eur. J. Pharmacol..

[135] Dong X.-Z., Huang C.-L., Yu B.-Y., Hu Y., Mu L.-H., Liu P. (2014). Effect of Tenuifoliside A isolated from *Polygala tenuifolia* on the ERK and PI3K pathways in C6 glioma cells. Phytomedicine.

[136] Liu Y.-M., Li Z.-Y., Hu H., Xu S.-P., Chang Q., Liao Y.-H., Pan R.-L., Liu X.-M. (2015). Tenuifolin, a secondary saponin from hydrolysates of polygalasaponins, counteracts the neurotoxicity induced by Aβ25-35 peptides in vitro and in vivo. Pharmacol. Biochem. Behav..

[137] Li Z., Liu Y., Wang L., Liu X., Chang Q., Guo Z., Liao Y., Pan R., Fan T.-P. (2014). Memory-Enhancing Effects of the Crude Extract of *Polygala tenuifolia* on Aged Mice. Evid. Based Complement. Altern. Med..

[138] Park C.H., Choi S.H., Koo J.-W., Seo J.-H., Kim H.-S., Jeong S.-J., Suh Y.-H. (2002). Novel cognitive improving and neuroprotective activities of *Polygala tenuifolia* Willdenow extract, BT-11. J. Neurosci. Res..

[139] Wu D., He J., Jiang Y., Yang B. (2015). Quality analysis of *Polygala tenuifolia* root by ultrahigh performance liquid chromatography-tandem mass spectrometry and gas chromatography-mass spectrometry. J. Food Drug Anal..

[140] Wu Y., Shi Q., Lei H., Liu X., Luan L. (2014). Studies on the total synthesis of tenuifoliside B. Tetrahedron.

[141] Huang H.-J., Huang C.-Y., Lee M., Lin J.-Y., Hsieh-Li H.M. (2019). Puerariae Radix prevents anxiety and cognitive deficits in mice under oligomeric Aβ-induced stress. Am. J. Chin. Med..

[142] Wang Z., Huang X., Zhao P., Zhao L., Wang Z.-Y. (2018). Catalpol inhibits amyloid-β generation through promoting α-cleavage of APP in Swedish mutant APP overexpressed N2a Cells. Front. Aging Neurosci..

[143] Leong P.K., Chen J., Ko K.M., Mandal S.C., Mandal V., Konishi T. (2018). Chapter 4—Development of Chinese herbal health products for the prevention of aging-associated diseases. Natural Products and Drug Discovery.

[144] Shiksharthi A.R., Mittal S., Ramana J., Road R., Pradesh H. (2011). Systematic Review of Herbals as Potential Memory Enhancers. Int. J. Res. Pharm. Biomed. Sci..

[145] Mao J., Huang S., Liu S., Feng X.-L., Yu M., Liu J., Sun Y.E., Chen G., Yu Y., Zhao J. (2015). A herbal medicine for Alzheimer’s disease and its active constituents promote neural progenitor proliferation. Aging Cell.

[146] Panossian A., Wikman G., Sarris J. (2010). Rosenroot (*Rhodiola rosea*): Traditional use, chemical composition, pharmacology and clinical efficacy. Phytomedicine.

[147] Cropley M., Banks A.P., Boyle J. (2015). The effects of *Rhodiola rosea* L. extract on anxiety, stress, cognition and other mood symptoms. Phytother. Res..

[148] Amsterdam J.D., Panossian A.G. (2016). *Rhodiola rosea* L. as a putative botanical antidepressant. Phytomedicine.

[149] Vepsäläinen S., Koivisto H., Pekkarinen E., Mäkinen P., Dobson G., McDougall G.J., Stewart D., Haapasalo A., Karjalainen R.O., Tanila H. (2013). Anthocyanin-enriched bilberry and blackcurrant extracts modulate amyloid precursor protein processing and alleviate behavioral abnormalities in the APP/PS1 mouse model of Alzheimer’s disease. J. Nutr. Biochem..

[150] Kennedy D.O., Dodd F.L., Robertson B.C., Okello E.J., Reay J.L., Scholey A.B., Haskell C.F. (2011). Monoterpenoid extract of sage (*Salvia lavandulaefolia*) with cholinesterase inhibiting properties improves cognitive performance and mood in healthy adults. J. Psychopharmacol..

[151] Chong C.-M., Su H., Lu J.-J., Wang Y. (2019). The effects of bioactive components from the rhizome of *Salvia miltiorrhiza* (Danshen) on the characteristics of Alzheimer’s disease. Chin. Med..

[152] Ren Y., Houghton P.J., Hider R.C., Howes M.-J.R. (2004). Novel diterpenoid acetylcholinesterase inhibitors from *Salvia miltiorhiza*. Planta Med..

[153] Kim S.R., Lee K.Y., Koo K.A., Sung S.H., Lee N.-G., Kim J., Kim Y.C. (2002). Four new neuroprotective iridoid glycosides from *Scrophularia buergeriana* roots. J. Nat. Prod..

[154] Kim S.R., Koo K.A., Sung S.H., Ma C.J., Yoon J.S., Kim Y.C. (2003). Iridoids from *Scrophularia buergeriana* attenuate glutamate-induced neurotoxicity in rat cortical cultures. J. Neurosci. Res..

[155] Sumiyoshi E., Matsuzaki K., Sugimoto N., Tanabe Y., Hara T., Katakura M., Miyamoto M., Mishima S., Shido O. (2019). Sub-chronic consumption of dark chocolate enhances cognitive function and releases nerve growth factors: A parallel-group randomized trial. Nutrients.

[156] Nehlig A. (2013). The neuroprotective effects of cocoa flavanol and its influence on cognitive performance. Br. J. Clin. Pharmacol..

[157] Marsh C.E., Carter H.H., Guelfi K.J., Smith K.J., Pike K.E., Naylor L.H., Green D.J. (2017). Brachial and cerebrovascular functions are enhanced in postmenopausal women after ingestion of chocolate with a high concentration of cocoa. J. Nutr..

[158] Occhiuto F., Palumbo D.R., Samperi S., Zangla G., Pino A., De Pasquale R., Circosta C. (2009). The isoflavones mixture from *Trifolium pratense* L. protects HCN 1-A neurons from oxidative stress. Phytother. Res..

[159] Zameer S., Najmi A.K., Vohora D., Akhtar M. (2018). A review on therapeutic potentials of *Trigonella foenum graecum* (fenugreek) and its chemical constituents in neurological disorders: Complementary roles to its hypolipidemic, hypoglycemic, and antioxidant potential. Nutr. Neurosci..

[160] Karcheva-Bahchevanska D., Lukova P. (2017). Therapeutic effects of anthocyannins from Vaccinium genus L.. Int. J. Med. Res. Pharm..

[161] Subash S., Essa M.M., Al-Adawi S., Memon M.A., Manivasagam T., Akbar M. (2014). Neuroprotective effects of berry fruits on neurodegenerative diseases. Neural Regen. Res..

[162] Nyakas C., Felszeghy K., Szabó R., Keijser J.N., Luiten P.G.M., Szombathelyi Z., Tihanyi K. (2009). Neuroprotective effects of vinpocetine and its major metabolite cis-apovincaminic acid on NMDA-induced neurotoxicity in a rat entorhinal cortex lesion model. CNS Neurosci. Ther..

[163] Kim J., Seo Y.H., Kim J., Goo N., Jeong Y., Bae H.J., Jung S.Y., Lee J., Ryu J.H. (2020). Casticin ameliorates scopolamine-induced cognitive dysfunction in mice. J. Ethnopharmacol..

[164] Kakkar S., Bais S. (2014). A review on protocatechuic Acid and its pharmacological potential. ISRN Pharmacol..

[165] Zahiruddin S., Basist P., Parveen A., Parveen R., Khan W., Ahmad S. (2020). Ashwagandha in brain disorders: A review of recent developments. J. Ethnopharmacol..

[166] Dar N.J. (2020). MuzamilAhmad Neurodegenerative diseases and *Withania somnifera* (L.): An update. J. Ethnopharmacol..

[167] Tandon N., Yadav S.S. (2020). Safety and clinical effectiveness of *Withania Somnifera* (Linn.) Dunal root in human ailments. J. Ethnopharmacol..

[168] Choudhary D., Bhattacharyya S., Bose S. (2017). Efficacy and Safety of Ashwagandha (*Withania somnifera* (L.) Dunal) Root Extract in Improving Memory and Cognitive Functions. J. Diet. Suppl..

[169] Gupta M., Kaur G. (2019). *Withania somnifera* (L.) Dunal ameliorates neurodegeneration and cognitive impairments associated with systemic inflammation. BMC Complement. Altern. Med..

[170] Saenghong N., Wattanathorn J., Muchimapura S., Tongun T., Piyavhatkul N., Banchonglikitkul C., Kajsongkram T. (2012). *Zingiber officinale* improves cognitive function of the middle-aged healthy women. Evid. Based Complement. Altern. Med..

[171] Wattanathorn J., Jittiwat J., Tongun T., Muchimapura S., Ingkaninan K. (2011). *Zingiber officinale* Mitigates Brain Damage and Improves Memory Impairment in Focal Cerebral Ischemic Rat. Evid. Based Complement. Altern. Med..

[172] Mao Q.-Q., Xu X.-Y., Cao S.-Y., Gan R.-Y., Corke H., Beta T., Li H.-B. (2019). Bioactive compounds and bioactivities of Ginger (*Zingiber officinale* Roscoe). Foods.

[173] Zhang F., Zhang J.-G., Yang W., Xu P., Xiao Y.-L., Zhang H.-T. (2018). 6-Gingerol attenuates LPS-induced neuroinflammation and cognitive impairment partially via suppressing astrocyte overactivation. Biomed. Pharmacother..

[174] Park E., Ryu M.J., Kim N.K., Bae M.H., Seo Y., Kim J., Yeo S., Kanwal M., Choi C.W., Heo J.Y. (2019). Synergistic neuroprotective effect of *Schisandra chinensis* and *Ribes fasciculatum* on neuronal cell death and scopolamine-induced cognitive impairment in rats. Int. J. Mol. Sci..

[175] McLennan S.N., Lam A.K., Mathias J.L., Koblar S.A., Hamilton-Bruce M.A., Jannes J. (2011). Role of vasodilation in cognitive impairment. Int. J. Stroke.

[176] Hitzenberger G., Sommer W., Grandt R. (1990). Influence of vinpocetine on warfarin-induced inhibition of coagulation. Int. J. Clin. Pharmacol. Ther. Toxicol..

[177] Wurglics M., Schubert-Zsilavecz M. (2006). *Hypericum perforatum*: A “modern” herbal antidepressant: Pharmacokinetics of active ingredients. Clin. Pharmacokinet..

[178] Sasaki K., El Omri A., Kondo S., Han J., Isoda H. (2013). *Rosmarinus officinalis* polyphenols produce anti-depressant like effect through monoaminergic and cholinergic functions modulation. Behav. Brain Res..

[179] Bak L.K., Schousboe A., Waagepetersen H.S. (2006). The glutamate/GABA-glutamine cycle: Aspects of transport, neurotransmitter homeostasis and ammonia transfer. J. Neurochem..

[180] Hajirahimkhan A., Dietz B.M., Bolton J.L. (2013). Botanical modulation of menopausal symptoms: Mechanisms of action?. Planta Med..

[181] Camfield D.A., Stough C., Farrimond J., Scholey A.B. (2014). Acute effects of tea constituents L-theanine, caffeine, and epigallocatechin gallate on cognitive function and mood: A systematic review and meta-analysis. Nutr. Rev..

[182] Liu R.H. (2004). Potential synergy of phytochemicals in cancer prevention: Mechanism of action. J. Nutr..

[183] Campos-Vega R., Oomah B.D., Tiwari B.K., Brunton N.P., Brennan C.S. (2013). Chemistry and classification of phytochemicals. Handbook of Plant Food Phytochemicals.

[184] Giada M.D.L.R., Morales-González J.A. (2013). Food Phenolic Compounds: Main Classes, Sources and Their Antioxidant Power. Oxidative Stress and Chronic Degenerative Diseases.

[185] Liu R.H. (2013). Health-promoting components of fruits and vegetables in the diet. Adv. Nutr..

[186] Verpoorte R., Worsfold P., Townshend A., Poole C. (2005). Alkaloids. Encyclopedia of Analytical Science.

[187] Debnath B., Singh W.S., Das M., Goswami S., Singh M.K., Maiti D., Manna K. (2018). Role of plant alkaloids on human health: A review of biological activities. Mater. Today Chem..

[188] Konrath E.L., Passos C.D.S., Klein L.C., Henriques A.T. (2013). Alkaloids as a source of potential anticholinesterase inhibitors for the treatment of Alzheimer’s disease. J. Pharm. Pharmacol..

[189] Brahmkshatriya P.P., Brahmkshatriya P.S., Ramawat K.G., Mérillon J.-M. (2013). Terpenes: Chemistry, Biological Role, and Therapeutic Applications. Natural Products: Phytochemistry, Botany and Metabolism of Alkaloids, Phenolics and Terpenes.

[190] Pearlson G. (2020). Chapter 9—Chemistry, chemical analysis, and extraction. Terpenes to tinctures. Weed Science.

[191] Agus H.H., Patel V.B., Preedy V.R. (2021). Chapter 4—Terpene toxicity and oxidative stress. Toxicology: Oxidative Stress and Dietary Antioxidants.

[192] Bell L., Lamport D.J., Butler L.T., Williams C.M. (2015). A review of the cognitive effects observed in humans following acute supplementation with flavonoids, and their associated mechanisms of action. Nutrients.

[193] Nouri Z., Fakhri S., El-Senduny F.F., Sanadgol N., Abd-Elghani G.E., Farzaei M.H., Chen J.T. (2019). On the neuroprotective effects of naringenin: Pharmacological targets, signaling pathways, molecular mechanisms, and clinical perspective. Biomolecules.

[194] Tutunchi H., Naeini F., Ostadrahimi A., Hosseinzadeh-Attar M.J. (2020). Naringenin, a flavanone with antiviral and anti-inflammatory effects: A promising treatment strategy against COVID-19. Phytother. Res..

[195] Umukoro S., Kalejaye H.A., Ben-Azu B., Ajayi A.M. (2018). Naringenin attenuates behavioral derangements induced by social defeat stress in mice via inhibition of acetylcholinesterase activity, oxidative stress and release of pro-inflammatory cytokines. Biomed. Pharmacother..

[196] Piironen V., Lampi A.-M., Poutanen K., Åman P. (2014). Chapter 9—Rye as a Source of Phytosterols, Tocopherols, and Tocotrienols. Rye and Health.

[197] Izzo A.A. (2012). Interactions between herbs and conventional drugs: Overview of the clinical data. Med. Princ. Pract..

[198] Ondieki G., Nyagblordzro M., Kikete S., Liang R., Wang L., He X. (2017). Cytochrome P450 and P-Glycoprotein-Mediated Interactions Involving African Herbs Indicated for Common Noncommunicable Diseases. Evid. Based Complement. Altern. Med..

[199] Zhou S., Gao Y., Jiang W., Huang M., Xu A., Paxton J.W. (2003). Interactions of herbs with cytochrome P450. Drug Metab. Rev..

[200] Cermak R. (2008). Effect of dietary flavonoids on pathways involved in drug metabolism. Expert Opin. Drug Metab. Toxicol..

[201] Liang Y., Li S., Chen L. (2015). The physiological role of drug transporters. Protein Cell.

[202] Grimstein M., Huang S.-M. (2018). A regulatory science viewpoint on botanical-drug interactions. J. Food Drug Anal..

[203] Roth M., Obaidat A., Hagenbuch B. (2012). OATPs, OATs and OCTs: The organic anion and cation transporters of the SLCO and SLC22A gene superfamilies. Br. J. Pharmacol..

[204] Zamek-Gliszczynski M.J., Taub M.E., Chothe P.P., Chu X., Giacomini K.M., Kim R.B., Ray A.S., Stocker S.L., Unadkat J.D., Wittwer M.B. (2018). Transporters in Drug Development: 2018 ITC Recommendations for Transporters of Emerging Clinical Importance. Clin. Pharmacol. Ther..

[205] Fitzpatrick F.A. (2004). Cyclooxygenase enzymes: Regulation and function. Curr. Pharm. Des..

[206] Churihar R., Solanki P., Vyas S., Hemant Tanwani H., Shubham Atal S. (2016). Analgesic activity of cinnamaldehyde *per se* and it’s interaction with diclofenac sodium and pentazocine in Swiss albino mice. Int. J. Phamacog..

[207] Finberg J.P.M., Rabey J.M. (2016). Inhibitors of MAo-A and MAo-B in Psychiatry and Neurology. Front. Pharmacol..

[208] Hritcu L., Ionita R., Postu P.A., Gupta G.K., Turkez H., Lima T.C., Carvalho C.U.S., de Sousa D.P. (2017). Antidepressant Flavonoids and Their Relationship with Oxidative Stress. Oxid. Med. Cell. Longev..

[209] Jewett B.E., Thapa B. (2020). Physiology, NMDA Receptor. StatPearls.

[210] Page A.T., Falster M.O., Litchfield M., Pearson S.-A., Etherton-Beer C. (2019). Polypharmacy among older Australians, 2006–2017: A population-based study. Med. J. Aust..

[211] National Center for Health Statistics (US) (2019). Health, United States, 2018.

[212] Nies A.T., Koepsell H., Damme K., Schwab M. (2011). Organic cation transporters (OCTs, MATEs), in vitro and in vivo evidence for the importance in drug therapy. Handbook of Experimental Pharmacology.

[213] Koepsell H. (2020). Organic Cation Transporters in Health and Disease. Pharmacol. Rev..

[214] Koepsell H. (2015). Role of organic cation transporters in drug-drug interaction. Expert Opin. Drug Metab. Toxicol..

[215] Benet L.Z., Cummins C.L., Wu C.Y. (2003). Transporter-enzyme interactions: Implications for predicting drug-drug interactions from in vitro data. Curr. Drug Metab..

[216] Liu X. (2019). Transporter-Mediated Drug-Drug Interactions and Their Significance. Adv. Exp. Med. Biol..

[217] Smolders E.J., de Kanter C.T.M.M., de Knegt R.J., van der Valk M., Drenth J.P.H., Burger D.M. (2016). Drug–Drug Interactions Between Direct-Acting Antivirals and Psychoactive Medications. Clin. Pharmacokinet..

[218] DrugBank Online. https://go.drugbank.com/.

[219] Zhou S.-F., Zhou Z.-W., Yang L.-P., Cai J.-P. (2009). Substrates, inducers, inhibitors and structure-activity relationships of human Cytochrome P450 2C9 and implications in drug development. Curr. Med. Chem..

[220] Vildhede A., Karlgren M., Svedberg E.K., Wisniewski J.R., Lai Y., Norén A., Artursson P. (2014). Hepatic uptake of atorvastatin: Influence of variability in transporter expression on uptake clearance and drug-drug interactions. Drug Metab. Dispos..

[221] Elsby R., Martin P., Surry D., Sharma P., Fenner K. (2016). Solitary Inhibition of the Breast Cancer Resistance Protein Efflux Transporter Results in a Clinically Significant Drug-Drug Interaction with Rosuvastatin by Causing up to a 2-Fold Increase in Statin Exposure. Drug Metab. Dispos..

[222] Shugarts S., Benet L.Z. (2009). The role of transporters in the pharmacokinetics of orally administered drugs. Pharm. Res..

[223] Clarke T.A., Waskell L.A. (2003). The metabolism of clopidogrel is catalyzed by human cytochrome P450 3A and is inhibited by atorvastatin. Drug Metab. Dispos..

[224] Kim S.-J., Yoshikado T., Ieiri I., Maeda K., Kimura M., Irie S., Kusuhara H., Sugiyama Y. (2016). Clarification of the Mechanism of Clopidogrel-Mediated Drug-Drug Interaction in a Clinical Cassette Small-dose Study and Its Prediction Based on In Vitro Information. Drug Metab. Dispos..

[225] Varma M.V.S., Bi Y.-A., Lazzaro S., West M. (2019). Clopidogrel as a Perpetrator of Drug-Drug Interactions: A Challenge for Quantitative Predictions?. Clin. Pharmacol. Ther..

[226] Lagas J.S., van der Kruijssen C.M.M., van de Wetering K., Beijnen J.H., Schinkel A.H. (2009). Transport of diclofenac by breast cancer resistance protein (ABCG2) and stimulation of multidrug resistance protein 2 (ABCC2)-mediated drug transport by diclofenac and benzbromarone. Drug Metab. Dispos..

[227] Kindla J., Müller F., Mieth M., Fromm M.F., König J. (2011). Influence of non-steroidal anti-inflammatory drugs on organic anion transporting polypeptide (OATP) 1B1- and OATP1B3-mediated drug transport. Drug Metab. Dispos..

[228] Müller F., Weitz D., Derdau V., Sandvoss M., Mertsch K., König J., Fromm M.F. (2017). Contribution of MATE1 to Renal Secretion of the NMDA Receptor Antagonist Memantine. Mol. Pharm..

[229] Williams E.I., Betterton R.D., Davis T.P., Ronaldson P.T. (2020). Transporter-Mediated Delivery of Small Molecule Drugs to the Brain: A Critical Mechanism That Can Advance Therapeutic Development for Ischemic Stroke. Pharmaceutics.

[230] Beconi M.G., Howland D., Park L., Lyons K., Giuliano J., Dominguez C., Munoz-Sanjuan I., Pacifici R. (2011). Pharmacokinetics of memantine in rats and mice. PLoS Curr..

[231] Li Y., Meng Q., Yang M., Liu D., Hou X., Tang L., Wang X., Lyu Y., Chen X., Liu K. (2019). Current trends in drug metabolism and pharmacokinetics. Acta Pharm. Sin. B.

[232] Lee J.O., Lee S.K., Kim J.H., Kim N., You G.Y., Moon J.W., Kim S.J., Park S.H., Kim H.S. (2012). Metformin regulates glucose transporter 4 (GLUT4) translocation through AMP-activated protein kinase (AMPK)-mediated Cbl/CAP signaling in 3T3-L1 preadipocyte cells. J. Biol. Chem..

[233] Kim J., Yang G., Kim Y., Kim J., Ha J. (2016). AMPK activators: Mechanisms of action and physiological activities. Exp. Mol. Med..

[234] Hemauer S.J., Patrikeeva S.L., Nanovskaya T.N., Hankins G.D.V., Ahmed M.S. (2010). Role of human placental apical membrane transporters in the efflux of glyburide, rosiglitazone, and metformin. Am. J. Obstet. Gynecol..

[235] Gong L., Goswami S., Giacomini K.M., Altman R.B., Klein T.E. (2012). Metformin pathways: Pharmacokinetics and pharmacodynamics. Pharmacogenet. Genom..

[236] Alsarrani A., Kaplita P.V. (2019). In Silico and in vitro evaluation of brain penetration properties of selected nootropic agents. Future Drug Discov..

[237] Masubuchi Y., Hosokawa S., Horie T., Suzuki T., Ohmori S., Kitada M., Narimatsu S. (1994). Cytochrome P450 isozymes involved in propranolol metabolism in human liver microsomes. The role of CYP2D6 as ring-hydroxylase and CYP1A2 as N-desisopropylase. Drug Metab. Dispos..

[238] Dudley A.J., Bleasby K., Brown C.D. (2000). The organic cation transporter OCT2 mediates the uptake of beta-adrenoceptor antagonists across the apical membrane of renal LLC-PK(1) cell monolayers. Br. J. Pharmacol..

[239] Steiner A., Walle T. (1992). Potent inhibition of MAO mediated propranolol metabolism by dimethyl sulfoxide in Hep G2 cells. Res. Commun. Chem. Pathol. Pharmacol..

[240] Goldberg M.R., Sciberras D., De Smet M., Lowry R., Tomasko L., Lee Y., Olah T.V., Zhao J., Vyas K.P., Halpin R. (2001). Influence of beta-adrenoceptor antagonists on the pharmacokinetics of rizatriptan, a 5-HT1B/1D agonist: Differential effects of propranolol, nadolol and metoprolol. Br. J. Clin. Pharmacol..

[241] Obach R.S., Cox L.M., Tremaine L.M. (2005). Sertraline is metabolized by multiple cytochrome P450 enzymes, monoamine oxidases, and glucuronyl transferases in human: An in vitro study. Drug Metab. Dispos..

[242] Nielsen C.U., Frølund S., Abdulhadi S., Sari H., Langthaler L., Nøhr M.K., Kall M.A., Brodin B., Holm R. (2013). Sertraline inhibits the transport of PAT1 substrates in vivo and in vitro. Br. J. Pharmacol..

[243] Feng S., Zheng L., Tang S., Gu J., Jiang X., Wang L. (2019). In-vitro and in situ assessment of the efflux of five antidepressants by breast cancer resistance protein. J. Pharm. Pharmacol..

[244] Ring B.J., Patterson B.E., Mitchell M.I., Vandenbranden M., Gillespie J., Bedding A.W., Jewell H., Payne C.D., Forgue S.T., Eckstein J. (2005). Effect of tadalafil on cytochrome P450 3A4-mediated clearance: Studies in vitro and in vivo. Clin. Pharmacol. Ther..

[245] Kopečná-Zapletalová M., Krasulová K., Anzenbacher P., Hodek P., Anzenbacherová E. (2017). Interaction of isoflavonoids with human liver microsomal cytochromes P450: Inhibition of CYP enzyme activities. Xenobiotica.

[246] Chao W.-W., Lin B.-F. (2011). Bioactivities of major constituents isolated from *Angelica sinensis* (Danggui). Chin. Med..

[247] Dewanjee S., Dua T.K., Bhattacharjee N., Das A., Gangopadhyay M., Khanra R., Joardar S., Riaz M., De Feo V., Zia-Ul-Haq M. (2017). Natural products as alternative choices for P-glycoprotein (P-gp) inhibition. Molecules.

[248] Wang X., Wolkoff A.W., Morris M.E. (2005). Flavonoids as a novel class of human organic anion-transporting polypeptide OATP1B1 (OATP-C) modulators. Drug Metab. Dispos..

[249] Šarić Mustapić D., Debeljak Ž., Maleš Ž., Bojić M. (2018). The Inhibitory Effect of Flavonoid Aglycones on the Metabolic Activity of CYP3A4 Enzyme. Molecules.

[250] Mandery K., Bujok K., Schmidt I., Keiser M., Siegmund W., Balk B., König J., Fromm M.F., Glaeser H. (2010). Influence of the flavonoids apigenin, kaempferol, and quercetin on the function of organic anion transporting polypeptides 1A2 and 2B1. Biochem. Pharmacol..

[251] Chan T., Li Z., Zheng J., Cheung F.S.G., Zhu L., Zhou F. (2013). Inhibitory effects of apigenin and kaempferol on the essential solute carrier transporters. World J. Gastrointest. Pharmacol. Ther..

[252] Saeed M., Kadioglu O., Khalid H., Sugimoto Y., Efferth T. (2015). Activity of the dietary flavonoid, apigenin, against multidrug-resistant tumor cells as determined by pharmacogenomics and molecular docking. J. Nutr. Biochem..

[253] Mandery K., Balk B., Bujok K., Schmidt I., Fromm M.F., Glaeser H. (2012). Inhibition of hepatic uptake transporters by flavonoids. Eur. J. Pharm. Sci..

[254] Meng X., Liao S., Wang X., Wang S., Zhao X., Jia P., Pei W., Zheng X., Zheng X. (2014). Reversing P-glycoprotein-mediated multidrug resistance in vitro by α-asarone and β-asarone, bioactive cis-trans isomers from *Acorus tatarinowii*. Biotechnol. Lett..

[255] Liu H.-J., Lai X., Xu Y., Miao J.-K., Li C., Liu J.-Y., Hua Y.-Y., Ma Q., Chen Q. (2017). α-Asarone Attenuates Cognitive Deficit in a Pilocarpine-Induced Status Epilepticus Rat Model via a Decrease in the Nuclear Factor-κB Activation and Reduction in Microglia Neuroinflammation. Front. Neurol..

[256] Cartus A.T., Schrenk D. (2020). Metabolism of carcinogenic alpha-asarone by human cytochrome P450 enzymes. Naunyn. Schmiedebergs. Arch. Pharmacol..

[257] Pandit S., Mukherjee P.K., Ponnusankar S., Venkatesh M., Srikanth N. (2011). Metabolism mediated interaction of α-asarone and *Acorus calamus* with CYP3A4 and CYP2D6. Fitoterapia.

[258] Rodríguez-Páez L., Juárez-Sanchez M., Antúnez-Solís J., Baeza I., Wong C. (2003). Alpha-asarone inhibits HMG-CoA reductase, lowers serum LDL-cholesterol levels and reduces biliary CSI in hypercholesterolemic rats. Phytomedicine.

[259] Chellian R., Pandy V., Mohamed Z. (2017). Pharmacology and toxicology of α- and β-Asarone: A review of preclinical evidence. Phytomedicine.

[260] Shin J.-W., Cheong Y.-J., Koo Y.-M., Kim S., Noh C.-K., Son Y.-H., Kang C., Sohn N.-W. (2014). α-Asarone Ameliorates Memory Deficit in Lipopolysaccharide-Treated Mice via Suppression of Pro-Inflammatory Cytokines and Microglial Activation. Biomol. Ther..

[261] Das B.K., Swamy A.V., Koti B.C., Gadad P.C. (2019). Experimental evidence for use of *Acorus calamus* (asarone) for cancer chemoprevention. Heliyon.

[262] Guo L., Cui Y., Hao K. (2018). Effects of glycyrrhizin on the pharmacokinetics of asiatic acid in rats and its potential mechanism. Pharm. Biol..

[263] Pan Y., Abd-Rashid B.A., Ismail Z., Ismail R., Mak J.W., Pook P.C.K., Er H.M., Ong C.E. (2010). In vitro modulatory effects on three major human cytochrome P450 enzymes by multiple active constituents and extracts of *Centella asiatica*. J. Ethnopharmacol..

[264] Wright K.M., Magana A.A., Laethem R.M., Moseley C.L., Banks T.T., Maier C.S., Stevens J.F., Quinn J.F., Soumyanath A. (2020). *Centella asiatica* Water Extract Shows Low Potential for Cytochrome P450-Mediated Drug Interactions. Drug Metab. Dispos..

[265] Cheng Q., Liao M., Hu H., Li H., Wu L. (2018). Asiatic Acid (AA) Sensitizes Multidrug-Resistant Human Lung Adenocarcinoma A549/DDP Cells to Cisplatin (DDP) via Downregulation of P-Glycoprotein (MDR1) and Its Targets. Cell. Physiol. Biochem..

[266] Zhang Q., Cao Y.F., Ran R.X., Li R.S., Wu X., Dong P.P., Zhang Y.Y., Hu C.M., Wang W.M. (2016). Strong Specific Inhibition of UDP-glucuronosyltransferase 2B7 by Atractylenolide I and III. Phytother. Res..

[267] Ahmed S., Gul S., Gul H., Bangash M.H. (2013). Anti-inflammatory and anti-platelet activities of Avena sativa are mediated through the inhibition of cyclooxygenase and lipoxygenase enzymes. IJEHSR.

[268] Scarpa E.S., Mari M., Antonini E., Palma F., Ninfali P. (2018). Natural and synthetic avenanthramides activate caspases 2, 8, 3 and downregulate hTERT, MDR1 and COX-2 genes in CaCo-2 and Hep3B cancer cells. Food Funct..

[269] Ramasamy V.S., Samidurai M., Park H.J., Wang M., Park R.Y., Yu S.Y., Kang H.K., Hong S., Choi W.-S., Lee Y.Y. (2020). Avenanthramide-C Restores Impaired Plasticity and Cognition in Alzheimer’s Disease Model Mice. Mol. Neurobiol..

[270] Ramasamy S., Kiew L.V., Chung L.Y. (2014). Inhibition of human cytochrome P450 enzymes by Bacopa monnieri standardized extract and constituents. Molecules.

[271] Li H., Liu L., Xie L., Gan D., Jiang X. (2016). Effects of berberine on the pharmacokinetics of losartan and its metabolite EXP3174 in rats and its mechanism. Pharm. Biol..

[272] Han Y.-L., Yu H.-L., Li D., Meng X.-L., Zhou Z.-Y., Yu Q., Zhang X.-Y., Wang F.-J., Guo C. (2011). In vitro inhibition of Huanglian [*Rhizoma coptidis* (L.)] and its six active alkaloids on six cytochrome P450 isoforms in human liver microsomes. Phytother. Res..

[273] Zhang X., Qiu F., Jiang J., Gao C., Tan Y. (2011). Intestinal absorption mechanisms of berberine, palmatine, jateorhizine, and coptisine: Involvement of P-glycoprotein. Xenobiotica.

[274] Yu C.-P., Huang C.-Y., Lin S.-P., Hou Y.-C. (2018). Activation of P-glycoprotein and CYP 3A by Coptidis Rhizoma in vivo: Using cyclosporine as a probe substrate in rats. J. Food Drug Anal..

[275] Min Y.D., Yang M.C., Lee K.H., Kim K.R., Choi S.U., Lee K.R. (2006). Protoberberine alkaloids and their reversal activity of P-gp expressed multidrug resistance (MDR) from the rhizome of *Coptis japonica* Makino. Arch. Pharm. Res..

[276] Tan K.W., Li Y., Paxton J.W., Birch N.P., Scheepens A. (2013). Identification of novel dietary phytochemicals inhibiting the efflux transporter breast cancer resistance protein (BCRP/ABCG2). Food Chem..

[277] Chen C., Wu Z.-T., Ma L.-L., Ni X., Lin Y.-F., Wang L., Chen K.-P., Huang C.-G., Pan G. (2015). Organic anion-transporting polypeptides contribute to the hepatic uptake of berberine. Xenobiotica.

[278] Obach R.S. (2000). Inhibition of human cytochrome P450 enzymes by constituents of St. John’s Wort, an herbal preparation used in the treatment of depression. J. Pharmacol. Exp. Ther..

[279] Gutmann H., Bruggisser R., Schaffner W., Bogman K., Botomino A., Drewe J. (2002). Transport of amentoflavone across the blood-brain barrier *in vitro*. Planta Med..

[280] Umegaki K., Taki Y., Endoh K., Taku K., Tanabe H., Shinozuka K., Sugiyama T. (2007). Bilobalide in *Ginkgo biloba* extract is a major substance inducing hepatic CYPs. J. Pharm. Pharmacol..

[281] Yaro P., Nie J., Xu M., Zeng K., He H., Yao J., Wang R., Zeng S. (2019). Influence of organic anion transporter 1/3 on the pharmacokinetics and renal excretion of ginkgolides and bilobalide. J. Ethnopharmacol..

[282] Weichel O., Hilgert M., Chatterjee S.S., Lehr M., Klein J. (1999). Bilobalide, a constituent of *Ginkgo biloba*, inhibits NMDA-induced phospholipase A2 activation and phospholipid breakdown in rat hippocampus. Naunyn. Schmiedebergs. Arch. Pharmacol..

[283] Srinivas N.R. (2015). Biochanin A: Understanding the complexities in the paradoxical drug-drug interaction potential. Eur. J. Drug Metab. Pharmacokinet..

[284] Zhang S., Morris M.E. (2003). Effects of the flavonoids biochanin A, morin, phloretin, and silymarin on P-glycoprotein-mediated transport. J. Pharmacol. Exp. Ther..

[285] Bircsak K.M., Aleksunes L.M. (2015). Interaction of Isoflavones with the BCRP/ABCG2 Drug Transporter. Curr. Drug Metab..

[286] Sissung T.M., Baum C.E., Kirkland C.T., Gao R., Gardner E.R., Figg W.D. (2010). Pharmacogenetics of membrane transporters: An update on current approaches. Mol. Biotechnol..

[287] An G., Morris M.E. (2011). The sulfated conjugate of biochanin A is a substrate of breast cancer resistant protein (ABCG2). Biopharm. Drug Dispos..

[288] Armutcu F., Akyol S., Ustunsoy S., Turan F.F. (2015). Therapeutic potential of caffeic acid phenethyl ester and its anti-inflammatory and immunomodulatory effects (Review). Exp. Ther. Med..

[289] Rastogi H., Jana S. (2014). Evaluation of Inhibitory Effects of Caffeic acid and Quercetin on Human Liver Cytochrome P450 Activities. Phytother. Res..

[290] Teng Y.-N., Wang C.C.N., Liao W.-C., Lan Y.-H., Hung C.-C. (2020). Caffeic Acid Attenuates Multi-Drug Resistance in Cancer Cells by Inhibiting Efflux Function of Human P-glycoprotein. Molecules.

[291] Hong Y.-J., Yang S.-Y., Nam M.-H., Koo Y.-C., Lee K.-W. (2015). Caffeic acid inhibits the uptake of 2-amino-1-methyl-6-phenylimidazo[4,5-b]pyridine (PhIP) by inducing the efflux transporters expression in Caco-2 cells. Biol. Pharm. Bull..

[292] Mora F., Molina J.D., Zubillaga E., López-Muñoz F., Álamo C. (2015). CYP450 and Its Implications in the Clinical Use of Antipsychotic Drugs. Clin. Exp. Pharmacol..

[293] Ding R., Shi J., Pabon K., Scotto K.W. (2012). Xanthines Down-Regulate the Drug Transporter ABCG2 and Reverse Multidrug Resistance. Mol. Pharmacol..

[294] Cao R., Peng W., Wang Z., Xu A. (2007). beta-Carboline alkaloids: Biochemical and pharmacological functions. Curr. Med. Chem..

[295] Passos C.D.S., Simoes-Pires C., Henriques A., Cuendet M., Carrupt P.-A., Christen P., Rahman A.U. (2014). Chapter 4—Alkaloids as Inhibitors of Monoamine Oxidases and Their Role in the Central Nervous System. Studies in Natural Products Chemistry.

[296] Ma Y., Wink M. (2010). The beta-carboline alkaloid harmine inhibits BCRP and can reverse resistance to the anticancer drugs mitoxantrone and camptothecin in breast cancer cells. Phytother. Res..

[297] Zhao T., He Y.-Q., Wang J., Ding K.-M., Wang C.-H., Wang Z.-T. (2011). Inhibition of human cytochrome P450 enzymes 3A4 and 2D6 by β-carboline alkaloids, harmine derivatives. Phytother. Res..

[298] Mohos V., Bencsik T., Boda G., Fliszár-Nyúl E., Lemli B., Kunsági-Máté S., Poór M. (2018). Interactions of casticin, ipriflavone, and resveratrol with serum albumin and their inhibitory effects on CYP2C9 and CYP3A4 enzymes. Biomed. Pharmacother..

[299] Liu C., Chen K., Lu Y., Fang Z., Yu G. (2018). Catalpol provides a protective effect on fibrillary Aβ1-42 -induced barrier disruption in an in vitro model of the blood-brain barrier. Phytother. Res..

[300] Bao Q., Shen X., Qian L., Gong C., Nie M., Dong Y. (2016). Anti-diabetic activities of catalpol in db/db mice. Korean J. Physiol. Pharmacol..

[301] Liu L., Cao X., Li T., Li X. (2019). Effects of catalpol on the activity of human liver cytochrome P450 enzymes. Xenobiotica.

[302] Knop J., Misaka S., Singer K., Hoier E., Müller F., Glaeser H., König J., Fromm M.F. (2015). Inhibitory Effects of Green Tea and (-)-Epigallocatechin Gallate on Transport by OATP1B1, OATP1B3, OCT1, OCT2, MATE1, MATE2-K and P-Glycoprotein. PLoS ONE.

[303] Satoh T., Fujisawa H., Nakamura A., Takahashi N., Watanabe K. (2016). Inhibitory effects of eight green tea catechins on cytochrome P450 1A2, 2C9, 2D6, and 3A4 activities. J. Pharm. Pharm. Sci..

[304] Reddy D.B., Reddy T.C.M., Jyotsna G., Sharan S., Priya N., Lakshmipathi V., Reddanna P. (2009). Chebulagic acid, a COX-LOX dual inhibitor isolated from the fruits of *Terminalia chebula* Retz., induces apoptosis in COLo-205 cell line. J. Ethnopharmacol..

[305] Achari C., Reddy G.V., Reddy T.C.M., Reddanna P. (2011). Chebulagic Acid Synergizes the Cytotoxicity of Doxorubicin in Human Hepatocellular Carcinoma Through COX-2 Dependant Modulation of MDR-1. Med. Chem..

[306] Clifford M.N., Kerimi A., Williamson G. (2020). Bioavailability and metabolism of chlorogenic acids (acyl-quinic acids) in humans. Compr. Rev. Food Sci. Food Saf..

[307] Najar I.A., Sachin B.S., Sharma S.C., Satti N.K., Suri K.A., Johri R.K. (2010). Modulation of P-glycoprotein ATPase activity by some phytoconstituents. Phytother. Res..

[308] Zhang L., Fan Y., Su H., Wu L., Huang Y., Zhao L., Han B., Shu G., Xiang M., Yang J.-M. (2018). Chlorogenic acid methyl ester exerts strong anti-inflammatory effects via inhibiting the COX-2/NLRP3/NF-κB pathway. Food Funct..

[309] Pang C., Sheng Y.-C., Jiang P., Wei H., Ji L.-L. (2015). Chlorogenic acid prevents acetaminophen-induced liver injury: The involvement of CYP450 metabolic enzymes and some antioxidant signals. J. Zhejiang Univ. Sci. B.

[310] Meng S., Cao J., Feng Q., Peng J., Hu Y. (2013). Roles of chlorogenic acid on regulating glucose and lipids metabolism: A review. Evid. Based Complement. Altern. Med..

[311] Mei Y., Pan D., Jiang Y., Zhang W., Yao X., Dai Y., Yu Y., Yao X. (2019). Target discovery of chlorogenic acid derivatives from the flower buds of Lonicera macranthoides and their MAO B inhibitory mechanism. Fitoterapia.

[312] Miyazawa M., Shindo M., Shimada T. (2001). Oxidation of 1,8-cineole, the monoterpene cyclic ether originated from eucalyptus polybractea, by cytochrome P450 3A enzymes in rat and human liver microsomes. Drug Metab. Dispos..

[313] Xi J., Yun M., Lee D., Park M.-N., Kim E.-O., Sohn E.J., Kwon B.-M. (2015). Cinnamaldehyde Derivative (CB-PIC) Sensitizes Chemo-Resistant Cancer Cells to Drug-Induced Apoptosis via Suppression of MDR1 and its Upstream STAT3 and AKT Signalling. Cell. Physiol. Biochem..

[314] Huang B., Yuan H.D., Kim D.Y., Quan H.Y., Chung S.H. (2011). Cinnamaldehyde prevents adipocyte differentiation and adipogenesis via regulation of peroxisome proliferator-activated receptor-γ (PPARγ) and AMP-activated protein kinase (AMPK) pathways. J. Agric. Food Chem..

[315] Guo J.-Y., Huo H.-R., Zhao B.-S., Liu H.-B., Li L.-F., Ma Y.-Y., Guo S.-Y., Jiang T.-L. (2006). Cinnamaldehyde reduces IL-1beta-induced cyclooxygenase-2 activity in rat cerebral microvascular endothelial cells. Eur. J. Pharmacol..

[316] Hasegawa A., Yoshino M., Nakamura H., Ishii I., Watanabe T., Kiuchi M., Ishikawa T., Ohmori S., Kitada M. (2002). Identification of inhibitory component in cinnamon--o-methoxycinnamaldehyde inhibits CYP1A2 and CYP2E1-. Drug Metab. Pharmacokinet..

[317] Williams C.H., Lawson J., Backwell F.R. (1992). Inhibition and inactivation of monoamine oxidase by 3-amino-1-phenyl-prop-1-enes. Biochim. Biophys. Acta.

[318] Chen C., Wu C., Lu X., Yan Z., Gao J., Zhao H., Li S. (2013). Coniferyl ferulate, a strong inhibitor of glutathione s-transferase isolated from radix *Angelicae sinensis*, reverses multidrug resistance and downregulates P-glycoprotein. Evid. Based Complement. Altern. Med..

[319] Ro J.S., Lee S.S., Lee K.S., Lee M.K. (2001). Inhibition of type A monoamine oxidase by coptisine in mouse brain. Life Sci..

[320] Li L., Sun S., Weng Y., Song F., Zhou S., Bai M., Zhou H., Zeng S., Jiang H. (2016). Interaction of six protoberberine alkaloids with human organic cation transporters 1, 2 and 3. Xenobiotica.

[321] Yoon S.-A., Kang S.-I., Shin H.-S., Kang S.-W., Kim J.-H., Ko H.-C., Kim S.-J. (2013). p-Coumaric acid modulates glucose and lipid metabolism via AMP-activated protein kinase in L6 skeletal muscle cells. Biochem. Biophys. Res. Commun..

[322] Luceri C., Guglielmi F., Lodovici M., Giannini L., Messerini L., Dolara P. (2004). Plant phenolic 4-coumaric acid protects against intestinal inflammation in rats. Scand. J. Gastroenterol..

[323] Yang F., Wang Y., Li G., Xue J., Chen Z.-L., Jin F., Luo L., Zhou X., Ma Q., Cai X. (2018). Effects of corilagin on alleviating cholestasis via farnesoid X receptor-associated pathways in vitro and *in vivo*. Br. J. Pharmacol..

[324] Lautenschläger M., Sendker J., Hüwel S., Galla H.J., Brandt S., Düfer M., Riehemann K., Hensel A. (2015). Intestinal formation of trans-crocetin from saffron extract (*Crocus sativus* L.) and in vitro permeation through intestinal and blood brain barrier. Phytomedicine.

[325] Neyshaburinezhad N., Hashemi M., Ramezani M., Arabzadeh S., Behravan J., Kalalinia F. (2018). The effects of crocetin, extracted from saffron, in chemotherapy against the incidence of multiple drug resistance phenotype. Iran. J. Basic Med. Sci..

[326] Berger F., Hensel A., Nieber K. (2010). Trans-crocetin is involved in the inhibition of the glutamatergic synaptic transmission on rat cortical neurones by saffron extract. Planta Med..

[327] Kamyar M., Razavi B.M., Hasani F.V., Mehri S., Foroutanfar A., Hosseinzadeh H. (2016). Crocin prevents haloperidol-induced orofacial dyskinesia: Possible an antioxidant mechanism. Iran. J. Basic Med. Sci..

[328] Dovrtělová G., Nosková K., Juřica J., Turjap M., Zendulka O. (2015). Can bioactive compounds of *Crocus sativus* L. Influence the metabolic activity of selected CYP enzymes in the rat?. Physiol. Res..

[329] Razavi S.M.S., Vaziri R.M., Karimi G., Arabzadeh S., Keyvani V., Behravan J., Kalalinia F. (2020). Crocin Increases Gastric Cancer Cells’ Sensitivity to Doxorubicin. Asian Pac. J. Cancer Prev..

[330] Lopes-Rodrigues V., Sousa E., Vasconcelos M.H. (2016). Curcumin as a Modulator of P-Glycoprotein in Cancer: Challenges and Perspectives. Pharmaceuticals.

[331] Wortelboer H.M., Usta M., van der Velde A.E., Boersma M.G., Spenkelink B., van Zanden J.J., Rietjens I.M.C.M., van Bladeren P.J., Cnubben N.H.P. (2003). Interplay between MRP inhibition and metabolism of MRP inhibitors: The case of curcumin. Chem. Res. Toxicol..

[332] Zhou X., Zhang F., Chen C., Guo Z., Liu J., Yu J., Xu Y., Zhong D., Jiang H. (2017). Impact of curcumin on the pharmacokinetics of rosuvastatin in rats and dogs based on the conjugated metabolites. Xenobiotica.

[333] Li H., Krstin S., Wink M. (2018). Modulation of multidrug resistant in cancer cells by EGCG, tannic acid and curcumin. Phytomedicine.

[334] Naganuma M., Saruwatari A., Okamura S., Tamura H. (2006). Turmeric and curcumin modulate the conjugation of 1-naphthol in Caco-2 cells. Biol. Pharm. Bull..

[335] Basu N.K., Kole L., Kubota S., Owens I.S. (2004). Human UDP-glucuronosyltransferases show atypical metabolism of mycophenolic acid and inhibition by curcumin. Drug Metab. Dispos..

[336] Volak L.P., Court M.H. (2010). Role for protein kinase C delta in the functional activity of human UGT1A6: Implications for drug-drug interactions between PKC inhibitors and UGT1A6. Xenobiotica.

[337] Ricci J.W., Lovato D., Larson R.S. (2015). ABCG2 Inhibitors: Will They Find Clinical Relevance?. J. Develop. Drugs.

[338] Gameiro M., Silva R., Rocha-Pereira C., Carmo H., Carvalho F., Bastos M.D.L., Remião F. (2017). Cellular Models and In Vitro Assays for the Screening of modulators of P-gp, MRP1 and BCRP. Molecules.

[339] Sun X., Li J., Guo C., Xing H., Xu J., Wen Y., Qiu Z., Zhang Q., Zheng Y., Chen X. (2016). Pharmacokinetic effects of curcumin on docetaxel mediated by OATP1B1, OATP1B3 and CYP450s. Drug Metab. Pharmacokinet..

[340] Zapletalova M.K., Lecianova A., Spicakova A., Anzenbacherova E., Anzenbacher P. (2013). Interaction of anthocyanins with human liver microsomal cytochromes P450. Biomed. Pap. Med. Fac. Univ. Palacky Olomouc Czech. Repub..

[341] Dreiseitel A., Oosterhuis B., Vukman K.V., Schreier P., Oehme A., Locher S., Hajak G., Sand P.G. (2009). Berry anthocyanins and anthocyanidins exhibit distinct affinities for the efflux transporters BCRP and MDR1. Br. J. Pharmacol..

[342] Eriksson A.H., Rønsted N., Güler S., Jäger A.K., Sendra J.R., Brodin B. (2012). In-vitro evaluation of the P-glycoprotein interactions of a series of potentially CNS-active Amaryllidaceae alkaloids. J. Pharm. Pharmacol..

[343] Wang Q., Xia M., Liu C., Guo H., Ye Q., Hu Y., Zhang Y., Hou M., Zhu H., Ma J. (2008). Cyanidin-3-*O*-beta-glucoside inhibits iNOS and COX-2 expression by inducing liver X receptor alpha activation in THP-1 macrophages. Life Sci..

[344] Srovnalova A., Svecarova M., Zapletalova M.K., Anzenbacher P., Bachleda P., Anzenbacherova E., Dvorak Z. (2014). Effects of anthocyanidins and anthocyanins on the expression and catalytic activities of CYP2A6, CYP2B6, CYP2C9, and CYP3A4 in primary human hepatocytes and human liver microsomes. J. Agric. Food Chem..

[345] Fuchikami H., Satoh H., Tsujimoto M., Ohdo S., Ohtani H., Sawada Y. (2006). Effects of herbal extracts on the function of human organic anion-transporting polypeptide OATP-B. Drug Metab. Dispos..

[346] Atherton K.M., Mutch E., Ford D. (2006). Metabolism of the soyabean isoflavone daidzein by CYP1A2 and the extra-hepatic CYPs 1A1 and 1B1 affects biological activity. Biochem. Pharmacol..

[347] Peng W.X., Wang L.S., Li H.D., Abd El-Aty A.M., Chen G.L., Zhou H.H. (2003). Evidence for the involvement of human liver microsomes CYP1A2 in the mono-hydroxylation of daidzein. Clin. Chim. Acta.

[348] Okura T., Ibe M., Umegaki K., Shinozuka K., Yamada S. (2010). Effects of dietary ingredients on function and expression of P-glycoprotein in human intestinal epithelial cells. Biol. Pharm. Bull..

[349] Pritchett L.E., Atherton K.M., Mutch E., Ford D. (2008). Glucuronidation of the soyabean isoflavones genistein and daidzein by human liver is related to levels of UGT1A1 and UGT1A9 activity and alters isoflavone response in the MCF-7 human breast cancer cell line. J. Nutr. Biochem..

[350] Abd El-Aty A.M., Shah S.S., Kim B.M., Choi J.H., Cho H.J., Chang B.J., Shin H.C., Lee K.B., Shimoda M., Shime J.-H. (2008). In Vitro inhibitory potential of decursin and decursinol angelate on the catalytic activity of cytochrome P-450 1A1/2, 2D15, and 3A12 isoforms in canine hepatic microsomes. Arch. Pharm. Res..

[351] Abdallah H.M., Al-Abd A.M., El-Dine R.S., El-Halawany A.M. (2015). P-glycoprotein inhibitors of natural origin as potential tumor chemo-sensitizers: A review. J. Adv. Res..

[352] Zhang J., Li L., Tang S., Hale T.W., Xing C., Jiang C., Lü J. (2015). Cytochrome P450 Isoforms in the Metabolism of Decursin and Decursinol Angelate from Korean Angelica. Am. J. Chin. Med..

[353] Riha J., Brenner S., Srovnalova A., Klameth L., Dvorak Z., Jäger W., Thalhammer T. (2015). Effects of anthocyanins on the expression of organic anion transporting polypeptides (SLCOs/OATPs) in primary human hepatocytes. Food Funct..

[354] Liagre B., Vergne-Salle P., Corbiere C., Charissoux J.L., Beneytout J.L. (2004). Diosgenin, a plant steroid, induces apoptosis in human rheumatoid arthritis synoviocytes with cyclooxygenase-2 overexpression. Arthritis Res. Ther..

[355] Manda V.K., Avula B., Wong Y.H., Smillie T.J., Kahn I.A., Khan S.I. (2013). Characterization of in Vitro ADME Properties of Diosgenin and Dioscin from *Dioscorea villosa*. Planta Med..

[356] Vijayakumar T.M., Ilango K., Kumar R.M., Agrawal A., Dubey G.P. (2015). Effect of *Dioscorea bulbifera* and its Major Bioactive Compound, Diosgenin on CYP450 Mediated Drug Metabolism. JBAPN.

[357] González-Sarrías A., Azorín-Ortuño M., Yáñez-Gascón M.J., Tomás-Barberán F.A., García-Conesa M.T., Espín J.C. (2009). Dissimilar in vitro and in vivo effects of ellagic acid and its microbiota-derived metabolites, urolithins, on the cytochrome P450 1A1. J. Agric. Food Chem..

[358] Barch D.H., Rundhaugen L.M., Thomas P.E., Kardos P., Pillay N.S. (1994). Dietary ellagic acid inhibits the enzymatic activity of CYP1A1 without altering hepatic concentrations of CYP1A1 or CYP1A1 mRNA. Biochem. Biophys. Res. Commun..

[359] Celik G., Semiz A., Karakurt S., Arslan S., Adali O., Sen A. (2013). A comparative study for the evaluation of two doses of ellagic acid on hepatic drug metabolizing and antioxidant enzymes in the rat. Biomed Res. Int..

[360] Whitley A.C., Sweet D.H., Walle T. (2005). The dietary polyphenol ellagic acid is a potent inhibitor of hOAT1. Drug Metab. Dispos..

[361] Van Wert A.L., Gionfriddo M.R., Sweet D.H. (2010). Organic anion transporters: Discovery, pharmacology, regulation and roles in pathophysiology. Biopharm. Drug Dispos..

[362] Ahn D., Putt D., Kresty L., Stoner G.D., Fromm D., Hollenberg P.F. (1996). The effects of dietary ellagic acid on rat hepatic and esophageal mucosal cytochromes P450 and phase II enzymes. Carcinogenesis.

[363] Tamaki N., Matsunami K., Otsuka H., Shinzato T., Aramoto M., Takeda Y. (2008). Rearranged ent-kauranes from the stems of Tricalysia dubia and their biological activities. J. Nat. Med..

[364] Sul Y.H., Lee M.S., Cha E.Y., Thuong P.T., Khoi N.M., Song I.S. (2013). An ent-kaurane diterpenoid from Croton tonkinensis induces apoptosis by regulating AMP-activated protein kinase in SK-HEP1 human hepatocellular carcinoma cells. Biol. Pharm. Bull..

[365] Lee M., Kim S.H., Lee H.K., Cho Y., Kang J., Sung S.H. (2014). Ent-kaurane and ent-pimarane diterpenes from Siegesbeckia pubescens inhibit lipopolysaccharide-induced nitric oxide production in BV2 microglia. Biol. Pharm. Bull..

[366] Chiba T., Sato Y., Suzuki S., Umegaki K. (2015). Concomitant use of dietary supplements and medicines in patients due to miscommunication with physicians in Japan. Nutrients.

[367] Roth M., Timmermann B.N., Hagenbuch B. (2011). Interactions of green tea catechins with organic anion-transporting polypeptides. Drug Metab. Dispos..

[368] Jodoin J., Demeule M., Beliveau R. (2002). Inhibition of the multidrug resistance P-glycoprotein activity by green tea polyphenols. Biochim. Biophys. Acta.

[369] Greenmol BD | Nature Derived Molecule Database. https://www.greenmolbd.gov.bd/search?search_in=target&term=Solute%20carrier%20organic%20anion%20transporter%20family%20member%201B1%20(OATP1B1.

[370] Wen Y., Zhao R., Gupta P., Fan Y., Zhang Y., Huang Z., Li X., Su Y., Liao L., Xie Y.-A. (2019). The epigallocatechin gallate derivative Y6 reverses drug resistance mediated by the ABCB1 transporter both in vitro and in vivo. Acta Pharm. Sin. B.

[371] Muto S., Fujita K., Yamazaki Y., Kamataki T. (2001). Inhibition by green tea catechins of metabolic activation of procarcinogens by human cytochrome P450. Mutat. Res..

[372] Isaka S.M., Awabe K.K., Noue S.O., Erba J.P.W., Iroli M.G., Amaki S.T., An T.K., Imura J.K., Atanabe H.W., Amada S.Y. (2013). Effects of Green Tea Catechins on Cytochrome P450 2B6, 2C8, 2C19, 2D6 and 3A Activities in Human Liver and Intestinal Microsomes. Drug Metab. Pharmacokinet..

[373] Kitagawa S., Nabekura T., Kamiyama S. (2004). Inhibition of P-glycoprotein function by tea catechins in KB-C2 cells. J. Pharm. Pharmacol..

[374] Alvarez A.I., Real R., Pérez M., Mendoza G., Prieto J.G., Merino G. (2010). Modulation of the activity of ABC transporters (P-glycoprotein, MRP2, BCRP) by flavonoids and drug response. J. Pharm. Sci..

[375] Wang E.J., Barecki-Roach M., Johnson W.W. (2002). Elevation of P-glycoprotein function by a catechin in green tea. Biochem. Biophys. Res. Commun..

[376] Farabegoli F., Papi A., Bartolini G., Ostan R., Orlandi M. (2010). (-)-Epigallocatechin-3-gallate downregulates Pg-P and BCRP in a tamoxifen resistant MCF-7 cell line. Phytomedicine.

[377] Iwano H., Ujita W., Nishikawa M., Ishii S., Inoue H., Yokota H. (2014). Effect of dietary eugenol on xenobiotic metabolism and mediation of UDP-glucuronosyltransferase and cytochrome P450 1A1 expression in rat liver. Int. J. Food Sci. Nutr..

[378] Jeong K.J., Kim D.Y., Quan H.-Y., Jo H.K., Kim G.W., Chung S.H. (2014). Effects of eugenol on hepatic glucose production and AMPK signaling pathway in hepatocytes and C57BL/6J mice. Fitoterapia.

[379] Kim S.S., Oh O.-J., Min H.-Y., Park E.-J., Kim Y., Park H.J., Nam Han Y., Lee S.K. (2003). Eugenol suppresses cyclooxygenase-2 expression in lipopolysaccharide-stimulated mouse macrophage RAW264.7 cells. Life Sci..

[380] Gardner I., Wakazono H., Bergin P., de Waziers I., Beaune P., Kenna J.G., Caldwell J. (1997). Cytochrome P450 mediated bioactivation of methyleugenol to 1’-hydroxymethyleugenol in Fischer 344 rat and human liver microsomes. Carcinogenesis.

[381] Harb A.A., Bustanji Y.K., Almasri I.M., Abdalla S.S. (2019). Eugenol Reduces LDL Cholesterol and Hepatic Steatosis in Hypercholesterolemic Rats by Modulating TRPV1 Receptor. Sci. Rep..

[382] Wie M.B., Won M.H., Lee K.H., Shin J.H., Lee J.C., Suh H.W., Song D.K., Kim Y.H. (1997). Eugenol protects neuronal cells from excitotoxic and oxidative injury in primary cortical cultures. Neurosci. Lett..

[383] Nabekura T., Kawasaki T., Furuta M., Kaneko T., Uwai Y. (2018). Effects of Natural Polyphenols on the Expression of Drug Efflux Transporter P-Glycoprotein in Human Intestinal Cells. ACS Omega.

[384] Yu L., Zhang Y., Ma R., Bao L., Fang J., Yu T. (2006). Potent protection of ferulic acid against excitotoxic effects of maternal intragastric administration of monosodium glutamate at a late stage of pregnancy on developing mouse fetal brain. Eur. Neuropsychopharmacol..

[385] Muthusamy G., Balupillai A., Ramasamy K., Shanmugam M., Gunaseelan S., Mary B., Prasad N.R. (2016). Ferulic acid reverses ABCB1-mediated paclitaxel resistance in MDR cell lines. Eur. J. Pharmacol..

[386] Zhuang X.-M., Chen L., Tan Y., Yang H.-Y., Lu C., Gao Y., Li H. (2017). Identification of human cytochrome P450 and UGT enzymes involved in the metabolism of ferulic acid, a major bioactive component in traditional Chinese medicines. Chin. J. Nat. Med..

[387] Mathew B., Baek S.C., Parambi D.G.T., Lee J.P., Mathew G.E., Jayanthi S., Vinod D., Rapheal C., Devikrishna V., Kondarath S.S. (2019). Potent and highly selective dual-targeting monoamine oxidase-B inhibitors: Fluorinated chalcones of morpholine versus imidazole. Archiv der Pharmazie.

[388] Wang E.J., Casciano C.N., Clement R.P., Johnson W.W. (2001). Active transport of fluorescent P-glycoprotein substrates: Evaluation as markers and interaction with inhibitors. Biochem. Biophys. Res. Commun..

[389] Virgona N., Yokotani K., Yamazaki Y., Shimura F., Chiba T., Taki Y., Yamada S., Shinozuka K., Murata M., Umegaki K. (2012). *Coleus forskohlii* extract induces hepatic cytochrome P450 enzymes in mice. Food Chem. Toxicol..

[390] Yokotani K., Chiba T., Sato Y., Taki Y., Yamada S., Shinozuka K., Murata M., Umegaki K. (2012). Hepatic cytochrome P450 mediates interaction between warfarin and *Coleus forskohlii* extract in vivo and in vitro. J. Pharm. Pharmacol..

[391] Hurley R.L., Barré L.K., Wood S.D., Anderson K.A., Kemp B.E., Means A.R., Witters L.A. (2006). Regulation of AMP-activated protein kinase by multisite phosphorylation in response to agents that elevate cellular cAMP. J. Biol. Chem..

[392] Egawa M., Kamata H., Kushiyama A., Sakoda H., Fujishiro M., Horike N., Yoneda M., Nakatsu Y., Ying G., Jun Z. (2008). Long-term forskolin stimulation induces AMPK activation and thereby enhances tight junction formation in human placental trophoblast BeWo cells. Placenta.

[393] El-Agroudy N.N., El-Naga R.N., El-Razeq R.A., El-Demerdash E. (2016). Forskolin, a hedgehog signalling inhibitor, attenuates carbon tetrachloride-induced liver fibrosis in rats. Br. J. Pharmacol..

[394] Ríos-Silva M., Trujillo X., Trujillo-Hernández B., Sánchez-Pastor E., Urzúa Z., Mancilla E., Huerta M. (2014). Effect of chronic administration of forskolin on glycemia and oxidative stress in rats with and without experimental diabetes. Int. J. Med. Sci..

[395] Kihira Y., Burentogtokh A., Itoh M., Izawa-Ishizawa Y., Ishizawa K., Ikeda Y., Tsuchiya K., Tamaki T. (2015). Hypoxia decreases glucagon-like peptide-1 secretion from the GLUTag cell line. Biol. Pharm. Bull..

[396] Grey K.B., Burrell B.D. (2008). Forskolin induces NMDA receptor-dependent potentiation at a central synapse in the leech. J. Neurophysiol..

[397] Morris D.I., Speicher L.A., Ruoho A.E., Tew K.D., Seamon K.B. (1991). Interaction of forskolin with the P-glycoprotein multidrug transporter. Biochemistry.

[398] Namanja H.A., Emmert D., Pires M.M., Hrycyna C.A., Chmielewski J. (2009). Inhibition of human P-glycoprotein transport and substrate binding using a galantamine dimer. Biochem. Biophys. Res. Commun..

[399] McNulty J., Nair J.J., Singh M., Crankshaw D.J., Holloway A.C., Bastida J. (2009). Selective cytochrome P450 3A4 inhibitory activity of Amaryllidaceae alkaloids. Bioorg. Med. Chem. Lett..

[400] Huang F., Fu Y. (2010). A review of clinical pharmacokinetics and pharmacodynamics of galantamine, a reversible acetylcholinesterase inhibitor for the treatment of Alzheimer’s disease, in healthy subjects and patients. Curr. Clin. Pharmacol..

[401] Jann M.W., Shirley K.L., Small G.W. (2002). Clinical pharmacokinetics and pharmacodynamics of cholinesterase inhibitors. Clin. Pharmacokinet..

[402] Cacabelos R. (2020). Pharmacogenetic considerations when prescribing cholinesterase inhibitors for the treatment of Alzheimer’s disease. Expert Opin. Drug Metab. Toxicol..

[403] Amaravani M., Prasad N.K., Ramakrishna V. (2012). COX-2 structural analysis and docking studies with gallic acid structural analogues. Springerplus.

[404] Ponnusankar S., Pandit S., Babu R., Bandyopadhyay A., Mukherjee P.K. (2011). Cytochrome P450 inhibitory potential of Triphala—A Rasayana from Ayurveda. J. Ethnopharmacol..

[405] Athukuri B.L., Neerati P. (2016). Enhanced oral bioavailability of metoprolol with gallic acid and ellagic acid in male Wistar rats: Involvement of CYP2D6 inhibition. Drug Metab. Pers. Ther..

[406] Chhillar R., Dhingra D. (2013). Antidepressant-like activity of gallic acid in mice subjected to unpredictable chronic mild stress. Fundam. Clin. Pharmacol..

[407] Kitagawa S., Nabekura T., Kamiyama S., Takahashi T., Nakamura Y., Kashiwada Y., Ikeshiro Y. (2005). Effects of alkyl gallates on P-glycoprotein function. Biochem. Pharmacol..

[408] Kahkeshani N., Farzaei F., Fotouhi M., Alavi S.S., Bahramsoltani R., Naseri R., Momtaz S., Abbasabadi Z., Rahimi R., Farzaei M.H. (2019). Pharmacological effects of gallic acid in health and diseases: A mechanistic review. Iran. J. Basic Med. Sci..

[409] Li Z., Wang K., Zheng J., Cheung F.S.G., Chan T., Zhu L., Zhou F. (2014). Interactions of the active components of *Punica granatum* (pomegranate) with the essential renal and hepatic human Solute Carrier transporters. Pharm. Biol..

[410] Palacios-González B., Zarain-Herzberg A., Flores-Galicia I., Noriega L.G., Alemán-Escondrillas G., Zariñan T., Ulloa-Aguirre A., Torres N., Tovar A.R. (2014). Genistein stimulates fatty acid oxidation in a leptin receptor-independent manner through the JAK2-mediated phosphorylation and activation of AMPK in skeletal muscle. Biochim. Biophys. Acta.

[411] Tuli H.S., Tuorkey M.J., Thakral F., Sak K., Kumar M., Sharma A.K., Sharma U., Jain A., Aggarwal V., Bishayee A. (2019). Molecular Mechanisms of Action of Genistein in Cancer: Recent Advances. Front. Pharmacol..

[412] Lepri S.R., Sartori D., Semprebon S.C., Baranoski A., Coatti G.C., Mantovani M.S. (2018). Genistein Affects Expression of Cytochrome P450 (CYP450) Genes in Hepatocellular Carcinoma (HEPG2/C3A) Cell Line. Drug Metab. Lett..

[413] Notarnicola M., Messa C., Orlando A., D’Attoma B., Tutino V., Rivizzigno R., Caruso M.G. (2008). Effect of genistein on cholesterol metabolism-related genes in a colon cancer cell line. Genes Nutr..

[414] Zarmouh N.O., Messeha S.S., Elshami F.M., Soliman K.F.A. (2016). Evaluation of the Isoflavone Genistein as Reversible Human Monoamine Oxidase-A and -B Inhibitor. Evid. Based Complement. Altern. Med..

[415] Huang R., Singh M., Dillon G.H. (2010). Genistein directly inhibits native and recombinant NMDA receptors. Neuropharmacology.

[416] Cho H.-J., Yoon I.-S. (2015). Pharmacokinetic interactions of herbs with cytochrome p450 and p-glycoprotein. Evid. Based Complement. Altern. Med..

[417] Qiu J.X., Zhou Z.W., He Z.X., Zhang X., Zhou S.F., Zhu S. (2015). Estimation of the binding modes with important human cytochrome P450 enzymes, drug interaction potential, pharmacokinetics, and hepatotoxicity of ginger components using molecular docking, computational, and pharmacokinetic modeling studies. Drug Des. Dev. Ther..

[418] Bogacz A., Deka-Pawlik D., Bartkowiak-Wieczorek J., Karasiewicz M., Kujawski R., Kowalska A., Chałas A., Czerny B., Grześkowiak E., Mrozikiewicz P.M. (2013). The effect of herbal materials on the p-glycoprotein activity and function. Herb. Pol..

[419] Li Y., Wu Y., Yao X., Hao F., Yu C., Bao Y., Wu Y., Song Z., Sun Y., Zheng L. (2017). Ginkgolide a ameliorates LPS-induced inflammatory responses in vitro and *in vivo*. Int. J. Mol. Sci..

[420] Kuo L.-C., Song Y.-Q., Yao C.-A., Cheng I.H., Chien C.-T., Lee G.-C., Yang W.-C., Lin Y. (2019). Ginkgolide A Prevents the Amyloid-β-Induced Depolarization of Cortical Neurons. J. Agric. Food Chem..

[421] Ramanathan M.R., Penzak S.R. (2017). Pharmacokinetic Drug Interactions with Panax ginseng. Eur. J. Drug Metab. Pharmacokinet..

[422] Wu L., Liu J., Hou J., Zhan T., Yuan L., Liu F., Xiong Y., Hu J., Xia C. (2020). Interactions of the major effective components in Shengmai formula with breast cancer resistance protein at the cellular and vesicular levels. Biomed. Pharmacother..

[423] Jiang R., Dong J., Li X., Du F., Jia W., Xu F., Wang F., Yang J., Niu W., Li C. (2015). Molecular mechanisms governing different pharmacokinetics of ginsenosides and potential for ginsenoside-perpetrated herb-drug interactions on OATP1B3. Br. J. Pharmacol..

[424] Zhang J., Li L., Kim S.-H., Hagerman A.E., Lü J. (2009). Anti-cancer, anti-diabetic and other pharmacologic and biological activities of penta-galloyl-glucose. Pharm. Res..

[425] Lee S.-J., Lee I.-S., Mar W. (2003). Inhibition of inducible nitric oxide synthase and cyclooxygenase-2 activity by 1,2,3,4,6-penta-*O*-galloyl-beta-d-glucose in murine macrophage cells. Arch. Pharm. Res..

[426] Wen F., Shi M., Bian J., Zhang H., Gui C. (2016). Identification of natural products as modulators of OATP2B1 using LC-MS/MS to quantify OATP-mediated uptake. Pharm. Biol..

[427] Tai T., Huang X., Su Y., Ji J., Su Y., Jiang Z., Zhang L. (2014). Glycyrrhizin accelerates the metabolism of triptolide through induction of CYP3A in rats. J. Ethnopharmacol..

[428] Hou Y.-C., Lin S.-P., Chao P.-D.L. (2012). Liquorice reduced cyclosporine bioavailability by activating P-glycoprotein and CYP 3A. Food Chem..

[429] Chen Y., Chen L., Zhang H., Huang S., Xiong Y., Xia C. (2018). Interaction of Sulfonylureas with Liver Uptake Transporters OATP1B1 and OATP1B3. Basic Clin. Pharmacol. Toxicol..

[430] Ishola A.A., Adewole K.E. (2019). In Silico screening of anticholinesterase alkaloids for cyclooxygenase-2 (COX-2) and matrix metalloproteinase 8 (MMP-8) inhibitory potentials as multi-target inhibitors of Alzheimer’s disease. Med. Chem. Res..

[431] Modarai M., Suter A., Kortenkamp A., Heinrich M. (2011). The interaction potential of herbal medicinal products: A luminescence-based screening platform assessing effects on cytochrome P450 and its use with devil’s claw (*Harpagophyti radix*) preparations: Screening for CYP P450-HMP interactions. J. Pharm. Pharmacol..

[432] Anauate M.C., Torres L.M., de Mello S.B.V. (2010). Effect of isolated fractions of Harpagophytum procumbens D.C. (devil’s claw) on COX-1, COX-2 activity and nitric oxide production on whole-blood assay. Phytother. Res..

[433] Zhang L., Feng L., Jia Q., Xu J., Wang R., Wang Z., Wu Y., Li Y. (2011). Effects of β-glucosidase hydrolyzed products of harpagide and harpagoside on cyclooxygenase-2 (COX-2) in vitro. Bioorg. Med. Chem..

[434] Unger M., Frank A. (2004). Simultaneous determination of the inhibitory potency of herbal extracts on the activity of six major cytochrome P450 enzymes using liquid chromatography/mass spectrometry and automated online extraction. Rapid Commun. Mass Spectrom..

[435] Fei Z., Hu M., Baum L., Kwan P., Hong T., Zhang C. (2020). The potential role of human multidrug resistance protein 1 (MDR1) and multidrug resistance-associated protein 2 (MRP2) in the transport of Huperzine A in vitro. Xenobiotica.

[436] Ferreira A., Rodrigues M., Fortuna A., Falcão A., Alves G. (2016). Huperzine A from *Huperzia serrata*: A review of its sources, chemistry, pharmacology and toxicology. Phytochem. Rev..

[437] Ma X., Wang H., Xin J., Zhang T., Tu Z. (2003). Identification of cytochrome P450 1A2 as enzyme involved in the microsomal metabolism of Huperzine A. Eur. J. Pharmacol..

[438] Madabushi R., Frank B., Drewelow B., Derendorf H., Butterweck V. (2006). Hyperforin in St. John’s wort drug interactions. Eur. J. Clin. Pharmacol..

[439] Chen X., Ji Z.L., Chen Y.Z. (2002). TTD: Therapeutic Target Database. Nucleic Acids Res..

[440] Koeberle A., Rossi A., Bauer J., Dehm F., Verotta L., Northoff H., Sautebin L., Werz O. (2011). Hyperforin, an Anti-Inflammatory Constituent from St. John’s Wort, Inhibits Microsomal Prostaglandin E(2) Synthase-1 and Suppresses Prostaglandin E(2) Formation *in vivo*. Front. Pharmacol..

[441] Quiney C., Billard C., Salanoubat C., Fourneron J.D., Kolb J.P. (2006). Hyperforin, a new lead compound against the progression of cancer and leukemia?. Leukemia.

[442] Albert D., Zündorf I., Dingermann T., Müller W.E., Steinhilber D., Werz O. (2002). Hyperforin is a dual inhibitor of cyclooxygenase-1 and 5-lipoxygenase. Biochem. Pharmacol..

[443] Schäfer A.M., Potterat O., Seibert I., Fertig O., Meyer Zu Schwabedissen H.E. (2019). Hyperforin-Induced Activation of the Pregnane X Receptor Is Influenced by the Organic Anion-Transporting Polypeptide 2B1. Mol. Pharmacol..

[444] Nicolussi S., Drewe J., Butterweck V., Meyer Zu Schwabedissen H.E. (2020). Clinical relevance of St. John’s wort drug interactions revisited. Br. J. Pharmacol..

[445] Kumar V., Mdzinarishvili A., Kiewert C., Abbruscato T., Bickel U., van der Schyf C.J., Klein J. (2006). NMDA receptor-antagonistic properties of hyperforin, a constituent of St. John’s Wort. J. Pharmacol. Sci..

[446] Tirona R.G., Leake B.F., Wolkoff A.W., Kim R.B. (2003). Human organic anion transporting polypeptide-C (SLC21A6) is a major determinant of rifampin-mediated pregnane X receptor activation. J. Pharmacol. Exp. Ther..

[447] Gutmann H., Poller B., Büter K.B., Pfrunder A., Schaffner W., Drewe J. (2006). *Hypericum perforatum*: Which constituents may induce intestinal MDR1 and CYP3A4 mRNA expression?. Planta Med..

[448] Smith N.F., Acharya M.R., Desai N., Figg W.D., Sparreboom A. (2005). Identification of OATP1B3 as a high-affinity hepatocellular transporter of paclitaxel. Cancer Biol. Ther..

[449] Quiney C., Billard C., Faussat A.-M., Salanoubat C., Kolb J.-P. (2007). Hyperforin inhibits P-gp and BCRP activities in chronic lymphocytic leukaemia cells and myeloid cells. Leuk. Lymphoma.

[450] You M.-K., Kim H.-J., Kook J.H., Kim H.-A. (2018). St. John’s Wort Regulates Proliferation and Apoptosis in MCF-7 Human Breast Cancer Cells by Inhibiting AMPK/mTOR and Activating the Mitochondrial Pathway. Int. J. Mol. Sci..

[451] Novelli M., Masiello P., Beffy P., Menegazzi M. (2020). Protective Role of St. John’s Wort and Its Components Hyperforin and Hypericin against Diabetes through Inhibition of Inflammatory Signaling: Evidence from In Vitro and In Vivo Studies. Int. J. Mol. Sci..

[452] Suzuki O., Katsumata Y., Oya M., Bladt S., Wagner H. (1984). Inhibition of monoamine oxidase by hypericin. Planta Med..

[453] Cott J.M. (1997). In vitro receptor binding and enzyme inhibition by *Hypericum perforatum* extract. Pharmacopsychiatry.

[454] Šemeláková M., Jendželovský R., Fedoročko P. (2016). Drug membrane transporters and CYP3A4 are affected by hypericin, hyperforin or aristoforin in colon adenocarcinoma cells. Biomed. Pharmacother..

[455] Khot M.I., Perry S.L., Maisey T., Armstrong G., Andrew H., Hughes T.A., Kapur N., Jayne D.G. (2018). Inhibiting ABCG2 could potentially enhance the efficacy of hypericin-mediated photodynamic therapy in spheroidal cell models of colorectal cancer. Photodiagnosis Photodyn. Ther..

[456] Song M., Hong M., Lee M.Y., Jee J.-G., Lee Y.M., Bae J.-S., Jeong T.C., Lee S. (2013). Selective inhibition of the cytochrome P450 isoform by hyperoside and its potent inhibition of CYP2D6. Food Chem. Toxicol..

[457] Li G., Simmler C., Chen L., Nikolic D., Chen S.-N., Pauli G.F., van Breemen R.B. (2017). Cytochrome P450 inhibition by three licorice species and fourteen licorice constituents. Eur. J. Pharm. Sci..

[458] Wang Y., Cao J., Zeng S. (2005). Involvement of P-glycoprotein in regulating cellular levels of Ginkgo flavonols: Quercetin, kaempferol, and isorhamnetin. J. Pharm. Pharmacol..

[459] Mohos V., Fliszár-Nyúl E., Ungvári O., Kuffa K., Needs P.W., Kroon P.A., Telbisz Á., Özvegy-Laczka C., Poór M. (2020). Inhibitory Effects of Quercetin and Its Main Methyl, Sulfate, and Glucuronic Acid Conjugates on Cytochrome P450 Enzymes, and on OATP, BCRP and MRP2 Transporters. Nutrients.

[460] Park J.-W., Choi J.-S. (2019). Role of kaempferol to increase bioavailability and pharmacokinetics of nifedipine in rats. Chin. J. Nat. Med..

[461] Piao Y., Shin S.-C., Choi J.-S. (2008). Effects of oral kaempferol on the pharmacokinetics of tamoxifen and one of its metabolites, 4-hydroxytamoxifen, after oral administration of tamoxifen to rats. Biopharm. Drug Dispos..

[462] Silva B., Oliveira P.J., Dias A., Malva J.O. (2008). Quercetin, kaempferol and biapigenin from *Hypericum perforatum* are neuroprotective against excitotoxic insults. Neurotox. Res..

[463] Navrátilová L., Applová L., Horký P., Mladěnka P., Pávek P., Trejtnar F. (2018). Interaction of soy isoflavones and their main metabolites with hOATP2B1 transporter. Naunyn. Schmiedebergs. Arch. Pharmacol..

[464] Vautier S., Milane A., Fernandez C., Buyse M., Chacun H., Farinotti R. (2008). Interactions between antiparkinsonian drugs and ABCB1/P-glycoprotein at the blood-brain barrier in a rat brain endothelial cell model. Neurosci. Lett..

[465] Blanchet P.J., Papa S.M., Metman L.V., Mouradian M.M., Chase T.N. (1997). Modulation of levodopa-induced motor response complications by NMDA antagonists in Parkinson’s disease. Neurosci. Biobehav. Rev..

[466] Hwang J.-T., Park O.J., Lee Y.K., Sung M.J., Hur H.J., Kim M.S., Ha J.H., Kwon D.Y. (2011). Anti-tumor effect of luteolin is accompanied by AMP-activated protein kinase and nuclear factor-κB modulation in HepG2 hepatocarcinoma cells. Int. J. Mol. Med..

[467] Harris G.K., Qian Y., Leonard S.S., Sbarra D.C., Shi X. (2006). Luteolin and chrysin differentially inhibit cyclooxygenase-2 expression and scavenge reactive oxygen species but similarly inhibit prostaglandin-E2 formation in RAW 264.7 cells. J. Nutr..

[468] Cao L., Kwara A., Greenblatt D.J. (2017). Metabolic interactions between acetaminophen (paracetamol) and two flavonoids, luteolin and quercetin, through *in-vitro* inhibition studies. J. Pharm. Pharmacol..

[469] Quintieri L., Palatini P., Nassi A., Ruzza P., Floreani M. (2008). Flavonoids diosmetin and luteolin inhibit midazolam metabolism by human liver microsomes and recombinant CYP 3A4 and CYP3A5 enzymes. Biochem. Pharmacol..

[470] Wong T.Y., Lin S.-M., Leung L.K. (2015). The Flavone Luteolin Suppresses SREBP-2 Expression and Post-Translational Activation in Hepatic Cells. PLoS ONE.

[471] Han X.H., Hong S.S., Hwang J.S., Lee M.K., Hwang B.Y., Ro J.S. (2007). Monoamine oxidase inhibitory components from *Cayratia japonica*. Arch. Pharm. Res..

[472] Boersma M.G., van der Woude H., Bogaards J., Boeren S., Vervoort J., Cnubben N.H.P., van Iersel M.L.P.S., van Bladeren P.J., Rietjens I.M.C.M. (2002). Regioselectivity of phase II metabolism of luteolin and quercetin by UDP-glucuronosyl transferases. Chem. Res. Toxicol..

[473] McNulty J., Nair J.J., Singh M., Crankshaw D.J., Holloway A.C., Bastida J. (2010). Cytochrome P450 3A4 inhibitory activity studies within the lycorine series of alkaloids. Nat. Prod. Commun..

[474] Hanley M.J., Masse G., Harmatz J.S., Cancalon P.F., Dolnikowski G.G., Court M.H., Greenblatt D.J. (2013). Effect of blueberry juice on clearance of buspirone and flurbiprofen in human volunteers. Br. J. Clin. Pharmacol..

[475] Li Y., Xu Y., Xie J., Chen W. (2020). Malvidin-3-*O*-arabinoside ameliorates ethyl carbamate-induced oxidative damage by stimulating AMPK-mediated autophagy. Food Funct..

[476] Showande S.J., Fakeye T.O., Kajula M., Hokkanen J., Tolonen A. (2019). Potential inhibition of major human cytochrome P450 isoenzymes by selected tropical medicinal herbs—Implication for herb–drug interactions. Food Sci. Nutr..

[477] Rodeiro I., José Gómez-Lechón M., Perez G., Hernandez I., Herrera J.A., Delgado R., Castell J.V., Teresa Donato M. (2013). *Mangifera indica* L. extract and mangiferin modulate cytochrome P450 and UDP-glucuronosyltransferase enzymes in primary cultures of human hepatocytes. Phytother. Res..

[478] Leslie E.M., Mao Q., Oleschuk C.J., Deeley R.G., Cole S.P. (2001). Modulation of multidrug resistance protein 1 (MRP1/ABCC1) transport and atpase activities by interaction with dietary flavonoids. Mol. Pharmacol..

[479] Surya Sandeep M., Sridhar V., Puneeth Y., Ravindra Babu P., Naveen Babu K. (2014). Enhanced oral bioavailability of felodipine by naringenin in Wistar rats and inhibition of P-glycoprotein in everted rat gut sacs in vitro. Drug Dev. Ind. Pharm..

[480] Zygmunt K., Faubert B., MacNeil J., Tsiani E. (2010). Naringenin, a citrus flavonoid, increases muscle cell glucose uptake via AMPK. Biochem. Biophys. Res. Commun..

[481] Chao C.-L., Weng C.-S., Chang N.-C., Lin J.-S., Kao S.-T., Ho F.-M. (2010). Naringenin more effectively inhibits inducible nitric oxide synthase and cyclooxygenase-2 expression in macrophages than in microglia. Nutr. Res..

[482] Cheng K., Zeng X., Wu H., Su W., Fan W., Bai Y., Yao H., Li P. (2019). Effects of Naringin on the Activity and mRNA Expression of CYP Isozymes in Rats. Nat. Prod. Commun..

[483] Ho P.C., Saville D.J., Coville P.F., Wanwimolruk S. (2000). Content of CYP3A4 inhibitors, naringin, naringenin and bergapten in grapefruit and grapefruit juice products. Pharm. Acta Helv..

[484] Lu W.J., Ferlito V., Xu C., Flockhart D.A., Caccamese S. (2011). Enantiomers of naringenin as pleiotropic, stereoselective inhibitors of cytochrome P450 isoforms. Chirality.

[485] Lee S.H., Park Y.B., Bae K.H., Bok S.H., Kwon Y.K., Lee E.S., Choi M.S. (1999). Cholesterol-lowering activity of naringenin via inhibition of 3-hydroxy-3-methylglutaryl coenzyme A reductase and acyl coenzyme A:cholesterol acyltransferase in rats. Ann. Nutr. Metab..

[486] Rani N., Bharti S., Krishnamurthy B., Bhatia J., Sharma C., Kamal M.A., Ojha S., Arya D.S. (2016). Pharmacological Properties and Therapeutic Potential of Naringenin: A Citrus Flavonoid of Pharmaceutical Promise. Curr. Pharm. Des..

[487] Satoh H., Yamashita F., Tsujimoto M., Murakami H., Koyabu N., Ohtani H., Sawada Y. (2005). Citrus juices inhibit the function of human organic anion-transporting polypeptide OATP-B. Drug Metab. Dispos..

[488] Hong S.S., Seo K., Lim S.-C., Han H.-K. (2007). Interaction characteristics of flavonoids with human organic anion transporter 1 (hOAT1) and 3 (hOAT3). Pharmacol. Res..

[489] Eagling V.A., Profit L., Back D.J. (1999). Inhibition of the CYP3A4-mediated metabolism and P-glycoprotein-mediated transport of the HIV-1 protease inhibitor saquinavir by grapefruit juice components. Br. J. Clin. Pharmacol..

[490] Sui G.-G., Xiao H.-B., Lu X.-Y., Sun Z.-L. (2018). Naringin Activates AMPK Resulting in Altered Expression of SREBPs, PCSK9, and LDLR To Reduce Body Weight in Obese C57BL/6J Mice. J. Agric. Food Chem..

[491] Zeng L., Zhen Y., Chen Y., Zou L., Zhang Y., Hu F., Feng J., Shen J., Wei B. (2014). Naringin inhibits growth and induces apoptosis by a mechanism dependent on reduced activation of NF-κB/COX-2-caspase-1 pathway in HeLa cervical cancer cells. Int. J. Oncol..

[492] Choi M.S., Do K.M., Park Y.S., Jeon S.M., Jeong T.S., Lee Y.K., Lee M.K., Bok S.H. (2001). Effect of naringin supplementation on cholesterol metabolism and antioxidant status in rats fed high cholesterol with different levels of vitamin E. Ann. Nutr. Metab..

[493] Shirasaka Y., Shichiri M., Mori T., Nakanishi T., Tamai I. (2013). Major active components in grapefruit, orange, and apple juices responsible for OATP2B1-mediated drug interactions. J. Pharm. Sci..

[494] Nabekura T., Yamaki T., Kitagawa S. (2008). Effects of chemopreventive citrus phytochemicals on human P-glycoprotein and multidrug resistance protein 1. Eur. J. Pharmacol..

[495] Huang H., Li L., Shi W., Liu H., Yang J., Yuan X., Wu L. (2016). The Multifunctional Effects of Nobiletin and Its Metabolites In Vivo and *In Vitro*. Evid. Based Complement. Altern. Med..

[496] Weiss J., Gattuso G., Barreca D., Haefeli W.E. (2020). Nobiletin, sinensetin, and tangeretin are the main perpetrators in clementines provoking food-drug interactions *in vitro*. Food Chem..

[497] Bajraktari-Sylejmani G., Weiss J. (2020). Potential Risk of Food-Drug Interactions: Citrus Polymethoxyflavones and Flavanones as Inhibitors of the Organic Anion Transporting Polypeptides (OATP) 1B1, 1B3, and 2B1. Eur. J. Drug Metab. Pharmacokinet..

[498] Braga F., Ayres-Saraiva D., Gattass C.R., Capella M.A.M. (2007). Oleanolic acid inhibits the activity of the multidrug resistance protein ABCC1 (MRP1) but not of the ABCB1 (P-glycoprotein): Possible use in cancer chemotherapy. Cancer Lett..

[499] Liu L., Li H., Hu K., Xu Q., Wen X., Cheng K., Chen C., Yuan H., Dai L., Sun H. (2020). Synthesis and anti-inflammatory activity of saponin derivatives of δ-oleanolic acid. Eur. J. Med. Chem..

[500] Martínez-González J., Rodríguez-Rodríguez R., González-Díez M., Rodríguez C., Herrera M.D., Ruiz-Gutierrez V., Badimon L. (2008). Oleanolic acid induces prostacyclin release in human vascular smooth muscle cells through a cyclooxygenase-2-dependent mechanism. J. Nutr..

[501] Kim K.-A., Lee J.-S., Park H.-J., Kim J.-W., Kim C.-J., Shim I.-S., Kim N.-J., Han S.-M., Lim S. (2004). Inhibition of cytochrome P450 activities by oleanolic acid and ursolic acid in human liver microsomes. Life Sci..

[502] Shen C., Huang L., Xiang H., Deng M., Gao H., Zhu Z., Liu M., Luo G. (2016). Inhibitory effects on the HMG-CoA reductase in the chemical constituents of the *Cassia mimosoides* Linn. Rev. Romana Med. Lab..

[503] Fajemiroye J.O., Polepally P.R., Chaurasiya N.D., Tekwani B.L., Zjawiony J.K., Costa E.A. (2015). Oleanolic acid acrylate elicits antidepressant-like effect mediated by 5-HT1A receptor. Sci. Rep..

[504] Rodrigues M., Alves G., Falcão A. (2013). Investigating herb-drug interactions: The effect of *Citrus aurantium* fruit extract on the pharmacokinetics of amiodarone in rats. Food Chem. Toxicol..

[505] Tiesjema B., Jeurissen S.M.F., De Wit L., Mol H., Fragki S., Razenberg L. (2017). Risk Assessment of Synephrine.

[506] Suzuki O., Matsumoto T., Oya M., Katsumata Y. (1979). Oxidation of synephrine by type A and type B monoamine oxidase. Experientia.

[507] Morsy M.A., Abdel-Aziz A.M., Abdel-Hafez S.M.N., Venugopala K.N., Nair A.B., Abdel-Gaber S.A. (2020). The Possible Contribution of P-Glycoprotein in the Protective Effect of Paeonol against Methotrexate-Induced Testicular Injury in Rats. Pharmaceuticals.

[508] Li M., Tan S.-Y., Wang X.-F. (2014). Paeonol exerts an anticancer effect on human colorectal cancer cells through inhibition of PGE₂ synthesis and COX-2 expression. Oncol. Rep..

[509] Liu H.-X., Hu Y., Liu Y., He Y.-Q., Li W., Yang L. (2009). CYP1A2 is the major isoform responsible for paeonol o-demethylation in human liver microsomes. Xenobiotica.

[510] Cai J., Chen S., Zhang W., Hu S., Lu J., Xing J., Dong Y. (2014). Paeonol reverses paclitaxel resistance in human breast cancer cells by regulating the expression of transgelin 2. Phytomedicine.

[511] Kong L.D., Cheng C.H.K., Tan R.X. (2004). Inhibition of MAO A and B by some plant-derived alkaloids, phenols and anthraquinones. J. Ethnopharmacol..

[512] Ekeuku S.O., Pang K.-L., Chin K.-Y. (2020). Palmatine as an Agent Against Metabolic Syndrome and Its Related Complications: A Review. Drug Des. Dev. Ther..

[513] Lo S.-N., Chang Y.-P., Tsai K.-C., Chang C.-Y., Wu T.-S., Ueng Y.-F. (2013). Inhibition of CYP1 by berberine, palmatine, and jatrorrhizine: Selectivity, kinetic characterization, and molecular modeling. Toxicol. Appl. Pharmacol..

[514] Vrba J., Papouskova B., Pyszkova M., Zatloukalova M., Lemr K., Ulrichova J., Vacek J. (2015). Metabolism of palmatine by human hepatocytes and recombinant cytochromes P450. J. Pharm. Biomed. Anal..

[515] Tarabasz D., Kukula-Koch W. (2020). Palmatine: A review of pharmacological properties and pharmacokinetics. Phytother. Res..

[516] Sukhaphirom N., Vardhanabhuti N., Chirdchupunseree H., Pramyothin P., Jianmongkol S. (2013). Phyllanthin and hypophyllanthin inhibit function of P-gp but not MRP2 in Caco-2 cells. J. Pharm. Pharmacol..

[517] Taesotikul T., Dumrongsakulchai W., Wattanachai N., Navinpipat V., Somanabandhu A., Tassaneeyakul W., Tassaneeyakul W. (2011). Inhibitory effects of *Phyllanthus amarus* and its major lignans on human microsomal cytochrome P450 activities: Evidence for CYP3A4 mechanism-based inhibition. Drug Metab. Pharmacokinet..

[518] Zhang F., Ai W., Hu X., Meng Y., Yuan C., Su H., Wang L., Zhu X., Gao P., Shu G. (2018). Phytol stimulates the browning of white adipocytes through the activation of AMP-activated protein kinase (AMPK) α in mice fed high-fat diet. Food Funct..

[519] Islam M.T., Ray P., Khalipha A.B.R., Hafiz Hassan S.M., Khan M.R., Rouf R. (2020). Molecular docking study of the phytol and its derivatives against COX-2 induced inflammation: A combined density functional study. RRST.

[520] de Moraes J., de Oliveira R.N., Costa J.P., Junior A.L.G., de Sousa D.P., Freitas R.M., Allegretti S.M., Pinto P.L.S. (2014). Phytol, a diterpene alcohol from chlorophyll, as a drug against neglected tropical disease Schistosomiasis mansoni. PLoS Negl. Trop. Dis..

[521] Li X.-J., Yang Y.-J., Li Y.-S., Zhang W.K., Tang H.-B. (2016). α-Pinene, linalool, and 1-octanol contribute to the topical anti-inflammatory and analgesic activities of frankincense by inhibiting COX-2. J. Ethnopharmacol..

[522] Wilderman P.R., Shah M.B., Jang H.-H., Stout C.D., Halpert J.R. (2013). Structural and thermodynamic basis of (+)-α-pinene binding to human cytochrome P450 2B6. J. Am. Chem. Soc..

[523] Ueno H., Shimada A., Suemitsu S., Murakami S., Kitamura N., Wani K., Matsumoto Y., Okamoto M., Ishihara T. (2019). Attenuation Effects of Alpha-Pinene Inhalation on Mice with Dizocilpine-Induced Psychiatric-Like Behaviour. Evid. Based Complement. Altern. Med..

[524] Lee S.H., Kim H.Y., Back S.Y., Han H.-K. (2018). Piperine-mediated drug interactions and formulation strategy for piperine: Recent advances and future perspectives. Expert Opin. Drug Metab. Toxicol..

[525] Han Y., Tan T.M.C., Lim L.-Y. (2008). In vitro and in vivo evaluation of the effects of piperine on P-gp function and expression. Toxicol. Appl. Pharmacol..

[526] Li S., Lei Y., Jia Y., Li N., Wink M., Ma Y. (2011). Piperine, a piperidine alkaloid from Piper nigrum re-sensitizes P-gp, MRP1 and BCRP dependent multidrug resistant cancer cells. Phytomedicine.

[527] Li Z.-L., Dong X.-Z., Wang D.-X., Dong R.-H., Guo T.-T., Sun Y., Liu P. (2014). Effect of oligosaccharide esters and polygalaxanthone Ill from *Polygala tenuifolia* willd towards cytochrome P450. Zhongguo Zhong Yao Za Zhi.

[528] Krajka-Kuźniak V., Szaefer H., Baer-Dubowska W. (2005). Modulation of cytochrome P450 and phase II enzymes by protocatechuic acid in mouse liver and kidney. Toxicology.

[529] Zhang M.-F., Liu Y.-X., Jiang K.-Y., Niu H.-M., Jiang J.-L., Dong S.-T., Wang X., Wang D.-F., Meng S.-N. (2019). Alteration of UDP-glucuronosyltransferase 1a1, 1a7 and P-glycoprotein expression in hepatic fibrosis rats and the impact on pharmacokinetics of puerarin. Phytomedicine.

[530] Wang L., Shan H., Wang B., Wang N., Zhou Z., Pan C., Wang F. (2018). Puerarin Attenuates Osteoarthritis via Upregulating AMP-Activated Protein Kinase/Proliferator-Activated Receptor-γ Coactivator-1 Signaling Pathway in Osteoarthritis Rats. Pharmacology.

[531] Hu W., Yang X., Zhe C., Zhang Q., Sun L., Cao K. (2011). Puerarin inhibits iNOS, COX-2 and CRP expression via suppression of NF-κB activation in LPS-induced RAW264.7 macrophage cells. Pharmacol. Rep..

[532] Ge B., Zhang Z., Lam T.T., Zuo Z. (2017). Puerarin offsets the anticoagulation effect of warfarin in rats by inducing rCyps, upregulating vitamin K epoxide reductase and inhibiting thrombomodulin. Biopharm. Drug Dispos..

[533] Zheng J., Chen B., Jiang B., Zeng L., Tang Z.-R., Fan L., Zhou H.-H. (2010). The effects of puerarin on CYP2D6 and CYP1A2 activities *in vivo*. Arch. Pharm. Res..

[534] Guo Y.-J., Liang D.-L., Xu Z.-S., Ye Q. (2014). In vivo inhibitory effects of puerarin on selected rat cytochrome P450 isoenzymes. Pharmazie.

[535] Hwang Y.P., Choi C.Y., Chung Y.C., Jeon S.S., Jeong H.G. (2007). Protective effects of puerarin on carbon tetrachloride-induced hepatotoxicity. Arch. Pharm. Res..

[536] Chung M.J., Sung N.-J., Park C.-S., Kweon D.-K., Mantovani A., Moon T.-W., Lee S.-J., Park K.-H. (2008). Antioxidative and hypocholesterolemic activities of water-soluble puerarin glycosides in HepG2 cells and in C57 BL/6J mice. Eur. J. Pharmacol..

[537] Zhang R., Guo H.-N., Wu H.-Q., Cheng H.-X., Wang H.-Q. (2011). Effect of puerarin on the expression of NMDA receptor in the hippocampus CA1 region after focal cerebral ischemia in rats. Sichuan Da Xue Xue Bao Yi Xue Ban.

[538] Borska S., Sopel M., Chmielewska M., Zabel M., Dziegiel P. (2010). Quercetin as a potential modulator of P-glycoprotein expression and function in cells of human pancreatic carcinoma line resistant to daunorubicin. Molecules.

[539] Yang T., Liu Y., Huang X., Zhang R., Yang C., Zhou J., Zhang Y., Wan J., Shi S. (2018). Quercetin-3-*O*-β-d-glucoside decreases the bioavailability of cyclosporin A through regulation of drug metabolizing enzymes, transporters and nuclear receptors in rats. Mol. Med. Rep..

[540] Elbarbry F., Ung A., Abdelkawy K. (2018). Studying the Inhibitory Effect of Quercetin and Thymoquinone on Human Cytochrome P450 Enzyme Activities. Pharmacogn. Mag..

[541] Walsky R.L., Gaman E.A., Obach R.S. (2005). Examination of 209 drugs for inhibition of cytochrome P450 2C8. J. Clin. Pharmacol..

[542] Chimenti F., Cottiglia F., Bonsignore L., Casu L., Casu M., Floris C., Secci D., Bolasco A., Chimenti P., Granese A. (2006). Quercetin as the active principle of *Hypericum hircinum* exerts a selective inhibitory activity against MAo-A: Extraction, biological analysis, and computational study. J. Nat. Prod..

[543] Park J.-Y., Lim M.-S., Kim S.-I., Lee H.J., Kim S.-S., Kwon Y.-S., Chun W. (2016). Quercetin-3-*O*-β-d-Glucuronide Suppresses Lipopolysaccharide-Induced JNK and ERK Phosphorylation in LPS-Challenged RAW264.7 Cells. Biomol. Ther..

[544] Aires V., Colin D.J., Doreau A., Pietro A.D., Heydel J.M., Artur Y., Latruffe N., Delmas D. (2019). P-glycoprotein 1 affects chemoactivities of resveratrol against human colorectal cancer cells. Nutrients.

[545] Detampel P., Beck M., Krähenbühl S., Huwyler J. (2012). Drug interaction potential of resveratrol. Drug Metab. Rev..

[546] Dao T.-M.A., Waget A., Klopp P., Serino M., Vachoux C., Pechere L., Drucker D.J., Champion S., Barthélemy S., Barra Y. (2011). Resveratrol increases glucose induced GLP-1 secretion in mice: A mechanism which contributes to the glycemic control. PLoS ONE.

[547] Cho I.J., Ahn J.Y., Kim S., Choi M.S., Ha T.Y. (2008). Resveratrol attenuates the expression of HMG-CoA reductase mRNA in hamsters. Biochem. Biophys. Res. Commun..

[548] Zhang Z., Hamada H., Gerk P.M. (2019). Selectivity of Dietary Phenolics for Inhibition of Human Monoamine Oxidases A and B. Biomed Res. Int..

[549] Maier-Salamon A., Böhmdorfer M., Riha J., Thalhammer T., Szekeres T., Jaeger W. (2013). Interplay between metabolism and transport of resveratrol. Ann. N. Y. Acad. Sci..

[550] Takehana S., Kubota Y., Uotsu N., Yui K., Iwata K., Shimazu Y., Takeda M. (2017). The dietary constituent resveratrol suppresses nociceptive neurotransmission via the NMDA receptor. Mol. Pain.

[551] van de Wetering K., Burkon A., Feddema W., Bot A., de Jonge H., Somoza V., Borst P. (2009). Intestinal breast cancer resistance protein (BCRP)/Bcrp1 and multidrug resistance protein 3 (MRP3)/Mrp3 are involved in the pharmacokinetics of resveratrol. Mol. Pharmacol..

[552] Thu O.K., Nilsen O.G., Hellum B. (2016). In vitro inhibition of cytochrome P-450 activities and quantification of constituents in a selection of commercial *Rhodiola rosea* products. Pharm. Biol..

[553] van Diermen D., Marston A., Bravo J., Reist M., Carrupt P.A., Hostettmann K. (2009). Monoamine oxidase inhibition by *Rhodiola rosea* L. roots. J. Ethnopharmacol..

[554] Kim S.-B., Kim K.-S., Kim D.-D., Yoon I.-S. (2019). Metabolic interactions of rosmarinic acid with human cytochrome P450 monooxygenases and uridine diphosphate glucuronosyltransferases. Biomed. Pharmacother..

[555] Li M., Yin D., Li J., Shao F., Zhang Q., Jiang Q., Zhang M., Yang Y. (2020). Rosmarinic acid, the active component of, improves gliquidone transport by regulating the expression and function of P-gp and BCRP in Caco-2 cells. Pharmazie.

[556] Mohana S., Ganesan M., Agilan B., Karthikeyan R., Srithar G., Beaulah Mary R., Ananthakrishnan D., Velmurugan D., Rajendra Prasad N., Ambudkar S.V. (2016). Screening dietary flavonoids for the reversal of P-glycoprotein-mediated multidrug resistance in cancer. Mol. Biosyst..

[557] Seo S., Lee M.-S., Chang E., Shin Y., Oh S., Kim I.-H., Kim Y. (2015). Rutin Increases Muscle Mitochondrial Biogenesis with AMPK Activation in High-Fat Diet-Induced Obese Rats. Nutrients.

[558] Fideles L.D.S., de Miranda J.A.L., Martins C.D.S., Barbosa M.L.L., Pimenta H.B., Pimentel P.V.D.S., Teixeira C.S., Scafuri M.A.S., Façanha S., de Façanha S.O. (2020). Role of Rutin in 5-Fluorouracil-Induced Intestinal Mucositis: Prevention of Histological Damage and Reduction of Inflammation and Oxidative Stress. Molecules.

[559] Karakurt S. (2016). Modulatory effects of rutin on the expression of cytochrome P450s and antioxidant enzymes in human hepatoma cells. Acta Pharm..

[560] Suganya S., Nandagopal B., Anbarasu A. (2017). Natural Inhibitors of HMG-CoA Reductase-An In silico Approach Through Molecular Docking and Simulation Studies. J. Cell. Biochem..

[561] Azam F., Abodabos H.S., Taban I.M., Rfieda A.R., Mahmood D., Anwar M.J., Khan S., Sizochenko N., Poli G., Tuccinardi T. (2019). Rutin as promising drug for the treatment of Parkinson’s disease: An assessment of MAo-B inhibitory potential by docking, molecular dynamics and DFT studies. Mol. Simul..

[562] Ogura J., Koizumi T., Segawa M., Yabe K., Kuwayama K., Sasaki S., Kaneko C., Tsujimoto T., Kobayashi M., Yamaguchi H. (2014). Quercetin-3-rhamnoglucoside (rutin) stimulates transport of organic anion compounds mediated by organic anion transporting polypeptide 2B1. Biopharm. Drug Dispos..

[563] Iriti M., Kubina R., Cochis A., Sorrentino R., Varoni E.M., Kabała-Dzik A., Azzimonti B., Dziedzic A., Rimondini L., Wojtyczka R.D. (2017). Rutin, a Quercetin Glycoside, Restores Chemosensitivity in Human Breast Cancer Cells. Phytother. Res..

[564] Dogra A., Kotwal P., Gour A., Bhatt S., Singh G., Mukherjee D., Nandi U. (2020). Description of Druglike Properties of Safranal and Its Chemistry behind Low Oral Exposure. ACS Omega.

[565] Hellum B.H., Tosse A., Hoybakk K., Thomsen M., Rohloff J., Georg Nilsen O. (2010). Potent in vitro Inhibition of CYP3A4 and P-Glycoprotein by *Rhodiola rosea*. Planta Med..

[566] Liu Y., Tang H., Liu X., Chen H., Feng N., Zhang J., Wang C., Qiu M., Yang J., Zhou X. (2019). Frontline Science: Reprogramming COX-2, 5-LOX, and CYP4A-mediated arachidonic acid metabolism in macrophages by salidroside alleviates gouty arthritis. J. Leukoc. Biol..

[567] Chiang H.-M., Chen H.-C., Wu C.-S., Wu P.-Y., Wen K.-C. (2015). Rhodiola plants: Chemistry and biological activity. J. Food Drug Anal..

[568] Wei Y.-L., Du H.-J., Lin Y.-P., Wu M.-L., Xu R.-A. (2018). Effects of salidroside on rat CYP enzymes by a cocktail of probe drugs. Iran. J. Basic Med. Sci..

[569] Coors A., Brosch M., Kahl E., Khalil R., Michels B., Laub A., Franke K., Gerber B., Fendt M. (2019). *Rhodiola rosea* root extract has antipsychotic-like effects in rodent models of sensorimotor gating. J. Ethnopharmacol..

[570] Shi X., Zhao W., Yang Y., Wu S., Lv B. (2018). Salidroside could enhance the cytotoxic effect of L-OHP on colorectal cancer cells. Mol. Med. Rep..

[571] Guo P., Wang S., Liang W., Wang W., Wang H., Zhao M., Liu X. (2017). Salvianolic acid B reverses multidrug resistance in HCT-8/VCR human colorectal cancer cells by increasing ROS levels. Mol. Med. Rep..

[572] Qin T., Rasul A., Sarfraz A., Sarfraz I., Hussain G., Anwar H., Riaz A., Liu S., Wei W., Li J. (2019). Salvianolic acid A & B: Potential cytotoxic polyphenols in battle against cancer via targeting multiple signaling pathways. Int. J. Biol. Sci..

[573] Wang Q.-L., Wu Q., Tao Y.-Y., Liu C.-H., El-Nezami H. (2011). Salvianolic acid B modulates the expression of drug-metabolizing enzymes in HepG2 cells. Hepatobiliary Pancreat. Dis. Int..

[574] Li J., Olaleye O.E., Yu X., Jia W., Yang J., Lu C., Liu S., Yu J., Duan X., Wang Y. (2019). High degree of pharmacokinetic compatibility exists between the five-herb medicine XueBiJing and antibiotics comedicated in sepsis care. Acta Pharm. Sin. B.

[575] Küblbeck J., Hakkarainen J.J., Petsalo A., Vellonen K.-S., Tolonen A., Reponen P., Forsberg M.M., Honkakoski P. (2016). Genetically Modified Caco-2 Cells With Improved Cytochrome P450 Metabolic Capacity. J. Pharm. Sci..

[576] Ren L.-X., Luo Y.-F., Li X., Zuo D.-Y., Wu Y.-L. (2006). Antidepressant-like effects of sarsasapogenin from *Anemarrhena asphodeloides* BUNGE (Liliaceae). Biol. Pharm. Bull..

[577] Qiangrong P., Wang T., Lu Q., Hu X. (2005). Schisandrin B—A novel inhibitor of P-glycoprotein. Biochem. Biophys. Res. Commun..

[578] Li W.L., Xin H.W., Su M.W., Xiong L. (2010). Inhibitory effects of schisandrin A and schisandrin B on CYP3A activity. Methods Find. Exp. Clin. Pharmacol..

[579] Xie M., Lin J., Kang J., Zheng X., Fang K., Luo Y., Liu A., Yang J. (2020). Schisandrin B and Schisandrol B induce mouse CYP2b10 associated with CAR not PXR. Phytochem. Lett..

[580] Chiu P.Y., Leung H.Y., Poon M.K.T., Lee S.S.T., Ko K.M. (2006). Schisandrin B induced antioxidant response is partly mediated by cytochrome P-4502E1 catalyzed reaction in mouse liver. Mol. Cell. Biochem..

[581] Lu Y., Hu Q., Chen L., Zhang H., Huang S., Xiong Y., Xia C. (2019). Interaction of deoxyschizandrin and schizandrin B with liver uptake transporters OATP1B1 and OATP1B3. Xenobiotica.

[582] Singla R., Jaitak V. (2014). Shatavari (*Asparagus racemosus* wild): A review on its cultivation, morphology, phytochemistry and pharmacological importance. Int. J. Pharm. Sci. Res..

[583] Azam F., Amer A.M., Abulifa A.R., Elzwawi M.M. (2014). Ginger components as new leads for the design and development of novel multi-targeted anti-Alzheimer’s drugs: A computational investigation. Drug Des. Dev. Ther..

[584] Kim J. (2016). Effects of 6-Shogaol, A Major Component of *Zingiber officinale* Roscoe, on Human Cytochrome P450 Enzymes in vitro. Korean J. Med. Crop. Sci..

[585] Fajrin F.A., Nugroho A.E., Nurrochmad A., Susilowati R. (2020). Ginger extract and its compound, 6-shogaol, attenuates painful diabetic neuropathy in mice via reducing TRPV1 and NMDAR2B expressions in the spinal cord. J. Ethnopharmacol..

[586] Wang P., Zhao Y., Zhu Y., Sang S. (2017). Glucuronidation and its impact on the bioactivity of [6]-shogaol. Mol. Nutr. Food Res..

[587] Silva N., Salgueiro L., Fortuna A., Cavaleiro C. (2016). P-glycoprotein Mediated Efflux Modulators of Plant Origin: A Short Review. Nat. Prod. Commun..

[588] Hwang S.-L., Kim H.-N., Jung H.-H., Kim J.-E., Choi D.-K., Hur J.-M., Lee J.-Y., Song H., Song K.-S., Huh T.-L. (2008). Beneficial effects of beta-sitosterol on glucose and lipid metabolism in L6 myotube cells are mediated by AMP-activated protein kinase. Biochem. Biophys. Res. Commun..

[589] Sun Y., Gao L., Hou W., Wu J. (2020). β-Sitosterol Alleviates Inflammatory Response via Inhibiting the Activation of ERK/p38 and NF-κB Pathways in LPS-Exposed BV2 Cells. Biomed Res. Int..

[590] Nair V.D.P., Foster B.C., Thor Arnason J., Mills E.J., Kanfer I. (2007). In vitro evaluation of human cytochrome P450 and P-glycoprotein-mediated metabolism of some phytochemicals in extracts and formulations of African potato. Phytomedicine.

[591] Rosenblat M., Volkova N., Aviram M. (2013). Pomegranate phytosterol (β-sitosterol) and polyphenolic antioxidant (punicalagin) addition to statin, significantly protected against macrophage foam cells formation. Atherosclerosis.

[592] Wang Z., Zhan Y., Xu J., Wang Y., Sun M., Chen J., Liang T., Wu L., Xu K. (2020). β-Sitosterol Reverses Multidrug Resistance via BCRP Suppression by Inhibiting the p53–MDM2 Interaction in Colorectal Cancer. J. Agric. Food Chem..

[593] El-Readi M.Z., Hamdan D., Farrag N., El-Shazly A., Wink M. (2010). Inhibition of P-glycoprotein activity by limonin and other secondary metabolites from Citrus species in human colon and leukaemia cell lines. Eur. J. Pharmacol..

[594] Beppe G.J., Dongmo A.B., Foyet H.S., Tsabang N., Olteanu Z., Cioanca O., Hancianu M., Dimo T., Hritcu L. (2014). Memory-enhancing activities of the aqueous extract of *Albizia adianthifolia* leaves in the 6-hydroxydopamine-lesion rodent model of Parkinson’s disease. BMC Complement. Altern. Med..

[595] Nakamura K., Deyama Y., Yoshimura Y., Hashimoto M., Kaga M., Suzuki K., Yawaka Y. (2011). Tannin-fluoride preparation attenuates prostaglandin E2 production by dental pulp cells. Mol. Med. Rep..

[596] Yao H.-T., Chang Y.-W., Lan S.-J., Yeh T.-K. (2008). The inhibitory effect of tannic acid on cytochrome P450 enzymes and NADPH-CYP reductase in rat and human liver microsomes. Food Chem. Toxicol..

[597] Karakurt S., Adali O. (2016). Tannic Acid Inhibits Proliferation, Migration, Invasion of Prostate Cancer and Modulates Drug Metabolizing and Antioxidant Enzymes. Anticancer Agents Med. Chem..

[598] Baer-Dubowska W., Szaefer H., Majchrzak-Celińska A., Krajka-Kuźniak V. (2020). Tannic Acid: Specific Form of Tannins in Cancer Chemoprevention and Therapy-Old and New Applications. Curr. Pharmacol. Rep..

[599] Liao X., Gao Y., Liu J., Tao L., Xie J., Gu Y., Liu T., Wang D., Xie D., Mo S. (2020). Combination of Tanshinone IIA and Cisplatin Inhibits Esophageal Cancer by Downregulating NF-κB/COX-2/VEGF Pathway. Front. Oncol..

[600] Zhang X.-X., Cao Y.-F., Wang L.-X., Yuan X.-L., Fang Z.-Z. (2017). Inhibitory effects of tanshinones towards the catalytic activity of UDP-glucuronosyltransferases (UGTs). Pharm. Biol..

[601] Chen F., Li L., Tian D.-D. (2017). *Salvia miltiorrhiza* roots against cardiovascular disease: Consideration of Herb-Drug Interactions. Biomed. Res. Int..

[602] Jing J., Zheng H., Wang J., Lin P., Zhang J., Xiong Z.-J., Wu Y.-Y., Ren J.-J., Yang H.-L., Wang X.-J. (2007). Growth inhibition and multidrug resistance-reversing effect of tanshinone I A on human breast cancer cell with estrogen receptor negative. Sichuan Da Xue Xue Bao Yi Xue Ban.

[603] Shu Q. (2013). Is Brain-derived Neurotrophic Factor a Possible Mechanism Underlying Risperidone Sensitization in Adolescent Rats?. Biochem. Pharmacol..

[604] Chen S., Jia J. (2020). Tenuifolin Attenuates Amyloid-β42-Induced Neuroinflammation in Microglia Through the NF-κB Signaling Pathway. J. Alzheimers. Dis..

[605] Gates S., Miners J.O. (1999). Cytochrome P450 isoform selectivity in human hepatic theobromine metabolism. Br. J. Clin. Pharmacol..

[606] Lin Y., Zhao W.R., Shi W.T., Zhang J., Zhang K.Y., Ding Q., Chen X.L., Tang J.Y., Zhou Z.Y. (2020). Pharmacological Activity, Pharmacokinetics, and Toxicity of Timosaponin AIII, a Natural Product Isolated From *Anemarrhena asphodeloides* Bunge: A Review. Front. Pharmacol..

[607] Kanaga Sabapathi S., Swamy V. (2015). Evaluation of cytochrome P450 inhibition properties of trigonelline hydrochloride by using Tandem Mass Spectrometry. World J. Pharm. Pharm. Sci..

[608] Isshiki M., Umezawa K., Tamura H. (2011). Coffee induces breast cancer resistance protein expression in Caco-2 cells. Biol. Pharm. Bull..

[609] Picking D., Chambers B., Barker J., Shah I., Porter R., Naughton D.P., Delgoda R. (2018). Inhibition of Cytochrome P450 Activities by Extracts of *Hyptis verticillata* Jacq.: Assessment for Potential HERB-Drug Interactions. Molecules.

[610] Jinhua W., Ying Z., Yuhua L. (2020). PXR-ABC drug transporters/CYP-mediated ursolic acid transport and metabolism in vitro and vivo. Arch. Pharm. (Weinheim).

[611] Singla R.K., Scotti L., Dubey A.K. (2017). In Silico Studies Revealed Multiple Neurological Targets for the Antidepressant Molecule Ursolic Acid. Curr. Neuropharmacol..

[612] Nabekura T., Yamaki T., Hiroi T., Ueno K., Kitagawa S. (2010). Inhibition of anticancer drug efflux transporter P-glycoprotein by rosemary phytochemicals. Pharmacol. Res..

[613] Hua W.J., Hua W.X., Nan F.Y., Jiang W.A., Yan C. (2014). The influence of herbal medicine ursolic acid on the uptake of rosuvastatin mediated by OATP1B1*1a and *5. Eur. J. Drug Metab. Pharmacokinet..

[614] Uesawa Y., Takeuchi T., Mohri K. (2012). Integrated analysis on the physicochemical properties of dihydropyridine calcium channel blockers in grapefruit juice interactions. Curr. Pharm. Biotechnol..

[615] Savai J., Pandita N., Chintamaneni M. (2014). Investigation of CYP1A interaction potential of *Withania somnifera* in rat and human liver microsomes. Indian J. Pharm. Sci..

[616] Wu Z., Uchi H., Morino-Koga S., Nakamura-Satomura A., Kita K., Shi W., Furue M. (2014). Z-Ligustilide inhibits benzo(a)pyrene-induced CYP1A1 upregulation in cultured human keratinocytes via ROS-dependent Nrf2 activation. Exp. Dermatol..

[617] Gonçalves B.M.F., Cardoso D.S.P., Ferreira M.-J.U. (2020). Overcoming Multidrug Resistance: Flavonoid and Terpenoid Nitrogen-Containing Derivatives as ABC Transporter Modulators. Molecules.

[618] Misaka S., Abe O., Sato H., Ono T., Shikama Y., Onoue S., Yabe H., Kimura J. (2018). Lack of pharmacokinetic interaction between fluvastatin and green tea in healthy volunteers. Eur. J. Clin. Pharmacol..

[619] Li M., Lan J., Li X., Xin M., Wang H., Zhang F., Lu X., Zhuang Z., Wu X. (2019). Novel ultra-small micelles based on ginsenoside Rb1: A potential nanoplatform for ocular drug delivery. Drug Deliv..

[620] González-Lugo O.E., Pozos-Guillén A., Ponce-Peña P., Lares-Asseff I., Escobar-García D.M., Campos-Cantón I., Vértiz-Hernández A.A. (2020). Synergistic interaction between 4-allyl-1-hydroxy-2-methoxybenzene (eugenol) and diclofenac: An isobolograpic analysis in Wistar rats. Drug Dev. Res..

[621] Matejczyk M., Ofman P., Dąbrowska K., Świsłocka R., Lewandowski W. (2020). The study of biological activity of transformation products of diclofenac and its interaction with chlorogenic acid. J. Environ. Sci..

[622] Bedada S.K., Yellu N.R., Neerati P. (2016). Effect of Resveratrol Treatment on the Pharmacokinetics of Diclofenac in Healthy Human Volunteers. Phytother. Res..

[623] Cheng X., Buckley D., Klaassen C.D. (2007). Regulation of hepatic bile acid transporters Ntcp and Bsep expression. Biochem. Pharmacol..

[624] Dawson S., Stahl S., Paul N., Barber J., Kenna J.G. (2012). In vitro inhibition of the bile salt export pump correlates with risk of cholestatic drug-induced liver injury in humans. Drug Metab. Dispos..

[625] DeBose-Boyd R.A. (2008). Feedback regulation of cholesterol synthesis: Sterol-accelerated ubiquitination and degradation of HMG CoA reductase. Cell Res..

[626] Sama V., Pagilla B., Chiluka R., Alvala R., Pola R.K., Mullangi R. (2019). Bioenhancing effects of naringin on atorvastatin. ADMET DMPK.

[627] Kong W., Wei J., Abidi P., Lin M., Inaba S., Li C., Wang Y., Wang Z., Si S., Pan H. (2004). Berberine is a novel cholesterol-lowering drug working through a unique mechanism distinct from statins. Nat. Med..

[628] Feng P., Zhao L., Guo F., Zhang B., Fang L., Zhan G., Xu X., Fang Q., Liang Z., Li B. (2018). The enhancement of cardiotoxicity that results from inhibitIon of CYP 3A4 activity and hERG channel by berberine in combination with statins. Chem. Biol. Interact..

[629] Li X., Xiao H., Lin C., Sun W., Wu T., Wang J., Chen B., Chen X., Cheng D. (2019). Synergistic effects of liposomes encapsulating atorvastatin calcium and curcumin and targeting dysfunctional endothelial cells in reducing atherosclerosis. Int. J. Nanomed..

[630] Bo L., Baosheng Z., Yang L., Mingmin T., Beiran L., Zhiqiang L., Huaqiang Z. (2016). Herb-drug enzyme-mediated interactions and the associated experimental methods: A review. J. Tradit. Chin. Med..

[631] NEUROTHERATM. https://klaire.com/nro-neurothera.

[632] Maxgars Memory Booster. https://www.maxgars.com/product/maxgars-memory-booster-60-capsules/.

[633] Palpu P., Rao C.V., Kishore K., Gupta Y.K., Kartik R., Govindrajan R. (2008). Herbal Formulation as Memory Enhancer in Alzheimer Condition. U.S. Patent.

[634] Shaw D., Graeme L., Pierre D., Elizabeth W., Kelvin C. (2012). Pharmacovigilance of herbal medicine. J. Ethnopharmacol..

