# Supplementary material for: Potential Herb–Drug Interactions in the Management of Age-Related Cognitive Dysfunction
Source: Pharmaceutics. 2021 Jan 19;13(1):124. doi: 10.3390/pharmaceutics13010124 (PMC7835864; doi:10.3390/pharmaceutics13010124)
Supplement: Supplementary file 1 [file pharmaceutics-13-00124-s001.pdf]

pharmaceutics

Supplementary Materials: Potential Herb–Drug Interactions in the Management of Age-Related Cognitive Dysfunction

Maria D. Auxtero, Susana Chalante, Mário R. Abade, Rui Jorge and Ana I. Fernandes

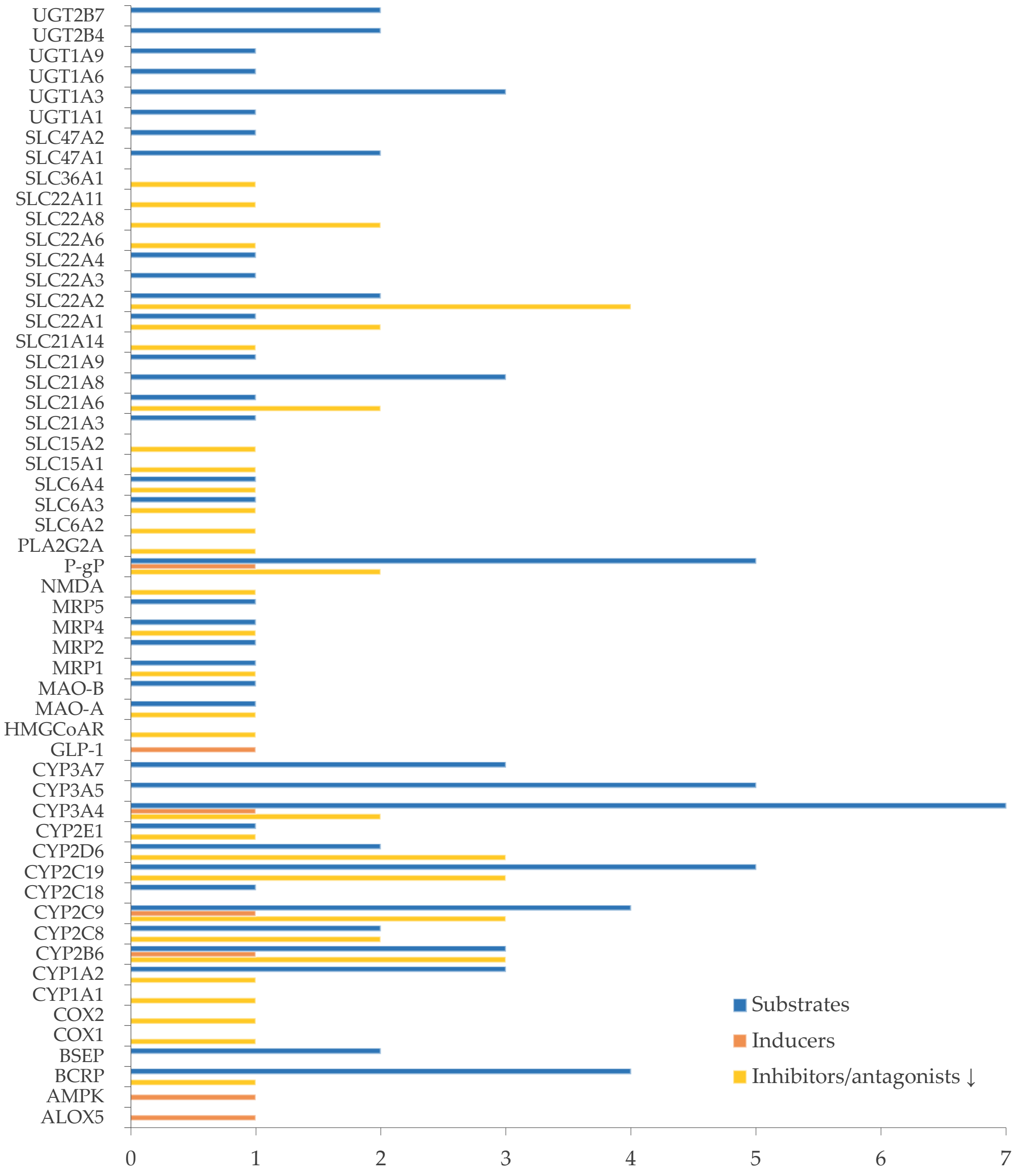

Figure S1. Number of drugs interacting with the different targets (enzymes, transporters and receptors) as substrates, inducers/upregulators and inhibitors/downregulators/antagonists.

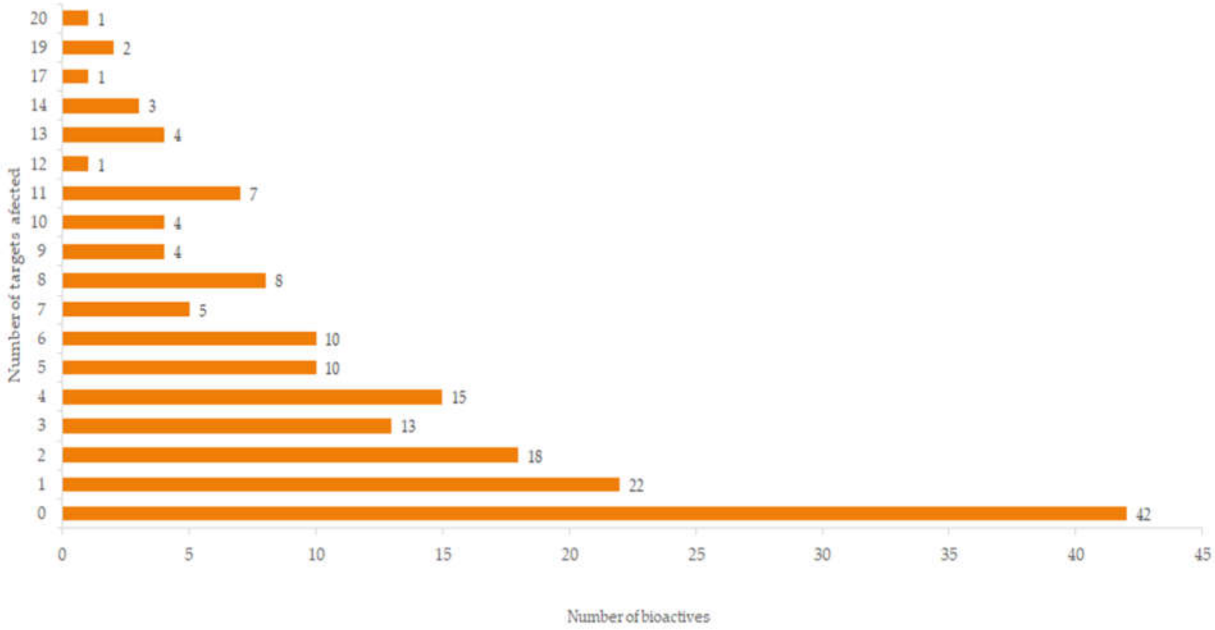

Figure S2. Frequency of target modulation by bioactives, as a measure of HDI potential.
